# Glutathione Mediates Control of Dual Differential Bio‐orthogonal Labelling of Biomolecules

**DOI:** 10.1002/anie.202313063

**Published:** 2023-11-13

**Authors:** Frederik Peschke, Andrea Taladriz‐Sender, Matthew J. Andrews, Allan J. B. Watson, Glenn A. Burley

**Affiliations:** ^1^ Department of Pure & Applied Chemistry & the Strathclyde Centre for Molecular Bioscience University of Strathclyde 295 Cathedral Street Glasgow G1 1XL UK; ^2^ EaStCHEM School of Chemistry University of Saint Andrews North Haugh St Andrews Fife KY16 9ST UK

**Keywords:** bio-orthogonal chemistry, ligation, CuAAC, peptide, oligonucleotide

## Abstract

Traditional approaches to bio‐orthogonal reaction discovery have focused on developing reagent pairs that react with each other faster than they are metabolically degraded. Glutathione (GSH) is typically responsible for the deactivation of most bio‐orthogonal reagents. Here we demonstrate that GSH promotes a Cu‐catalysed (3+2) cycloaddition reaction between an ynamine and an azide. We show that GSH acts as a redox modulator to control the Cu oxidation state in these cycloadditions. Rate enhancement of this reaction is specific for ynamine substrates and is tuneable by the Cu:GSH ratio. This unique GSH‐mediated reactivity gradient is then utilised in the dual sequential bio‐orthogonal labelling of peptides and oligonucleotides via two distinct chemoselective (3+2) cycloadditions.

## Graphical abstract

The chemical modification at precise sites within biomolecules is essential to assist in understanding their function. The incorporation of bio‐orthogonal reactive groups provides a strategy for selective tagging, however cross‐reactivity is problematic when dual modification is required. Glutathione (GSH) is used to control the reactivity of ynamine and cyclooctyne reagents in sequential (3+2) cycloaddition reactions.

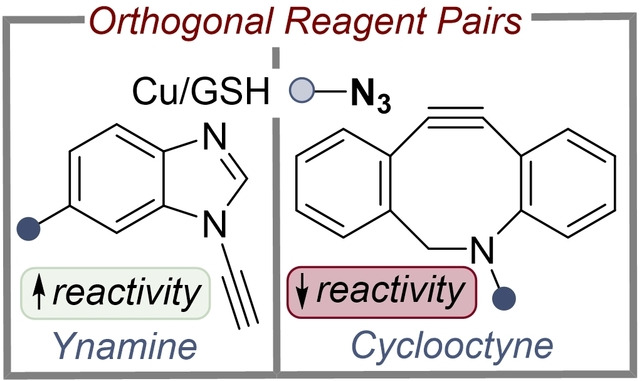

## Introduction

Orthogonal control of chemical reactivity is an essential requirement for the selective modification of biomolecules.[^1^, ^2^, ^3^] The “bio‐orthogonality” of these reactions is contingent on the choice of reagent pairs which selectively react with each other whilst minimising (or preferably abolishing) any cross‐reactivity with other available functional groups.[^4^, ^5^] Overcoming reagent deactivation by cellular components has been the main driver in bio‐orthogonal reaction design, which must be reconciled with the need for fast reaction kinetics of these pairs and high overall yields.[^6^, ^7^] This is particularly important for the preparation of bioconjugates where each reaction partner is a large, complex, and high‐value substrate.[^8^, ^9^]

**Figure 1:**
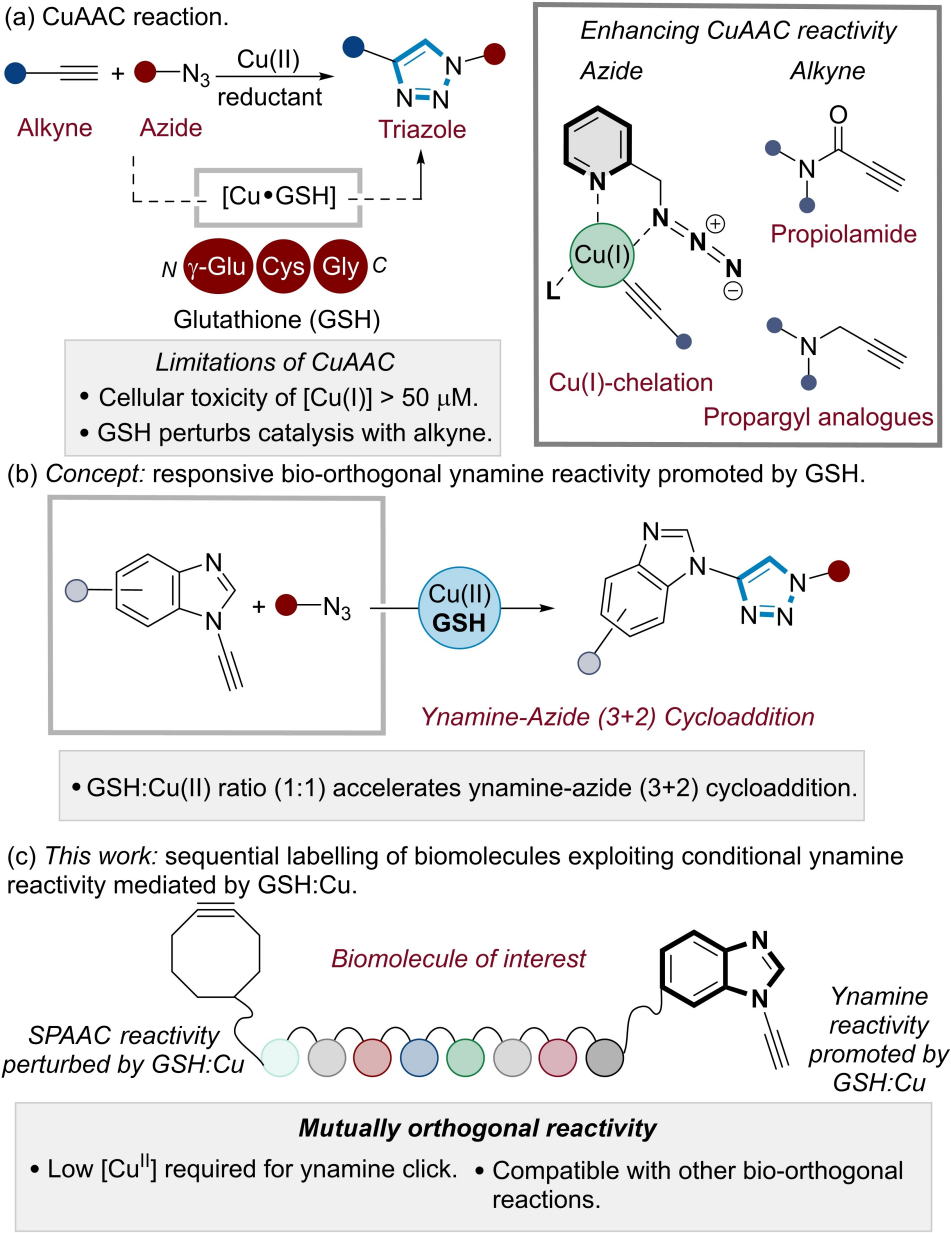
(a) CuAAC as a bio‐orthogonal tool in chemical biology. (b) Design concept: Controlling ynamine reactivity as a function of Cu:GSH ratio. (c) Sequential labelling of biomolecules exploiting conditional ynamine reactivity mediated by GSH:Cu.

In contrast to many of the metal‐free bio‐orthogonal reactions that have been developed,[^10^, ^11^] the copper‐catalysed azide‐alkyne cycloaddition (CuAAC) “click” reaction is unique in its reaction mechanism (Figure 1a). This is due to the acceleration of the reaction kinetics of the (3+2) cycloaddition by a copper catalyst to exclusively form a 1,4‐disubstituted triazole product (Figure 1a).[^12^, ^13^] As a result, a hallmark of the CuAAC reaction is the reactive latency of the azide and alkyne reagents under physiological conditions.^[14]^

This sets the CuAAC reaction apart from other classes of bio‐orthogonal reactions (e.g., strain‐promoted azide‐alkyne cycloadditions, SPAAC,[^15^, ^16^, ^17^] inverse electron demand Diels–Alder, iEDDA^[18]^), where the corresponding diene and dienophile need to react preferentially with each other in the presence of cellular components which are known to deactivate these functional groups, such as thiols present in cysteine residues.[^19^, ^20^, ^21^, ^22^] A particular case in point is glutathione (GSH), a tripeptide which is present in millimolar concentrations (0.1–10 mM) within a cellular environment (Figure 1a). GSH is the principle redox mediator in live cells, minimising the production of reactive oxygen species (ROS). However, this also causes perturbation of Cu catalysis in the CuAAC reaction when conventional alkynes are used.[^23^, ^24^] GSH acts as a chelating agent for Cu(II) as well as a reducing agent to form GSH−Cu(I) complexes.^[25]^ As a result, a significant limitation of the CuAAC in chemical biology workflows[^3^, ^26^, ^27^, ^28^] is the need for relatively high (micromolar) concentrations of Cu(I) for high conversions in aqueous buffered systems.[^28^, ^29^] This is problematic as Cu(I) is toxic to cells at these concentrations (e.g., 20–50 μM).[^30^, ^31^]

An additional issue is the prevalence of numerous Lewis basic groups present in biomolecules, which act as sites for Cu chelation, resulting in sequestration of Cu species. This poses additional issues relating to the onset of oxidative damage of biomolecules.[^32^, ^33^] As a result, there is a fine interplay between forming sufficient levels of a catalytically competent Cu(I) species for efficient CuAAC ligation versus the potential to induce deleterious side‐reactions.

From a mechanistic perspective, the need for high Cu loadings when conventional alkynes in the CuAAC reaction under physiologically‐relevant conditions arises from the formation of a Cu‐acetylide species, which is rate‐determining.[^34^, ^35^] Efforts to mitigate oxidative damage whilst improving reaction kinetics of the CuAAC reaction have primarily focused on developing water‐soluble Cu(I)‐stabilizing ligands,^[24]^ Cu nanoparticles,[^30^, ^36^] and Cu‐chelating azide groups.[^37^, ^38^, ^39^] Despite these innovations, few strategies have focused on addressing the core issue i.e., developing reactive alkyne substrates, which when combined with Cu‐stabilising ligands and Cu‐chelating azides, lower the need for high Cu loadings.^[40]^

We have recently identified aromatic ynamines (Figure 1a) as highly reactive alkyne substrates for CuAAC reactions.[^41^, ^42^, ^43^] Ynamines are unique alkyne analogues since their reaction kinetics are based on a shift in the rate determining step away from acetylide formation towards the azide ligation step.^[44]^ This lowers the Cu dependency of the alkyne substrate, enabling the (3+2) cycloaddition to proceed at a far lower Cu concentration relative to that required for other alkyne classes. We surmised that this deviation from the conventional reaction mechanism could provide opportunities to tune ynamine reactivity when incorporated into biomolecules, such as nucleic acids and peptides.

Herein, we disclose a chemoselective tagging strategy which exploits a ubiquitous endogenous cellular redox modulator (GSH) to control the kinetics of ynamine CuAAC reactions (Figure 1b). By controlling the reactivity of both the Ynamine CuAAC and the SPAAC reaction, GSH enables the preparation di‐functionalised oligodeoxyribonucleotide (ODN) and peptide bioconjugates with chemoselective control of the modification site (Figure 1c). This represents a strategy to modulate bio‐orthogonal reactivity and selectivity by exploiting endogenous cellular components to tune bio‐orthogonal reaction kinetics.

## Results and Discussion

**Figure 2:**
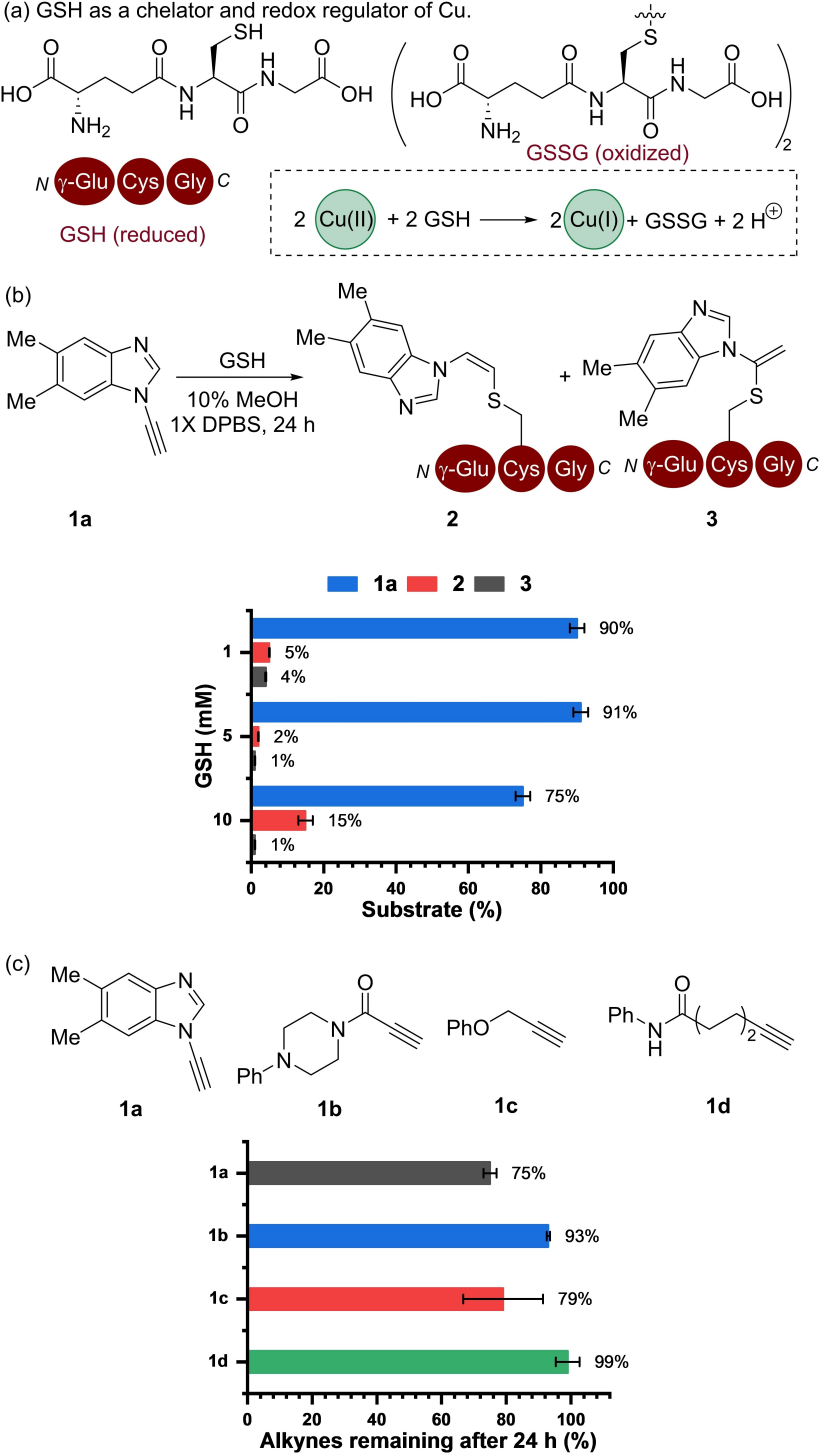
(a) GSH acts as a Cu ligand and redox mediator. (b) Formation of thio‐alkene adducts (**2**–**3**) by the reaction of ynamine (**1 a**, (200 μM) with GSH (1–10 mM) in Dulbecco's phosphate‐buffered saline (DPBS) containing 10 % methanol (MeOH). (c) Stability of **1 a**–**d** (200 μM) in the presence of GSH (10 mM). Error bars correspond to the standard deviation of three replicate experiments. All analyses conducted using reverse phase high pressure liquid chromatography (RP‐HPLC).

The first phase of the work focused on establishing a reactivity framework which considers the rate of the (3+2) cycloaddition alongside reagent deactivation in the presence of biologically relevant concentrations of GSH (Figure 2a). GSH is known to modulate the Cu oxidation state by acting as a chelating agent for Cu(II) as well as a reducing agent to form GSH−Cu(I) complexes.^[25]^ In addition, complexes are formed with primarily Cu(II) ions and glutathione disulphide (GSSG), in which Cu(II) chelation is influenced by the ratio of GSH/GSSG present.^[45]^

### Reaction Rate of Azide‐Ynamine (3+2) Cycloaddition is Influenced by Cu:GSH Ratio

We explored the stability of ynamine (**1 a**) across the physiological range of GSH^[46]^ in buffered solutions over 24 h (Figure 2b). In the presence of **1 a** and 5 mM of GSH, low levels (≈10 %) of ynamine **1 a** formed GSH adducts **2** and **3** (Figure 2b) either from a radical‐based thiol‐yne addition to form **2**,^[47]^ or via a step‐wise activation and subsequent nucleophilic addition to form **3**.[^48^, ^49^] The addition of Cu(OAc)_2_ (350 μM) only led to the formation of **2** (Figure S9).^[50]^ The stability of **1 a** was then compared with **1 b**–**d** using 10 mM GSH by determining the percentage of alkyne analogue remaining after 24 h by RP‐HPLC (Figure 2c). Around 75 % of **1 a** remained under these conditions. The stability of **1 a** was comparable to the propargyl ether (**1 c**) in which 79 % remained after 24 h. The most stable alkynes in this series were **1 b** and **1 d**, in which 93 % and 99 % remained after 24 h, respectively. Taken collectively, the stability of **1 a** in the presence of GSH over 24 h provided confidence that an ynamine could be used for bio‐orthogonal tagging in a cellular redox environment.

**Figure 3:**
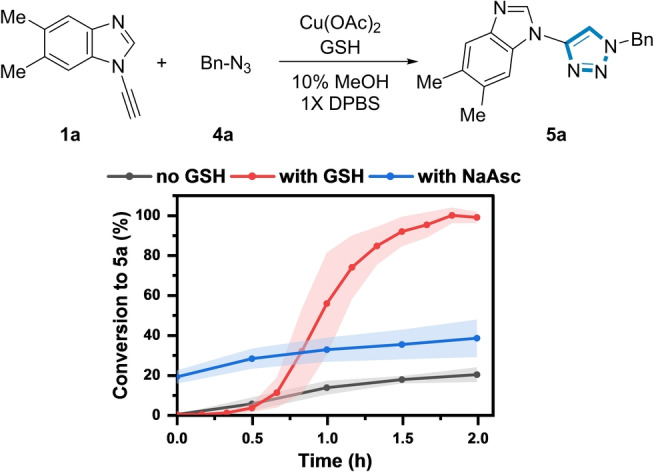
Influence of [GSH] and NaAsc on the formation of **5 a**. *Reaction conditions*: (black) **1 a** (200 μM), **4 a** (500 μM), Cu(OAc)_2_ (350 μM), 10 % MeOH, 1X DPBS, rt. (red)+GSH (1 mM). (blue)+NaAsc (1 mM). Shaded error bands correspond to the standard deviation of three replicate experiments.

The influence of [GSH] on the reaction kinetics was then explored using **1 a** and benzyl azide **4 a** as a model reagent pair to form **5 a** using 350 μM Cu(II) (Figure 3).

In the absence of GSH, the conversion to **5 a** was sluggish, reaching a maximum of only 20 % after 2 h. Unexpectedly, a rate acceleration was observed at 1 mM [GSH], resulting in full conversion to **5 a** at 2 h. However, a further increase in [GSH] prolonged the induction period (>8 h) to form **5 a** (Figure S10), indicating that the Cu:GSH ratio influenced the reaction kinetics. Potentially, the known ability of GSH to chelate Cu(II) at higher [GSH] could explain the slower reaction kinetics.[^51^, ^52^] Addition of 1 mM sodium ascorbate (NaAsc) instead of GSH only increased the conversion to **5 a** to 35 % after 2 h. Therefore, in stark contrast to other classes of alkyne (see below), at certain concentrations, GSH was a more effective reductant for the ynamine‐azide (3+2) cycloaddition than NaAsc, which has been traditionally used in conventional CuAAC reactions.

**Figure 4:**
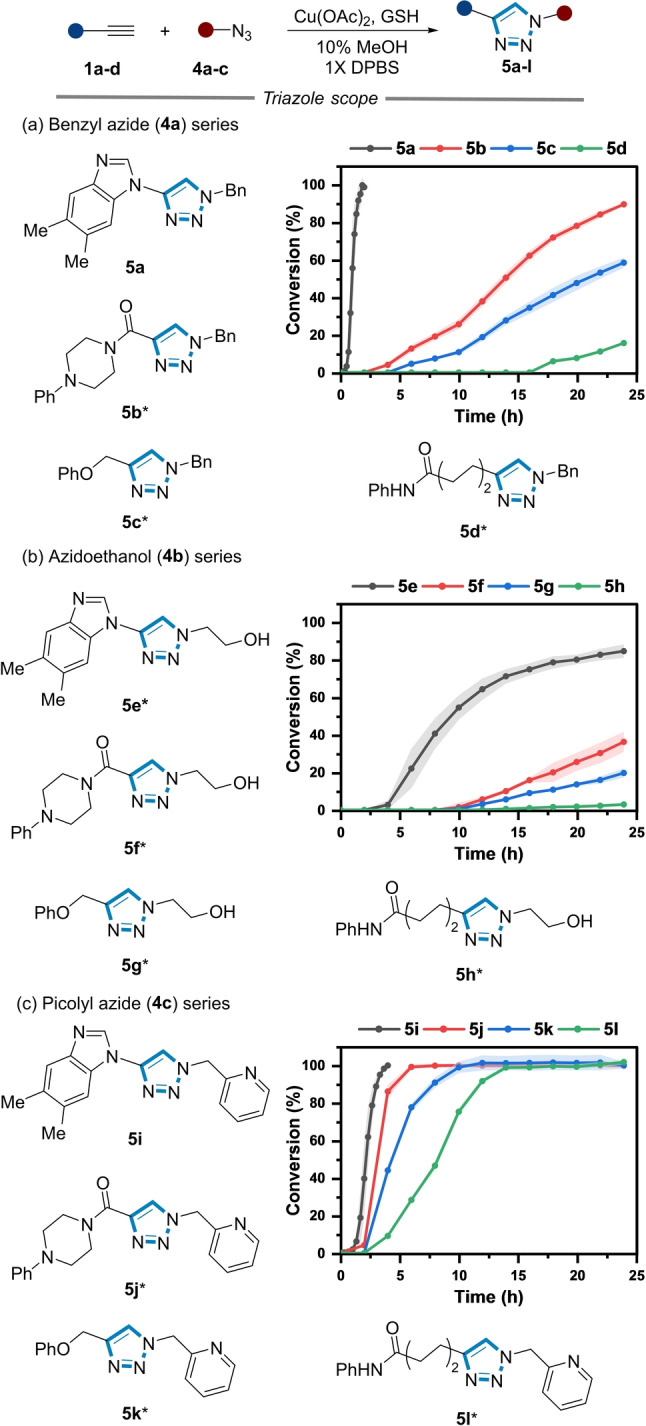
Reaction kinetics of triazole formation using benzyl azide. (a), azidoethanol (b), and picolyl azide (c). *Reaction conditions*: **1 a**–**d** (200 μM), **4 a**–**c** (500 μM), Cu(OAc)_2_ (350 μM), GSH (1 mM), 10 % MeOH, 1X DPBS, rt. * Addition of NaAsc (1 mM). Shaded error bands correspond to the standard deviation of three replicate experiments.

We then explored the reaction kinetics of a series of alkynes **1 a**–**d** under comparable conditions using azides **4 a**–**c** (Figure 4). Azides **4 a**–**c** were chosen based on their variation in physicochemical properties, and in the case of picolyl azide, their enhanced reactivity in the CuAAC reaction in physiological conditions.[^37^, ^38^] One striking observation was no product formation was observed using other classes of alkynes (i.e., **1 b**–**d**) when only GSH and benzyl azide (**4 a**) were used.

Products **5 b**–**d** were formed only upon the addition of NaAsc (1 mM) and NaAsc was also required across all three azides using alkynes **1 b**–**d** (data not shown).

When **4 a** was used as the corresponding azide, the rate of triazole formation was fastest for the ynamine substrate, forming **5 a** without the addition of NaAsc in 2 h (Figure 4a). The reaction of **4 a** with propiolamide **1 b**, an alkyne known to undergo facile CuAAC,^[40]^ was considerably slower, reaching 80 % conversion after 24 h. The reactions of **5 c**–**d** were poorer still, reaching 60 % and 20 % conversion when **1 c** and **1 d** were used after 24 h, respectively (Figure 4b).

When the water‐soluble azide **4 b** was used, the rate of triazole formation (**5 e**–**h**) was slower compared to that observed for the benzyl azide series. The stark reactivity difference of azide **4 b** compared to azide **4 a** might be attributed to an “on water” effect, which can accelerate cycloaddition reactions when hydrophobic substrates are used.[^53^, ^54^, ^55^] In this instance, the kinetics of the otherwise sluggish reaction (Figure S11) between **1 a** and **4 b** increased by the addition of NaAsc to form **5 b**, further suggestive of the influence of the physicochemical properties of the azide on the rate of triazole formation.

Finally, the reaction kinetics of the alkyne series were explored using picolyl azide **4 c**. The Cu‐chelating pyridyl unit of picolyl azides are more reactive in CuAAC reaction than regular azides under physiological conditions,^[38]^ which also was observed in our alkyne series. In contrast to the stark differences in reaction kinetics observed when **4 a** and **4 b** were used, full conversion was observed to products **5 i**–**l** across all three alkynes using **4 c** (Figure 4c).^[37]^ Consistent with the benzyl azide (Figure 4a), **1 b**–**d** required NaAsc to reach completion, which was not a requirement for **1 a**. Collectively, these data highlight the divergence of ynamine reactivity relative to other alkyne substrates in the CuAAC reaction, with GSH and the azide component further influencing the rate of product formation in physiologically relevant buffer systems.

### Reaction Optimisation of the Azide‐Ynamine (3+2) Cycloaddition Reaction

**Figure 5:**
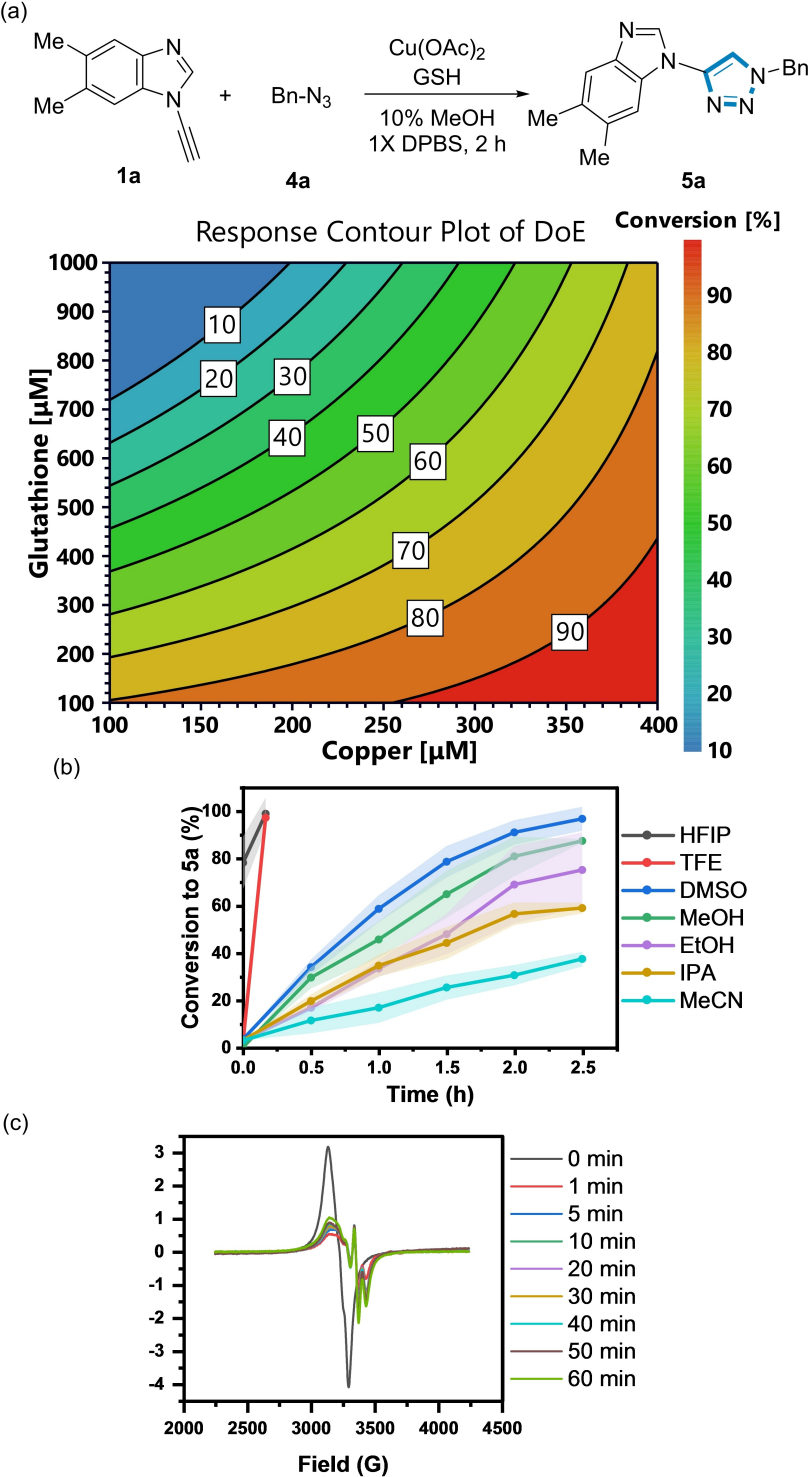
(a) DoE analysis as a function of conversion to triazole (**5 a**). *Reaction conditions*: **1 a** (200 μM), **4 a** (500 μM), Cu(OAc)_2_ (100–400 μM), GSH (100 μM‐1 mM), 10 % MeOH in 1X DPBS, rt. (b) Organic co‐solvent effects on the formation of **5 a**. *Reaction conditions*: **1 a** (200 μM), **4 a** (500 μM), Cu(OAc)_2_ (100 μM), GSH (100 μM), 1X DPBS in 10 % organic co‐solvent. Shaded error bands correspond to the standard deviation of three replicate experiments. (c) EPR spectrum of the ynamine‐azide (3+2) cycloaddition in HFIP:H_2_O (1 : 9).

We then explored how the GSH:Cu(II) ratio and the organic co‐solvent influenced the reaction kinetics of the ynamine‐azide (3+2) cycloaddition. Design of Experiments (DoE) was used to survey the variable space using a central composite face two‐factor design (three levels).^[56]^ A [Cu(II)] range of 100 μM, 250 μM, and 400 μM was used relative to [GSH] at 100 μM, 550 μM, and 1 mM. Using this DoE strategy, an optimal GSH:Cu(II) ratio of 1 : 1 (best reactivity at the lowest [Cu]) to form **5 a** was observed without causing a measurable lag period (Figure 5a and Figure S12). A pertinent observation was an induction period occurs when the GSH:Cu(II)>2 : 1. No reaction was observed when GSH:Cu(II) was 3 : 1 after 2.5 h under these conditions (Figure S12).^[23]^ No difference was observed when CuSO_4_ was used as a Cu source relative to Cu(OAc)_2_ (Figure S13).

Previous studies have shown that Diels–Alder reaction kinetics are enhanced using protic fluorinated solvents.[^57^, ^58^, ^59^] However, their application as a co‐solvent in CuAAC has not been explored in depth.[^23^, ^40^, ^60^, ^61^, ^62^] A co‐solvent screen (10 % organic co‐solvent in 1X DPBS buffer) revealed a striking increase in both the reaction rate and conversion to **5 a** when trifluoroethanol (TFE) or hexafluoroisopropanol (HFIP) was used (Figure 5b). A pertinent comparator is the observed differences in conversion to **5 a** using HFIP (full conversion in 10 min) relative to IPA (≈60 % conversion after 2.5 h), suggesting the enhanced polarity and H‐bond donating character of fluorinated solvents enhances the CuAAC reaction kinetics. Electron Paramagnetic Resonance (EPR) was used to determine the influence of the GSH:Cu ratio on the Cu oxidation state and the co‐solvent.[^25^, ^51^, ^63^, ^64^] Complete reduction of Cu(II) to Cu(I) was observed at a GSH:Cu ratio >3 : 1, whereas only partial reduction with a GSH:Cu ratio of <3 : 1 in a 10 % MeOH/H_2_O mixture (Figure S20).

Under the optimised reaction conditions (GSH:Cu=1 : 1), only partial reduction occurred in the first 1 min of the reaction in both HFIP (Figure 5c) and MeOH (Figure S20). This signal resembles the EPR spectrum of GSSG+Cu(OAc)_2_ (Figure S19), which suggests the formation of a Cu(II)‐GSSG complex^[52]^ and the presence of mixed Cu oxidation states during the reaction.[^37^, ^65^] This HFIP phenomenon was also observed using **1 d** with benzyl azide **4 a** (Figure S14).

### Dual Differential Modification of Peptides and DNA Exploiting Conditional Reactivity of the Azide‐Ynamine (3+2) Cycloaddition

**Figure 6:**
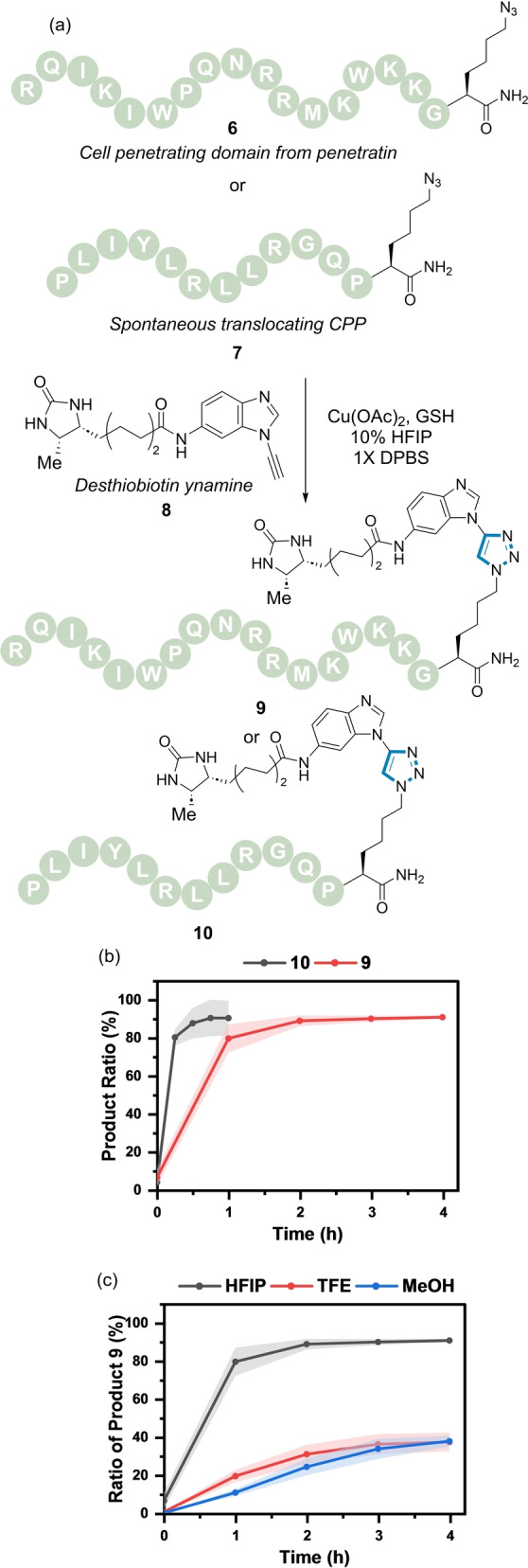
(a) CuAAC reaction of azide‐modified CPPs (**6**–**7**) and ynamine (**8**) catalysed by Cu(OAc)_2_ and GSH. (b) Reaction profile of the formation of **9** (red) and **10** (grey). *Reaction conditions*: **8** (200 μM), **6/7** (200 μM), Cu(OAc)_2_ (100 μM), GSH (100 μM), 10 % HFIP in 1X DPBS, rt. (c) Reaction profile of the formation of **9** using different co‐solvents (10 % co‐solvent) in 1X DPBS. Shaded error bands correspond to the standard deviation of three replicate experiments.

The GSH‐dependent reactivity of the ynamine in the CuAAC reaction offered a new concept for chemoselective biomolecule ligation. This was explored in the post‐synthetic modification of cell‐penetrating peptides (CPPs) and ODNs. Peptide **6** is based on a CPP sequence derived from the third helix homeodomain of Antennapedia (i.e., penetratin),^[66]^ whereas **7** is derived from a previously identified spontaneous membrane translocating peptide (Figure 6a).^[67]^ CPPs **6** and **7** were prepared by solid phase synthesis with an azido lysine installed at the C‐terminus. A desthiobiotin‐modified ynamine **8** was used as the corresponding reaction partner.

The formation of triazoles **9** and **10** using optimised conditions involving the use of 10 % HFIP in 1X DPBS buffer was explored (Figure 6b). Using these conditions, maximal conversion was observed within 2–4 h. Figure 6c highlights influence of the co‐solvent on the reaction with only ≈40 % conversion to **9** observed when TFE or MeOH was used as a co‐solvent compared with ≈90 % conversion using HFIP.

Previous work by Hosoya et al. has shown that transient protection of the internal alkyne of dibenzocyclooctyne (DBCO) occurs in the presence of Cu(I).^[68]^ Addition of a Cu chelator such as ethylenediaminetetraacetic acid (EDTA) results in decomplexation and reestablishes the reactivity of the cyclooctyne group to undergo SPAAC.^[69]^ We surmised that reduction of Cu(II) to Cu(I) by GSH would lead to transient protection of the DBCO triple bond. Conversely, in the absence of GSH only the SPAAC reaction would proceed, forming DBCO‐based triazoles.

The concept of GSH as an ynamine reactivity modulator was demonstrated by a competition experiment using azide **6** in the presence of DBCO analogue **11** and ynamine **8** (Figure S15). In the absence of GSH, exclusive formation of the SPAAC adducts **S11a/b** were observed (Figure S15b), whereas in the presence of GSH, ynamine based triazole **9** was formed selectively (Figure S15c).

These results prompted us to explore the chemoselectivity of the dual modification of biomolecules. The ability to control the sequence and site of two modifications provides a step‐efficient methodology to tune the properties of bioconjugates.[^4^, ^7^, ^70^, ^71^] Picolyl azides are known to undergo CuAAC reactions preferentially in the presence of an aliphatic azide;[^37^, ^43^] enabled by its capacity to chelate a Cu atom.^[38]^ Thus we hypothesised that a sequential one‐pot click reaction on doubly azide‐modified peptides with ynamine **8** and DBCO **11** would be possible by exploiting the difference in azide reactivity and the transient protection of DBCO **11** by Cu(II) reduction with GSH.

**Figure 7:**
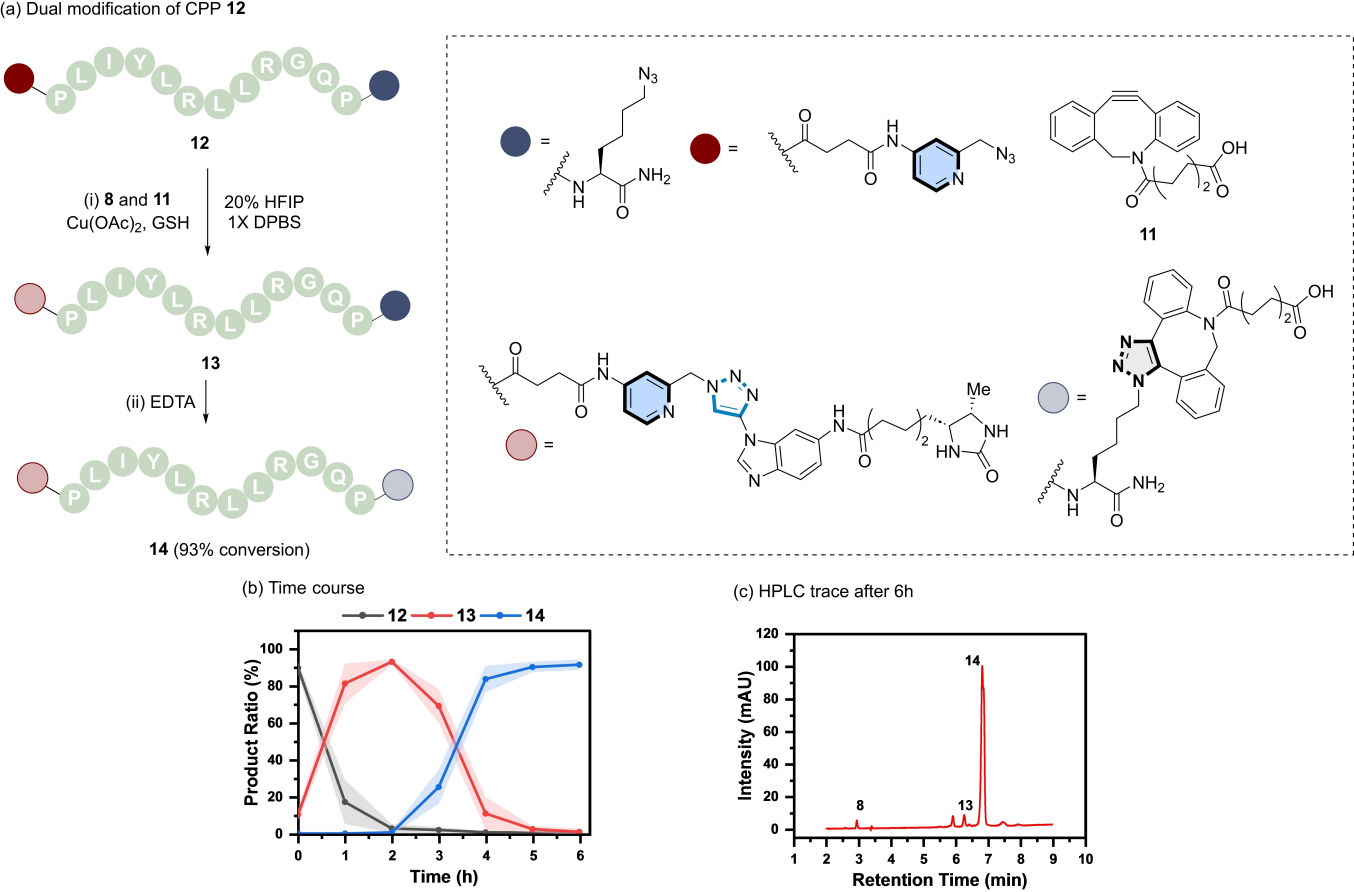
(a) Sequential and selective modification of a double azide labelled CPP (**12**). *Reaction conditions*: (i) **12** (200 μM), **8** (220 μM), **11** (220 μM), Cu(OAc)_2_ (500 μM), GSH (500 μM), 20 % HFIP in 1X DPBS, rt, 2 h; (ii) EDTA (5 mM), 4 h. (b) Reaction profile of the formation of mono‐functionalised CPP (**13**, red), followed by the di‐functionalised CPP (**14**, blue) from **12** (black). Shaded error bands correspond to the standard deviation of three replicate experiments. (c) RP‐HPLC trace of the reaction mixture after 6 h.

To explore the scope of aromatic ynamines as a tool for selective dual modification, the doubly modified CPP **12** (derived from **7**) was prepared by incorporation of a Cu‐chelating azide at the N‐terminus via solid phase synthesis (Figure 7a).

Dual azide‐labelled peptide **12** was then tested in a one‐pot chemoselective peptide modification strategy in the presence of equimolar amounts of **8** and analogue **11** using similar reaction conditions applied previously to the mono‐labelling of peptides (Figure 6). However, ynamine **8** proved to be too reactive under these conditions and reacted indiscriminately with both the picolyl and aliphatic azides. This was improved by changing the concentration of the co‐solvent HFIP. The reaction rate was correlated with the solvent concentration and decreased with increasing amount of HFIP (Figure S16).

The differences in reactivity of ynamine **8** as a function of HFIP concentration accentuated the reactivity difference between the two azides and resulted in an increase in the chemoselectivity for picolyl azide. The use of 20 % HFIP resulted in the best compromise between reactivity and chemoselectivity.

“Deprotection” of the strained alkyne triple bond was accomplished by addition of excess EDTA after 2 h and invoked the reactivity of the DBCO group upon decomplexation with Cu(I).^[68]^ This step enabled the second (3+2) cycloaddition to occur between the azido lysine on peptide **12** and DBCO **11**. RP‐HPLC analysis of the sequential modification of peptide **12** revealed the formation of the mono‐labelled triazole peptide **13** from **12** first (Figure 7b), followed by the formation of the dual‐labelled peptide **14** in 93 % (Figure 7c and Figure S21). Enzymatic digestion confirmed the CuAAC of ynamine **8** occurred exclusively at the *N*‐terminal picolyl azide under these conditions (Figure S22). Taken collectively, these results show the mutual orthogonality of the aromatic ynamine group in the presence of a redox modulator (i.e., GSH).

**Figure 8:**
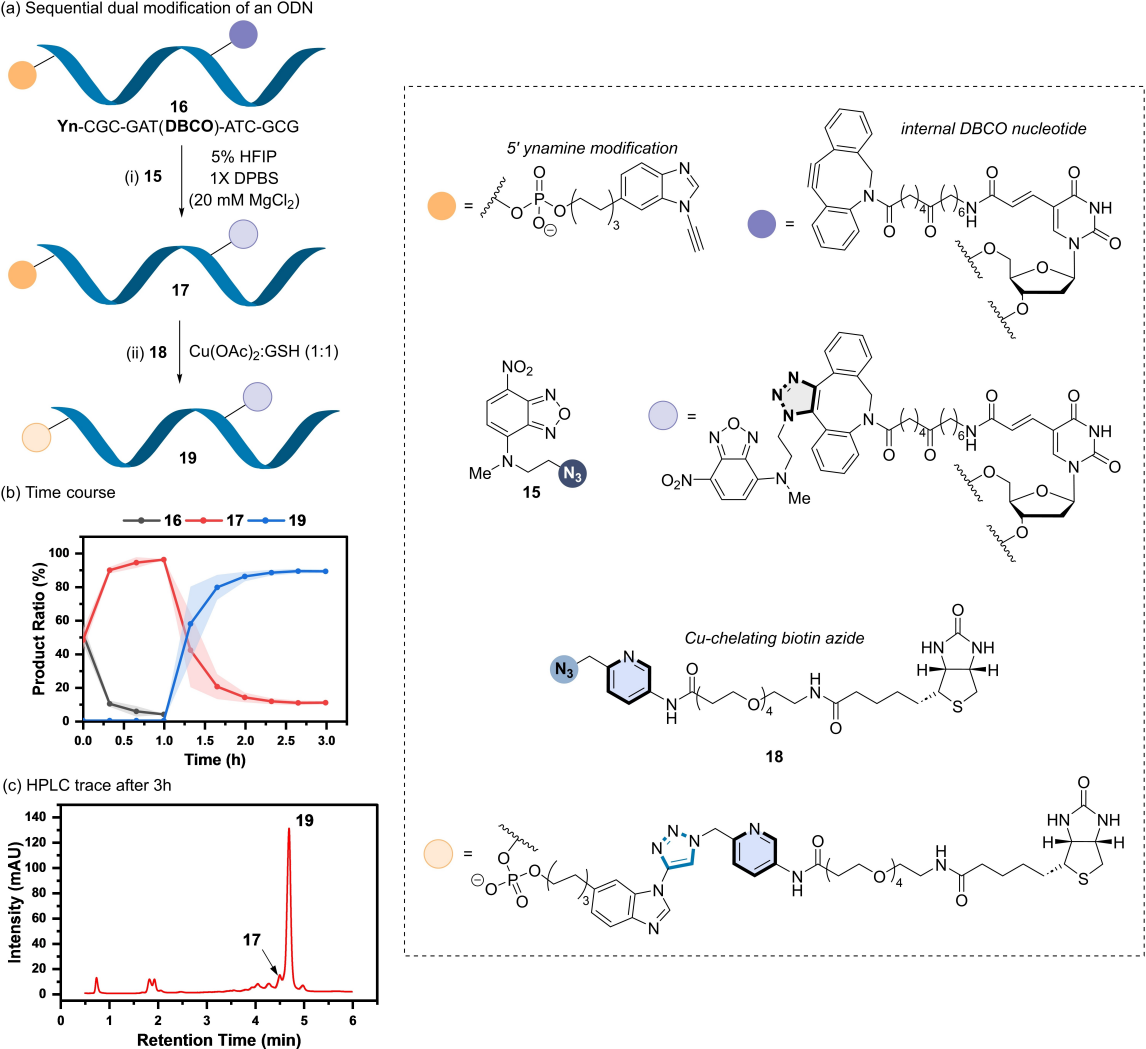
(a) Sequential modification of a dual‐alkyne modified ODN (**16**). *Reaction conditions*: (i) **16** (20 μM), **15** (20 μM), 5 % HFIP in 1X DPBS (20 mM MgCl_2_), rt, 1 h; (ii) **18** (30 μM), Cu(OAc)_2_ (50 μM), GSH (50 μM), 2 h. (b) RP‐HPLC time course of the sequential, dual modification of **16** to form **19** via **17**. Shaded error bands correspond to the standard deviation of three replicate experiments. (c) RP‐HPLC trace of the reaction mixture after 3 h.

Finally, the versatility of the ynamine platform as a tuneable bio‐orthogonal reactive group in biomolecule labelling was extended to the post‐synthetic modification of ODNs.[^72^, ^73^, ^74^] Installation of an ynamine onto the 5′‐end of the dodecamer ODN **S10** was achieved by automated solid phase synthesis using the ynamine phosphoramidite **S9** (Figure 8a). Fluorescent azide **15** was used as the reactive partner. Using the GSH‐based reaction conditions based on the labelling of peptides, 90 % conversion of **S10** into product **S12** was observed (Figure S17b).

A chemoselective, dual modification approach was then explored using ODN **16**, which contains an ynamine incorporated on the 5′ end, and a DBCO group linked to an internal T nucleotide (Figure 8). In the absence of Cu(II) and GSH, exclusive formation of the mono‐adduct ODN **17** was observed, where the (3+2) cycloaddition takes place at the DBCO sited using azide **15** after 1 h (Figure 8a/b). Subsequent addition of Cu(II):GSH (1 : 1) and azide **18** then provided the dual functionalised product **19** in ≈90 % conversion (Figure 8c). The chemoselectivity of each (3+2) cycloaddition was confirmed by enzymatic digestion (Figure S23), highlighting the ability to tune the reactivity of an ynamine and cyclooctyne by the choice of reaction conditions when incorporated into an ODN.

## Conclusion

We have shown that the reaction kinetics of ynamine CuAAC is modulated by the endogenous cellular redox regulator GSH, with further fine‐tuning of kinetics possible by using a fluorinated solvent, such as HFIP.

This allows robust control of ynamines as CuAAC reagents by modulating the Cu redox state,^[70]^ and, importantly, enables ynamines to undergo CuAAC in the presence of GSH while the reactivity of other alkynes is suppressed.

This work highlights the ability to exploit the redox properties of GSH rather than minimising the deactivation of existing reagents in the bio‐orthogonal toolkit. Further fine‐tuning of ynamine reactivity in the presence of a Cu catalyst and reactive oxygen species (e.g., H_2_O_2_) could widen the aperture of applications of this bio‐orthogonal reactive group.^[75]^ Moreover, we show the biocompatibility of the ynamine reactive group and chemoselective control of biomolecule tagging in concert with SPAAC reagents. Whilst this strategy requires the dedicated synthesis of ynamine building blocks and their incorporation into peptides and ODNs by solid phase synthesis, this complements other reagents in the bio‐orthogonal reaction toolkit. As a result, defining “reactive orthogonality” amongst bio‐orthogonal reagents^[68]^ opens up opportunities for the step‐efficient preparation of bioconjugates, and potentially extending this conditional reactivity to the selective labelling of biomolecules within live cells.^[76]^

## Abbreviations

CuAAC: Cu‐catalysed alkyne‐azide (3+2) cycloaddition
CPP: cell‐penetrating peptide
DBCO: dibenzocyclooctyne
DPBS: Dulbecco's phosphate‐buffered saline
DoE: design of experiments
EDTA: ethylenediaminetetraacetic acid
EPR: electron paramagnetic resonance
GSH: glutathione
GSSG: glutathione disulphide
HFIP: hexafluoroisopropyl alcohol
IEDDA: inverse electron demand Diels–Alder
MeOH: methanol
NaAsc: sodium ascorbate
ODN: oligodeoxyribonucleotide
ROS: reactive oxygen species
RP‐HPLC: reverse phase high pressure liquid chromatography
SPAAC: strain‐promoted azide‐alkyne cycloaddition
TFE: trifluoroethanol

## Supporting Information

The authors have cited additional references within the Supporting Information.[^44^, ^77^, ^78^, ^79^, ^80^, ^81^, ^82^, ^83^]

## Author Contributions

The manuscript was written through contributions of all authors. All authors have given approval to the final version of the manuscript.

## Conflict of interest

A patent application relating to the work in this manuscript (GB2209210.0 PG450228GB).

1

## Supporting information

As a service to our authors and readers, this journal provides supporting information supplied by the authors. Such materials are peer reviewed and may be re‐organized for online delivery, but are not copy‐edited or typeset. Technical support issues arising from supporting information (other than missing files) should be addressed to the authors.

Supporting Information

## Data Availability

The data that support the findings of this study are available in the supplementary material of this article.
