# Supplementary material for: Glutathione Mediates Control of Dual Differential Bio‐orthogonal Labelling of Biomolecules
Source: Angew Chem Int Ed Engl. 2023 Nov 13;62(50):e202313063. doi: 10.1002/anie.202313063 (PMC10952886; doi:10.1002/anie.202313063)
Supplement: Supplementary file 1 — Supporting Information [file ANIE-62-0-s001.pdf]

Angewandte Chemie
Eine Zeitschrift der Gesellschaft Deutscher Chemiker

Supporting Information

Glutathione Mediates Control of Dual Differential Bio-orthogonal Labelling of Biomolecules

F. Peschke, A. Taladriz-Sender, M. J. Andrews, A. J. B. Watson, G. A. Burley**

Contents

Contents 1
1. General Experimental Techniques and Procedures 2
1.1 Small Molecule Synthesis 3
1.2 General Protocol Peptide Synthesis 17
1.3 General Protocol for Oligonucleotide Synthesis 20
2. HPLC Assay Protocol 22
2.1 Procedure for Small Molecule HPLC Assay 22
2.2 Procedure for Peptide HPLC Assay 24
2.3 Procedure for Oligonucleotide HPLC Assay 28
3. Supplementary Figures 33
3.1 HPLC data 33
3.2 EPR-Data 38
3.3 Supplementary UPLC-MS Data 1
4. Experimental Spectra 1
5. List of References 29

1. General Experimental Techniques and Procedures

All reagents and solvents were purchased from commercial sources and used without further purification (except for benzyl azide which was purchased from Fluorochem (Cat Num: 342017) and purified by column chromatography). 1X DPBS buffer was purchased from Sigma-Aldrich (SKU: D8662-100ML). Thin layer chromatography (TLC) was carried out using Merck silica plates coated with fluorescent indicator UV254. TLC plates were analysed under 254 nm UV light or developed using potassium permanganate solution. Data analysis was performed in Excel and data plotted with OriginPro®.

Column Chromatography (normal-phase flash chromatography) was carried out using Silicagel 60A 40-63 µm obtained from Fluorochem (Cat No: LC401). Ethyl acetate in petroleum ether 40 – 60 °C or MeOH in DCM were commonly used as eluent mixtures. The exact conditions are reported in the experimental information for each compound.

NMR spectra (^{1}H and ^{13}C NMR) were obtained using Bruker spectrometers (400, 500 or 600 MHz). All chemical shifts (δ) were referenced at 7.26 (^{1}H) and 77.06 ppm (^{13}C) in $CDCl_3$, at 2.50 (^{1}H) and 39.52 (^{13}C) in $(CD_3)_2SO$, at 4.79 ppm (^{1}H) in D_2O and at 3.31 (^{1}H) and 49.0 ppm (^{13}C) in CD_3OD. Coupling constants are reported in hertz (Hz) and multiplicities are abbreviated as follows: s = singlet, d = doublet, t = triplet, q = quartet and m = multiplet.

EPR Spectroscopy was carried out on a Bruker EMX plus spectrometer controlled by a Bruker ER 083 CS microwave bridge operating at X-Band, microwave frequency of ≈ 9.9 GHz at microwave power of 20.70 mW, modulation amplitude of 10.0 G and a time constant of 40.96 s. The sweep centre was at 3425 G and a sweep width of 2000 G with 2000 points on X-axis.

Low Resolution Mass Spectrometry was used for compound identification and reaction monitoring. LC-MS was carried out on an Agilent HPLC instrument in equipped with an Agilent Quadrupole mass detector on an Agilent Poroshell 120 C18 column (75 mm × 4.6 mm, 2.7 µm). UPLC-MS was carried out on an AVANT UPLC equipped with an Advion Expression CMS L (ESI) on a Phenomenex Kinetex C18 column (30 mm × 2.1 mm, 2.7 µm).

Infrared Spectroscopy (IR) was carried out on an Agilent or Shimadzu FTIR spectrometer and the data processed using the proprietary software. Only major absorbances were reported.

High Resolution Mass Spectrometry (HRMS) was carried out an LTQ Orbitrap XL 1 mass spectrometer at the EPSRC UK National Mass Spectrometry Facility (Swansea), or a Thermo Exactive Orbitrap mass spectrometer at the University of St Andrews or a Bruker Micro TOF II at the university of Edinburgh.

MALDI-ToF mass spectra were recorded using a Shimadzu Axima Confidence spectrometer, using 3-hydroxypicolinic acid (HPA) and ammonium citrate as the matrix. Spectra were recorded in positive mode (linear or reflectron). For details see relevant experiments.

1.1 Small Molecule Synthesis

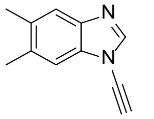

1-ethynyl-5,6-dimethyl-1*H*-benzo[*d*]imidazole (1a)[1]

5,6-Dimethyl-1-((triisopropylsilyl)ethynyl)-1H-benzo[d]imidazole[2] (4.0 g, 12.5 mmol) was dissolved in acetonitrile (62 mL), fluoride on polymer (415 mg, 12.5 mmol, Sigma catalogue number: 387789-10G) was added and the reaction was stirred at room temperature overnight. Then the reaction mixture was filtered to remove the polymer, and the filtrate was concentrated under vacuum. The residue was purified by flash column chromatography (0 – 20% ethyl acetate in petroleum ether) and the desired product was obtained as a white solid (1.99 g, 94%).

^{1}H NMR (500 MHz, $CDCl_3$) δ 7.98 (s, 1H), 7.56 (s, 1H), 7.35 (s, 1H), 3.26 (s, 1H), 2.41 (s, 3H), 2.38 (s, 3H).

^{13}C NMR (126 MHz, $CDCl_3$) δ 144.0, 143.6, 143.1, 128.2, 114.4, 111.3, 105.7, 90.6, 72.4, 18.7, 11.3.

IR (ν_{max}): 3195 (sp C-H), 3107 (ar. C-H), 2148 (C≡C), 1492 (ar. C=C), 1460 (ar. C=C) cm^{-1}.

NMR spectra in agreement with literature values.[1]

***N*^{5}-((*R*)-1-((carboxymethyl)amino)-3-(((*Z*)-2-(5,6-dimethyl-1*H*-benzo[*d*]imidazol-1-yl)vinyl)thio)-1-oxopropan-2-yl)-*L*-glutamine (2) and *N*^{5}-((*R*)-1-((carboxymethyl)amino)-3-((1-(5,6-dimethyl-1*H*-benzo[*d*]imidazol-1-yl)vinyl)thio)-1-oxopropan-2-yl)-*L*-glutamine (3)**

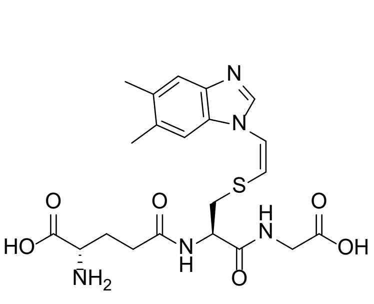

2

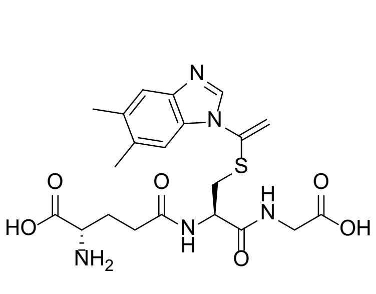

3

1-Ethynyl-5,6-dimethyl-1H-benzo[d]imidazole (20 mg, 0.12 mmol) and glutathione (370 mg, 1.2 mmol) were dissolved in a mixture of acetonitrile and 0.2 M sodium phosphate buffer. The reaction was stirred at room temperature for 2 weeks. Then water and diethyl ether were added, and phases were separated. The aqueous layer was washed again with diethyl ether, then the aqueous layer was evaporated under vacuum, and the residue purified by semi-preparative HPLC to yield **2** (6 mg, 10%) and **3** (1 mg, 2%) as a white solid.

Compound **2**: ^{1}**H NMR** (500 MHz, D_2O) δ 9.24 (s, 1H), 7.63 (s, 1H), 7.52 (s, 1H), 7.19 (d, *J* = 7.6 Hz, 1H), 6.98 (d, *J* = 7.6 Hz, 1H), 4.66 (dd, *J* = 8.3, 5.5 Hz, 1H), 3.94 (s, 2H), 3.79 (t, *J* = 6.5 Hz, 1H), 3.43 (dd, *J* = 14.5, 5.5 Hz, 1H), 3.23 (dd, *J* = 14.5, 8.3 Hz, 1H), 2.47 – 2.41 (m, 8H), 2.03 (dd, *J* = 14.3, 7.3 Hz, 2H).

^{13}**C NMR** (151 MHz, D_2O) δ 174.4, 173.1, 171.7, 138.0, 137.7, 129.0, 118.1, 114.2, 113.0, 112.4, 53.5, 41.4, 35.3, 31.3, 25.9, 19.7, 19.5. (2 quat. C not observed).

HRMS (ESI): *m/z* calcd for $C_{21}H_{26}N_5O_5S$: 476.1609 $[M\text{-}H]^-$; found: 476.1611.

Compound **3:** ^{1}**H NMR** (500 MHz, D_2O) δ 9.39 (s, 1H), 7.85 (s, 1H), 7.70 (s, 1H), 6.14 (d, *J* = 2.4 Hz, 1H), 6.07 (d, *J* = 2.4 Hz, 1H), 4.61 – 4.54 (m, 1H), 3.92 (s, 2H), 3.83 (d, *J* = 6.5 Hz, 1H), 3.16 (dd, *J* = 14.7, 5.5 Hz, 1H), 2.99 (dd, *J* = 14.7, 8.1 Hz, 1H), 2.53 – 2.44 (m, 9H), 2.22 – 2.08 (m, 2H).

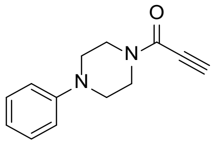

1-(4-phenylpiperazin-1-yl)prop-2-yn-1-one (1b)

Propiolic acid (500 mg, 7.14 mmol) was dissolved in MTBE (10 mL) under an argon atmosphere, and oxalyl chloride (1.09 g, 8.57 mmol) was added dropwise followed by 2 drops of DMF. The reaction was left stirring for 1.5 h and then added dropwise to a mixture of 1-phenylpiperazine (1.39 g, 8.57 mmol) and DIPEA (4.61 g, 35.7 mmol) in MTBE (5 mL). The reaction was stirred for an additional 1.5 h and then quenched with a saturated solution of $NaHCO_3$ (30 mL). The phases were separated, and the aqueous phase was extracted with ethyl acetate (20 mL). The combined organic layers were washed with water (2 × 30 mL), brine (30 mL) and then dried over Na_2SO_4. The solvents were removed under vacuum and the residue purified by column chromatography (25 – 30% ethyl acetate in petroleum ether) to give the desired product as a brown solid (271 mg, 18%).

^{1}**H NMR** (500 MHz, $CDCl_3$) δ 7.29 (dd, *J* = 8.7, 7.2 Hz, 2H), 6.97 – 6.89 (m, 3H), 3.93 (app. t, *J* = 5.0 Hz, 2H), 3.81 (app. t, *J* = 5.0 Hz, 2H), 3.22 (app. t, *J* = 5.2 Hz, 2H), 3.17 (app. t, *J* = 5.2 Hz, 2H), 3.15 (s, 1H).

^{13}**C NMR** (126 MHz, $CDCl_3$) δ 151.8, 150.8, 129.3, 120.9, 117.0, 79.5, 75.3, 50.0, 49.3, 46.9, 41.4.

IR (ν_{max}**)**: 3205 (ar C-H), 2917 (C-H), 2103 (C≡C), 1622 (C=O) cm^{-1}.

HRMS (ESI): *m/z* calcd for $C_{13}H_{14}N_2O+H^+$: 215.1179 $[M+H]^+$; found: 215.1179.

HR-MS (ESI): $C_{13}H_{14}N_2OH^+$ calculated 215.1179, found 215.1179.

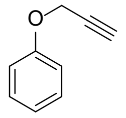

(prop-2-yn-1-yloxy)benzene (1c)[3]

Phenol (500 mg, 5.31 mmol), propargyl bromide (790 mg, 5.31 mmol) and potassium carbonate (734 mg, 5.31 mmol) were dissolved in acetone and stirred under reflux overnight. The solution was filtered, and the solvent evaporated under vacuum. The crude product was dissolved in diethyl ether and washed with 5% wt aqueous solution of NaOH (20 mL), brine (20 mL) and then dried over anhydrous Na_2SO_4. The solvent was evaporated under vacuum, and the desired compound was obtained as a yellow liquid without further purification (426 mg, 61%).

^{1}H NMR (500 MHz, $CDCl_3$) δ 7.35 – 7.27 (m, 2H), 7.04 – 6.95 (m, 3H), 4.70 (d, *J* = 2.4 Hz, 2H), 2.52 (t, *J* = 2.4 Hz, 1H).

^{13}C NMR (126 MHz, $CDCl_3$) δ 157.6, 129.5, 121.6, 114.9, 78.6, 75.4, 55.8.

IR (ν_{max}): 3291 (sp C-H), 2080 (C≡C), 1600 (C=C) cm^{-1}.

NMR spectra in agreement with literature values.[4]

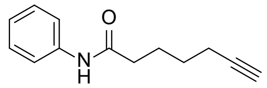

***N*-phenylhept-6-ynamide (1d)**[5]

Hept-6-ynoic acid (200 mg, 1.58 mmol), aniline (162 mg, 1.74 mmol) and 4-dimethylaminopyridine (20 mg, 0.16 mmol) were dissolved in dichloromethane (10 mL) and stirred at room temperature. DCC (326 mg, 1.58 mmol) was dissolved in dichloromethane (10 mL) and this solution was slowly added to the stirred solution. After 4 h the suspension was cooled in an ice bath and filtered. The filtrate was collected and washed subsequently with a 5 % wt aqueous solution of HCl (2 × 20 mL), a saturated solution of $NaHCO_3$ (20 mL), brine (20 mL) and dried over anhydrous Na_2SO_4. The solvent was removed under vacuum and the residue purified by column chromatography (0 – 20% ethyl acetate in petroleum ether) to give the desired compound as a white solid (292 mg, 92%).

^{1}H NMR (500 MHz, $CDCl_3$) δ 7.51 (d, *J* = 7.9 Hz, 2H), 7.36 – 7.27 (m, 3H), 7.10 (t, *J* = 7.4 Hz, 1H), 2.39 (t, *J* = 7.5 Hz, 2H), 2.28 – 2.20 (m, 2H), 1.97 (t, *J* = 2.6 Hz, 1H), 1.91 – 1.80 (m, 2H), 1.67 – 1.57 (m, 2H).

^{13}C NMR (126 MHz, $CDCl_3$) δ 170.9, 137.9, 129.0, 124.3, 119.8, 84.0, 68.7, 37.1, 27.8, 24.6, 18.2.

IR (ν_{max}**)**: 3271 (sp C-H), 2951 (C-H), 2163 (C≡C), 1657 (C=O) cm^{-1}.

NMR spectra in agreement with literature values.[6]

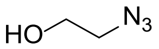

2-azidoethan-1-ol (4b)

Bromoethanol (6.2 g, 50 mmol) was dissolved in water (20 mL) and sodium azide (3.9 g, 60 mmol) was added (*ATTENTION:* azides are explosive). The reaction was heated to 50 °C for 21 h and then cooled to room temperature. Sodium chloride was added to saturate the aqueous solution which was then extracted with diethyl ether (3 × 50 mL). The combined organic phases were washed with brine (50 mL) and the solvent was removed under a stream of air to give the desired compound as a colourless oil (3.1 g, 72%).

^{1}H NMR (500 MHz, $CDCl_3$) δ 3.82 – 3.75 (m, 2H), 3.45 (t, *J* = 5.0 Hz, 2H), 1.74 (t, *J* = 5.9 Hz, 1H).

^{13}C NMR (126 MHz, $CDCl_3$) δ 61.6, 53.6.

IR (**ν_{max}**): 3361 (broad, O-H), 2097 (N_3) cm^{-1}.

NMR spectra in agreement with literature values.[7]

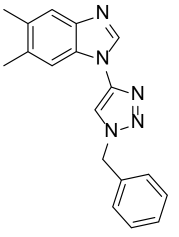

1-(1-benzyl-1*H*-1,2,3-triazol-4-yl)-5,6-dimethyl-1*H*-benzo[*d*]imidazole (5a)

1-ethynyl-5,6-dimethyl-1H-benzo[d]imidazole (200 mg, 1.17 mmol), $Cu(OAc)_2$•H_2O (24 mg, 0.12 mmol) and benzyl azide (160 mg, 1.20 mmol) were dissolved in methanol (2 mL). The reaction was stirred at room temperature for 24 h, then ethyl acetate was added. The mixture was washed with a saturated solution of EDTA (pH 3, 2 × 30 mL), brine (30 mL) and then dried over anhydrous Na_2SO_4. The solvents were removed under vacuum and the residue was purified by column chromatography (80% ethyl acetate in petroleum ether) to give the desired compound as a light-yellow solid (230 mg, 64%).

^{1}H NMR (500 MHz, DMSO) δ 8.80 (s, 1H), 8.52 (s, 1H), 7.66 (s, 1H), 7.53 (s, 1H), 7.45 – 7.40 (m, 4H), 7.40 – 7.34 (m, 1H), 5.73 (s, 2H), 2.36 (s, 3H), 2.33 (s, 3H).

^{13}C NMR (126 MHz, DMSO) δ 142.1, 141.9, 141.1, 135.5, 132.7, 131.3, 130.7, 128.9, 128.3, 128.0, 119.9, 115.7, 111.7, 53.8, 20.1, 19.8.

IR (ν_{max}): 3090 (ar C-H), 2958 (C-H), 1584 (C=C), 1495 (C=C), 1459 (C=C) cm^{-1}.

HRMS (ESI): *m*/*z* calcd for $C_{18}H_{17}N_5+H^+$: 304.1557 $[M+H]^+$; found: 304.1558.

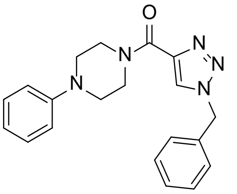

(1-benzyl-1*H*-1,2,3-triazol-4-yl)(4-phenylpiperazin-1-yl)methanone (5b)

1-(4-phenylpiperazin-1-yl)prop-2-yn-1-one (20 mg, 93 µmol), $Cu(OAc)_2$•H_2O (1.8 mg, 9 µmol) and benzyl azide (13.6 mg, 102 µmol) were dissolved in methanol (2 mL). The reaction was stirred at room temperature for 24 h, then ethyl acetate (20 mL) was added. The mixture was washed with a saturated solution of EDTA (pH 3, 2 × 20 mL), brine (20 mL) and then dried over anhydrous Na_2SO_4. The solvent

was removed under vacuum and the residue was purified by column chromatography (50% ethyl acetate in petroleum ether with 1% triethylamine) to give the desired compound as a beige solid (18 mg, 56%).

^{1}H NMR (500 MHz, $CDCl_3$) δ 8.03 (s, 1H), 7.41 – 7.33 (m, 3H), 7.32 – 7.23 (m, 4H), 6.97 (d, *J* = 8.0 Hz, 2H), 6.90 (t, *J* = 7.3 Hz, 1H), 5.53 (s, 2H), 4.51 – 4.47 (m, 2H), 3.95 – 3.89 (m, 2H), 3.29 – 3.22 (m, 4H).

^{13}C NMR (126 MHz, $CDCl_3$) δ 159.7, 150.7, 144.6, 133.8, 129.3, 129.3, 129.1, 128.4, 128.4, 120.7, 116.8, 54.4, 50.2, 49.7, 46.4, 42.6.

IR (ν_{max}): 3120 (ar C-H), 2802 (C-H), 1603 (C=C) cm^{-1}.

HRMS (ESI): *m/z* calcd for $C_{20}H_{21}N_5O+H^+$: 348.1819 [*M*+H]$^+$; found: 348.1822.

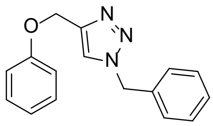

1-benzyl-4-(phenoxymethyl)-1*H*-1,2,3-triazole (5c)

(prop-2-yn-1-yloxy)benzene (20 mg, 151 μmol), $Cu(OAc)_2$•H_2O (1.6 mg, 8 μmol), benzyl azide (22 mg, 166 μmol) and THPTA (8 mg, 15 μmol) were dissolved in methanol (2 mL). Sodium ascorbate (3 mg, 15 μmol) was dissolved in the minimum amount of water and added dropwise to the reaction which was stirred at room temperature for 24 h, then dichloromethane (20 mL) was added. The mixture was washed with a saturated solution of EDTA (pH 3, 2 × 20 mL), followed by brine (20 mL) and then dried over anhydrous Na_2SO_4. The solvent was removed under vacuum and the residue purified by column chromatography (30% ethyl acetate in petroleum ether) to give the desired compound as a white solid (25 mg, 62%).

^{1}H NMR (500 MHz, $CDCl_3$) δ 7.53 (s, 1H), 7.42 – 7.32 (m, 3H), 7.32 – 7.24 (m, 4H), 7.00 – 6.93 (m, 3H), 5.53 (s, 2H), 5.19 (s, 2H).

^{13}C NMR (126 MHz, $CDCl_3$) δ 158.2, 144.7, 134.4, 129.6, 129.2, 128.9, 128.2, 122.6, 121.3, 114.8, 62.1, 54.4.

IR (ν_{max}): 3133 (ar. C-H), 2924 (C-H), 1600 (C=C), 1486 (CH_2) cm^{-1}.

HRMS (ESI): *m/z* calcd for $C_{16}H_{15}N_3O+H^+$: 266.1288 [*M*+H]$^+$; found: 266.1291.

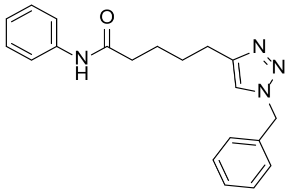

5-(1-benzyl-1*H*-1,2,3-triazol-4-yl)-*N*-phenylpentanamide (5d)

N-phenylhept-6-ynamide (20 mg, 99 μmol), $Cu(OAc)_2$•H_2O (1 mg, 5 μmol), benzyl azide (15 mg, 110 μmol) and THPTA (5 mg, 10 μmol) were dissolved in methanol (2 mL). Sodium ascorbate (2 mg, 10 μmol) was dissolved in the minimum amount of water and the solution was added dropwise to the reaction which was stirred at room temperature for 24 h, then dichloromethane (20 mL) was added. The mixture was washed with saturated solution of EDTA (pH 3, 2 × 20 mL), brine (20 mL) and then dried over anhydrous Na_2SO_4. The solvent was removed under vacuum and the

residue was purified by column chromatography (80% ethyl acetate in petroleum ether) to give the desired compound as a white solid (31 mg, 94%).

^{1}H NMR (500 MHz, $CDCl_3$) δ 7.84 (s, 1H), 7.58 (d, *J* = 8.0 Hz, 2H), 7.40 – 7.34 (m, 2H), 7.30 (t, *J* = 7.9 Hz, 2H), 7.29 – 7.23 (m, 4H), 7.08 (t, *J* = 7.4 Hz, 1H), 5.49 (s, 2H), 2.77 (app. t, *J* = 6.6 Hz, 2H), 2.41 (app. t, *J* = 6.9 Hz, 2H), 1.80 – 1.74 (m, 4H).

^{13}C NMR (126 MHz, $CDCl_3$) δ 171.3, 147.7, 138.3, 134.4, 129.2, 129.0, 128.9, 128.2, 124.0, 121.3, 119.8, 54.5, 37.0, 28.4, 24.8, 24.7.

IR (ν_{max}): 3297 (N-H), 3112 (ar. C-H), 3066 (C-H), 2936 (C-H), 1654 (C=C), 1603 (C=O) cm^{-1}.

HRMS (ESI): *m/z* calcd for $C_{20}H_{22}N_4O+H^+$: 335.1866 [*M*+H]$^+$; found: 335.1869.

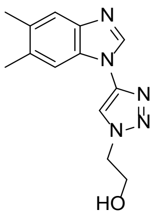

2-(4-(5,6-dimethyl-1*H*-benzo[*d*]imidazol-1-yl)-1*H*-imidazol-1-yl)ethan-1-ol (5e)

1-ethynyl-5,6-dimethyl-1H-benzo[d]imidazole (20 mg, 0.12 mmol) was dissolved in MeOH (2 mL). Then $Cu(OAc)_2$•H_2O (2 mg, 0.01 mmol) was added, followed by 2-azidoethan-1-ol (12 mg, 0.14 mmol) and the reaction was stirred at room temperature overnight. A saturated solution of EDTA (pH 3, 20 mL) and ethyl acetate (20 mL) were added, and the phases were separated. The aqueous phase was extracted with ethyl acetate (20 mL) and then the combined organic layers were washed with brine (30 mL) and dried over anhydrous Na_2SO_4. The solvents were removed under vacuum and the residue was purified by column chromatography (3 – 10% MeOH in DCM) to give the desired compound as a white solid (12 mg, 40%).

^{1}H NMR (500 MHz, DMSO) δ 8.69 (s, 1H), 8.52 (s, 1H), 7.66 (s, 1H), 7.54 (s, 1H), 5.13 (t, *J* = 5.4 Hz, 1H), 4.52 (t, *J* = 5.4 Hz, 2H), 3.88 (q, *J* = 5.4 Hz, 2H), 2.37 (s, 3H), 2.35 (s, 3H).

^{13}C NMR (126 MHz, DMSO) δ 142.4, 142.1, 141.6, 133.1, 131.8, 131.2, 120.4, 116.6, 112.2, 60.2, 53.7, 20.6, 20.3.

IR (ν_{max}): 3218 (broad O-H), 3088 (ar. C-H), 2926 (C-H), 1600 (C=C) cm^{-1}.

HRMS (ESI): *m/z* calcd for $C_{13}H_{15}N_5O+H^+$: 258.1349 [*M*+H]$^+$; found: 258.1340.

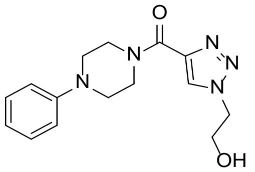

(1-(2-hydroxyethyl)-1*H*-1,2,3-triazol-4-yl)(4-phenylpiperazin-1-yl)methanone (5f)

1-(4-phenylpiperazin-1-yl)prop-2-yn-1-one (40 mg, 0.19 mmol) and 2-azidoethanol (18 mg, 0.21 mmol) were dissolved in MeOH (2 mL). Then $Cu(OAc)_2$•H_2O (2 mg, 0.01 mmol) was added and AMTC (4.2 mg, 0.02 mmol) and NaAsc (3.7 mg, 0.02 mmol) were dissolved in the minimum amount of water and then added dropwise. The reaction was stirred at room temperature for 2 hours and then a saturated solution of EDTA (pH 3, 20 mL) and ethyl acetate (20 mL) were added, and the phases were separated. The aqueous phase was extracted with ethyl acetate (20 mL) and then the combined organic

layers were washed with brine (30 mL) and dried over anhydrous Na_2SO_4. The solvents were removed under vacuum and the residue was purified by column chromatography (60% ethanol/ethyl acetate (1:3) in petroleum ether) to give the desired compound as a brown solid (38 mg, 67%).

^{1}H NMR (500 MHz, $CDCl_3$) δ 8.27 (s, 1H), 7.32 – 7.25 (m, 2H), 6.95 (d, *J* = 8.2 Hz, 2H), 6.90 (t, *J* = 7.3 Hz, 1H), 4.53 (app. t, *J* = 4.9 Hz, 2H), 4.45 (app. t, *J* = 5.1 Hz, 2H), 4.06 (app. t, *J* = 5.1 Hz, 2H), 3.91 (app, t, *J* = 5.2 Hz, 2H), 3.27 (app, t, *J* = 5.1 Hz, 4H).

^{13}C NMR (126 MHz, $CDCl_3$) δ 160.0, 150.9, 143.8, 129.5, 129.3, 120.5, 116.6, 60.9, 52.8, 50.0, 49.4, 46.6, 42.7.

IR (ν_{max}): 3408 (broad, O-H), 3117 (ar. C-H), 2923 (C-H), 1619 (C=O) cm^{-1}.

HRMS (ESI): *m*/*z* calcd for $C_{15}H_{19}N_5O_2+H^+$: 302.1612 [*M*+H]$^+$; found: 302.1606.

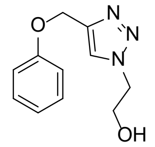

2-(4-(phenoxymethyl)-1*H*-1,2,3-triazol-1-yl)ethan-1-ol (5g)

(prop-2-yn-1-yloxy)benzene (40 mg, 0.3 mmol) was dissolved in MeOH (2 mL). $Cu(OAc)_2$•H_2O (6 mg, 0.03 mmol) was added, followed by 2-azidoethan-1-ol (29 mg, 0.3 mmol) and sodium ascorbate (60 mg, 0.3 mmol), then reaction was stirred at room temperature overnight. A saturated solution of EDTA (pH 3, 20 mL) and diethyl ether (20 mL) were added, and the phases were separated. The aqueous phase was extracted with diethyl ether (20 mL) and then the combined organic layers were washed with brine (30 mL) and dried over anhydrous Na_2SO_4. The solvents were removed under vacuum and the residue was purified by column chromatography (80% ethyl acetate in petroleum ether) to give the desired product as a clear oil (13 mg, 20%).

^{1}H NMR (500 MHz, $CDCl_3$) δ 7.73 (s, 1H), 7.32 – 7.26 (m, 2H), 7.00 – 6.96 (m, 3H), 5.19 (s, 2H), 4.48 (app. t, *J* = 5.0 Hz, 2H), 4.07 (app. t, *J* = 5.1 Hz, 2H), 2.23 (br. s, 1H).

^{13}C NMR (126 MHz, $CDCl_3$) δ 158.2, 144.1, 129.6, 123.9, 121.3, 114.8, 61.9, 61.1, 52.7.

IR (ν_{max}): 3283 (O-H), 3146 (ar. C-H), 2974 (C-H), 1599 (C=C), 1495 (C=C), 1212 (C-O) cm^{-1}.

HRMS (ESI): *m*/*z* calcd for $C_{11}H_{13}N_3O_2+H^+$: 220.1081 [*M*+H]$^+$; found: 220.1076.

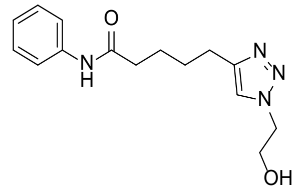

5-(1-(2-hydroxyethyl)-1*H*-1,2,3-triazol-4-yl)-*N*-phenylpentanamide (5h)

N-phenylhept-6-ynamide (40 mg, 0.2 mmol) was dissolve in MeOH (2 mL). $Cu(OAc)_2$•H_2O (4 mg, 0.02 mmol) was added, followed by 2-azidoethan-1-ol (19 mg, 0.22 mmol) and sodium ascorbate (39 mg, 0.2 mmol), then the reaction was stirred at room temperature overnight. A saturated solution of EDTA (pH 3, 20 mL) and ethyl acetate (20 mL) were added, and the phases were separated. The aqueous phase was extracted with ethyl acetate (2 × 20 mL) and then the combined organic layers were washed with brine and dried over anhydrous Na_2SO_4. The solvent was removed under

vacuum and the residue was purified by column chromatography (60% ethanol/ethyl acetate (1:3) in petroleum ether) to give the desired compound as a clear oil (24 mg, 42%).

^{1}H NMR (500 MHz, DMSO) δ 9.85 (s, 1H), 7.80 (s, 1H), 7.58 (d, *J* = 7.9 Hz, 2H), 7.27 (app. t, *J* = 7.7 Hz, 2H), 7.01 (t, *J* = 7.4 Hz, 1H), 4.98 (br. s, 1H), 4.33 (app t, *J* = 5.5 Hz, 2H), 3.75 (app. t, *J* = 5.5 Hz, 2H), 2.67 – 2.60 (m, 2H), 2.37 – 2.30 (m, 2H), 1.66 – 1.61 (m, 4H).

^{13}C NMR (126 MHz, DMSO) δ 171.6, 146.8, 139.8, 129.1, 123.4, 122.6, 119.5, 60.4, 52.4, 36.6, 29.1, 25.3, 25.2.

IR (ν_{max}): 3268 (N-H), 3063 (ar. C-H), 2933 (C-H), 1651 (C=O) cm^{-1}, O-H observed but not reported, overlaps with N-H.

HRMS (ESI): *m*/*z* calcd for $C_{15}H_{20}N_4O_2+H^+$: 289.1659 [*M*+H]$^+$; found: 289.1651.

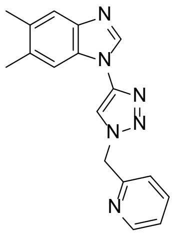

5,6-dimethyl-1-(1-(pyridin-2-ylmethyl)-1*H*-1,2,3-triazol-4-yl)-1*H*-benzo[*d*]imidazole (5i)

1-ethynyl-5,6-dimethyl-1H-benzo[d]imidazole (20 mg, 0.12 mmol) was dissolved in MeOH (2 mL). Then $Cu(OAc)_2{\bullet}H_2O$ (2 mg, 0.01 mmol) was added, followed by picolyl azide (12 mg, 0.14 mmol) and the reaction was stirred at room temperature overnight. A saturated solution of EDTA (pH 3, 20 mL) and ethyl acetate (10 mL) were added, and the phases were separated. The aqueous phase was extracted with ethyl acetate (20 mL) and then the combined organic layers were washed with brine (30 mL) and dried over anhydrous Na_2SO_4. The solvent was removed under vacuum and the residue was purified by column chromatography (5 - 10% MeOH in DCM) to give the desired product as a brown oil (26 mg, 73%).

^{1}H NMR (500 MHz, DMSO) δ 8.84 (s, 1H), 8.70 (s, 1H), 8.61 – 8.55 (m, 1H), 7.88 (td, *J* = 7.7, 1.8 Hz, 1H), 7.70 (s, 1H), 7.56 (s, 1H), 7.44 (d, *J* = 7.6 Hz, 1H), 7.40 (ddd, *J* = 7.6, 4.8, 1.1 Hz, 1H), 5.85 (s, 2H), 2.37 (s, 3H), 2.35 (s, 3H).

^{13}C NMR (126 MHz, DMSO) δ 154.4, 149.5, 141.8, 141.1, 140.6, 137.5, 133.1, 131.9, 130.5, 123.5, 122.4, 119.4, 116.7, 111.9, 55.2, 20.1, 19.8.

IR (ν_{max}): 3107 (ar. C-H), 2923 (C-H), 1587 (C=C) cm^{-1}.

HRMS (ESI): *m*/*z* calcd for $C_{17}H_{16}N_6+H^+$: 305.1509 [*M*+H]$^+$; found: 305.1508.

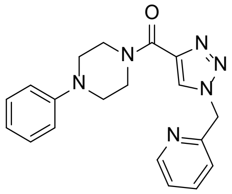

(4-phenylpiperazin-1-yl)(1-(pyridin-2-ylmethyl)-1*H*-1,2,3-triazol-4-yl)methanone (5j)

1-(4-phenylpiperazin-1-yl)prop-2-yn-1-one (40 mg, 0.19 mmol) was dissolved in MeOH (2 mL). Then $Cu(OAc)_2{\bullet}H_2O$ (4 mg, 0.02 mmol) was added, followed by picolyl azide (28 mg, 0.21 mmol) and the reaction was stirred at room temperature overnight. A saturated solution of EDTA (pH 3, 20 mL) and ethyl acetate (20 mL) were added, and the phases were separated. The aqueous phase was extracted with ethyl

acetate (20 mL) and then the combined organic layers were washed with brine (20 mL) and dried over anhydrous Na_2SO_4. The solvent was removed under vacuum and the residue was purified by column chromatography (60% ethanol/ethyl acetate (1:3) in petroleum ether) to give the desired compound as a brown solid (9 mg, 13%).

^{1}H NMR (500 MHz, $CDCl_3$) δ 8.63 – 8.58 (m, 1H), 8.26 (s, 1H), 7.70 (td, *J* = 7.7, 1.8 Hz, 1H), 7.32 – 7.21 (m, 4H), 6.96 (d, *J* = 8.1 Hz, 2H), 6.90 (t, *J* = 7.3 Hz, 1H), 5.68 (s, 2H), 4.48 (app. t, *J* = 5.0 Hz, 2H), 3.94 (app. t, *J* = 5.1 Hz, 2H), 3.27 (app. t, *J* = 5.3 Hz, 4H).

^{13}C NMR (126 MHz, $CDCl_3$) δ 159.7, 153.6, 150.9, 150.0, 144.6, 137.4, 129.3, 129.0, 123.7, 122.6, 120.6, 116.7, 55.7, 50.2, 49.6, 46.5, 42.6.

IR (ν_{max}): 3123 (ar. C-H), 2793 (C-H), 1609 (C=O) cm^{-1}.

HRMS (ESI): *m*/*z* calcd for $C_{19}H_{20}N_6O+H^+$: 349.1771 [*M*+H]$^+$; found: 349.1769.

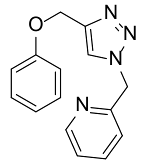

2-((4-(phenoxymethyl)-1*H*-1,2,3-triazol-1-yl)methyl)pyridine (5k)

(prop-2-yn-1-yloxy)benzene (40 mg, 0.3 mmol) was dissolved in MeOH (2 mL). $Cu(OAc)_2$•H_2O (6 mg, 0.03 mmol) was added, followed by picolyl azide (45 mg, 0.3 mmol) and sodium ascorbate (60 mg, 0.3 mmol), then reaction was stirred at room temperature overnight. A saturated solution of EDTA (pH 3, 20 mL) and diethyl ether (20 mL) were added, and the phases were separated. The aqueous phase was extracted with diethyl ether (20 mL) and then the combined organic layers were washed with brine (30 mL) and dried over anhydrous Na_2SO_4. The solvents were removed under vacuum and the residue purified by column chromatography (70% ethyl acetate in petroleum ether) to give the desired compound as a clear oil (41 mg, 51%).

^{1}H NMR (500 MHz, $CDCl_3$) δ 8.62 – 8.57 (m, 1H), 7.79 (s, 1H), 7.69 (td, *J* = 7.7, 1.8 Hz, 1H), 7.31 – 7.26 (m, 3H), 7.20 (d, *J* = 7.8 Hz, 1H), 7.00 – 6.93 (m, 3H), 5.66 (s, 2H), 5.22 (s, 2H).

^{13}C NMR (126 MHz, $CDCl_3$) δ 158.2, 154.3, 149.8, 144.7, 137.4, 129.5, 123.5, 123.3, 122.5, 121.3, 114.8, 62.1, 55.7.

IR (ν_{max}): 3132 (ar. C-H), 2926 (C-H), 1603 (C=C) cm^{-1}.

HRMS (ESI): *m*/*z* calcd for $C_{15}H_{14}N_4O+H^+$: 267.1240 [*M*+H]$^+$; found: 267.1253.

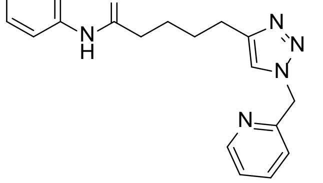

***N*-phenyl-5-(1-(pyridin-2-ylmethyl)-1*H*-1,2,3-triazol-4-yl)pentanamide (5l)**

N-phenylhept-6-ynamide (40 mg, 0.2 mmol) was dissolve in MeOH (2 mL). $Cu(OAc)_2$•H_2O (4 mg, 0.02 mmol) was added, followed by picolyl azide (29 mg, 0.22 mmol) and sodium ascorbate (39 mg, 0.2 mmol), then reaction was stirred at room temperature overnight. A saturated solution of EDTA (pH 3, 20 mL) and ethyl acetate (20 mL) were added, and the phases were separated. The aqueous phase was extracted with ethyl acetate (20 mL) and then the combined organic layers

were washed with brine and dried over anhydrous Na_2SO_4. The solvent was removed under vacuum and the residue was purified by column chromatography (70% ethanol/ethyl acetate (1:3) in petroleum ether) to give the desired compound as a clear oil which crystallized upon cooling (39 mg, 67%).

^{1}H NMR (500 MHz, DMSO) δ 9.85 (s, 1H), 8.56 – 8.51 (m, 1H), 7.92 (s, 1H), 7.80 (td, *J* = 7.7, 1.8 Hz, 1H), 7.57 (d, *J* = 7.7 Hz, 2H), 7.37 – 7.30 (m, 1H), 7.31 – 7.22 (m, 2H), 7.20 (d, *J* = 7.9 Hz, 1H), 7.01 (t, *J* = 7.4 Hz, 1H), 5.64 (s, 2H), 2.69 – 2.62 (m, 2H), 2.37 – 2.29 (m, 2H), 1.68 – 1.59 (m, 4H).

^{13}C NMR (126 MHz, DMSO) δ 171.6, 155.8, 149.8, 147.3, 139.8, 137.8, 129.1, 123.6, 123.4, 123.1, 122.5, 119.5, 54.8, 36.6, 29.1, 25.3, 25.2.

IR (ν_{max}): 3275 (N-H), 3136 (ar. C-H), 2955 (C-H), 1691 (C=O) cm^{-1}.

HRMS (ESI): *m/z* calcd for $C_{19}H_{21}N_5O+H^+$: 336.1819 $[M+H]^+$; found: 336.1812.

Synthesis of *N*-(1-ethynyl-1*H*-benzo[*d*]imidazol-6-yl)-6-((4*R*,5*S*)-5-methyl-2-oxoimidazolidin-4-yl)hexanamide (8):

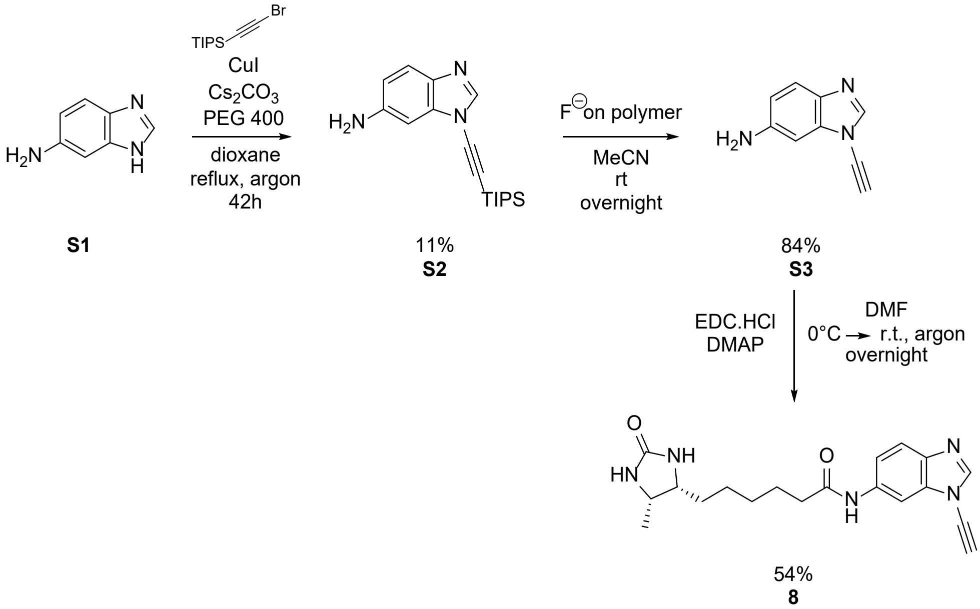

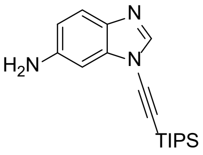

1-((triisopropylsilyl)ethynyl)-1*H*-benzo[*d*]imidazol-5-amine (S2)

1H-benzo[d]imidazol-6-amine (2.0 g, 15 mmol), caesium carbonate (5.9 g, 18 mmol), copper iodide (143 mg, 0.75 mmol) and PEG 400 (1.2 g, 3.0 mmol) were dissolved in dry dioxane (100 mL). The mixture was degassed under vacuum three times (20 min, 10 min, 5 min) and (bromoethynyl)triisopropylsilane (4.7 g, 18 mmol) was added. The reaction mixture was heated to reflux (110 °C) for 42 hours under an argon atmosphere. The reaction was allowed to reach room temperature and the diluted with ethyl acetate (100 mL). The mixture was washed with a saturated solution of EDTA (pH 3, 100 mL), brine (100 mL) and then dried over Na_2SO_4. The organic phase was concentrated

under vacuum and purified by flash column chromatography (50 – 70% ethyl acetate in petroleum ether) to give the desired compounds as yellow solids (505 mg, 11%).

^{1}H NMR (500 MHz, $CDCl_3$) δ 7.88 (s, 1H), 7.55 (d, *J* = 8.5 Hz, 1H), 6.78 (d, *J* = 2.2 Hz, 1H), 6.72 (dd, *J* = 8.5, 2.2 Hz, 1H), 3.83 (s, 2H), 1.16 (s, 21H).

^{13}C NMR (126 MHz, $CDCl_3$) δ 144.5, 141.8, 135.9, 135.1, 121.2, 113.5, 96.0, 90.5, 72.5, 18.7, 11.2.

IR (ν_{max}): 3323 (N-H), 3210 (N-H), 2943 (C-H), 2865 (C-H), 2187 (C≡C), 1498 (ar. C=C) cm $^{-1}$.

HRMS (ESI): *m*/*z* calcd for $C_{18}H_{27}N_3Si+H^+$: 314.2047 [*M*+H]$^+$; found: 314.2047.

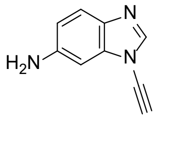

1-ethynyl-1*H*-benzo[*d*]imidazol-6-amine (S3)

1-((triisopropylsilyl)ethynyl)-1H-benzo[d]imidazol-6-amine (359 mg, 1.15 mmol) was dissolved in acetonitrile (6 mL) and fluoride on polymer support 2 – 3 mmol/g loading (383 mg, 1.15 mmol, Sigma catalogue number: 387789-10G) was added. The reaction was stirred at room temperature overnight, then filtered and the solvent removed under vacuum. The residue was purified by flash column chromatography (0 – 60% in ethyl acetate in petroleum ether) and the desired product was obtained as a light brown solid (152 mg, 84%).

^{1}H NMR (500 MHz, $CDCl_3$) δ 7.87 (s, 1H), 7.55 (d, *J* = 8.6 Hz, 1H), 6.83 (d, *J* = 2.2 Hz, 1H), 6.72 (dd, *J* = 8.6, 2.2 Hz, 1H), 3.84 (s, 2H), 3.24 (s, 1H).

^{13}C NMR (126 MHz, $CDCl_3$) δ 144.9, 141.7, 135.9, 135.2, 121.5, 113.7, 96.0, 70.8, 61.9.

IR (ν_{max}): 3409 (N-H), 3299 (N-H), 3180 (ar. C-H) 3116 (sp C-H), 2146 (C≡C), 1497 (ar. C=C) cm $^{-1}$.

HRMS (ESI): *m*/*z* calcd for $C_9H_7N_3+H^+$: 158.0713 [*M*+H]$^+$; found: 158.0712.

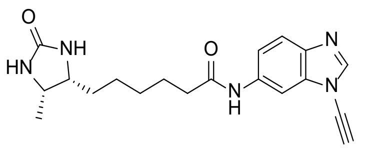

***N*-(1-ethynyl-1*H*-benzo[*d*]imidazol-6-yl)-6-((4*R*,5*S*)-5-methyl-2-oxoimidazolidin-4-yl)hexanamide (8)**

Desthiobiotin (40 mg, 0.18 mmol), DMAP (5 mg, 0.04 mmol) and then EDC.HCl (40 mg, 0.21 mmol) were dissolved in DMF (3 ml) and stirred for 30 minutes under argon atmosphere and cooled to 0 °C, then 1-ethynyl-1*H*-benzo[*d*]imidazol-6-amine (33 mg, 0.21 mmol) was added, and reaction stirred overnight. Dichloromethane (20 mL) was added, the organic phase was extracted with a saturated solution of $NaHCO_3$ (20 mL) and water (20 mL), then dried over Na_2SO_4. The solvent was removed under vacuum and the residue purified by column chromatography (7% methanol/ 1% TEA in dichloromethane) to give the desired compound as a white solid (36 mg, 54%).

^{1}H NMR (400 MHz, DMSO) δ 10.12 (s, 1H), 8.54 (s, 1H), 8.26 (d, *J* = 2.0 Hz, 1H), 7.67 (d, *J* = 8.7 Hz, 1H), 7.33 (dd, *J* = 8.7, 2.0 Hz, 1H), 6.30 (s, 1H), 6.11 (s, 1H), 4.73 (s, 1H), 3.67 – 3.55

(m, 1H), 3.54 – 3.45 (m, 1H), 2.34 (t, *J* = 7.4 Hz, 2H), 1.67 – 1.55 (m, 2H), 1.46 – 1.19 (m, 6H), 0.96 (d, *J* = 6.4 Hz, 3H).

^{13}C NMR (101 MHz, DMSO) δ 171.4, 162.8, 144.2, 137.0, 136.8, 134.3, 120.3, 116.0, 100.5, 70.2, 64.9, 55.0, 50.2, 36.4, 29.5, 28.7, 25.6, 25.0, 15.5.

IR (ν_{max}): 3256 (N-H), 3133 (ar. C-H), 2930 (C-H), 1707 (C=O), 1595 (C=O) cm^{-1}.

HRMS (ESI): *m*/*z* calcd for $C_{19}H_{23}N_5O_2+H^+$: 354.1925 [*M*+H]$^+$; found: 354.1935.

Synthesis of 2-cyanoethyl (6-(1-((triisopropylsilyl)ethynyl)-1*H*-benzo[*d*]imidazol-6-yl)hexyl) diisopropylphosphoramid (S9):

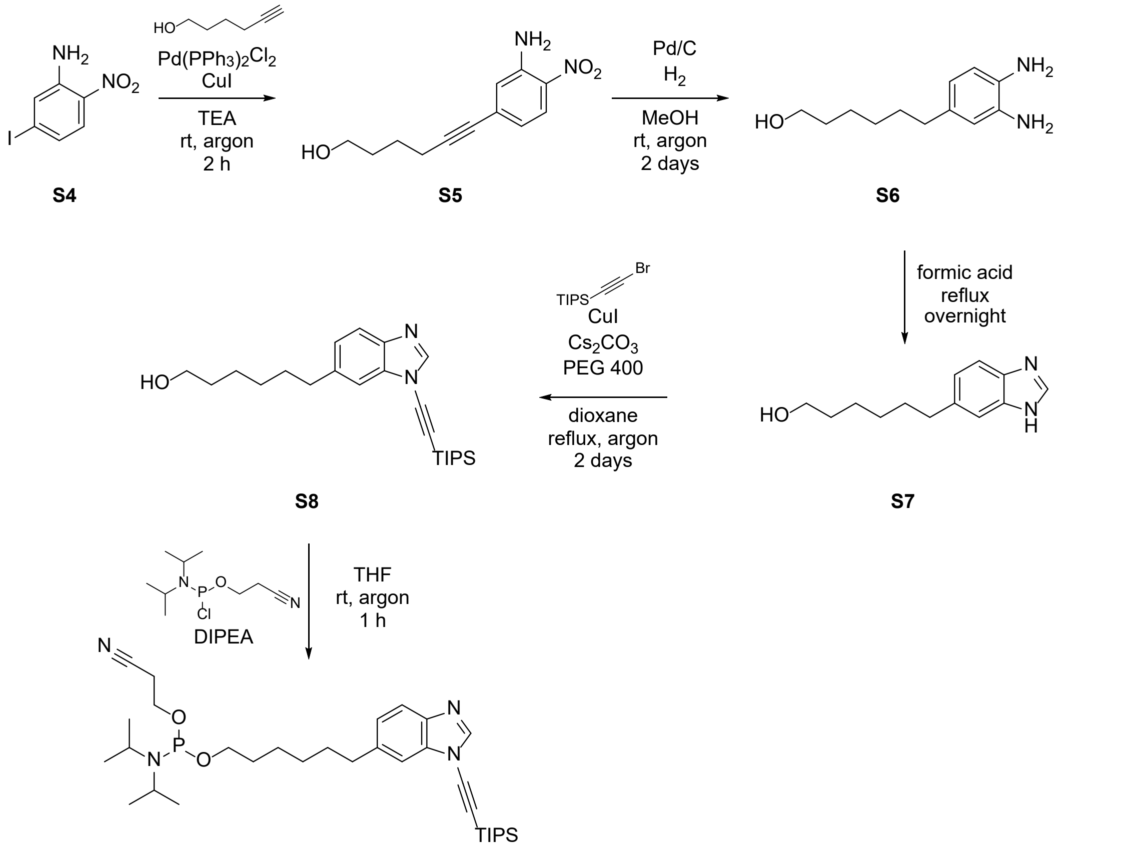

S9

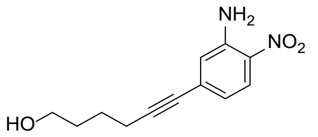

6-(3-amino-4-nitrophenyl)hex-5-yn-1-ol (S5)

4-Iodo-2-nitroaniline (11 g, 42 mmol), $Pd(PPh_3)_2Cl_2$ (3.2 g, 4.6 mmol) and CuI (634 mg, 3.3 mmol) were added to a flask which was put under vacuum and refilled with argon. Then triethylamine (200 mL) and hex-5-yn-1-ol (4.5 g, 46 mmol) were added and the reaction was stirred under argon atmosphere for 2 h. Diethyl ether (100 mL) was added and the suspension was filtered through celite and eluted with more diethyl ether (100 mL). The filtrate was concentrated under vacuum and washed with an aqueous solution of 5% wt HCl (70 mL), a solution of saturated $NaHCO_3$ (2 × 70 mL), brine

and then dried over Na_2SO_4. The solvents were removed under vacuum to give a brown solid, which was dissolved in ethyl acetate (50 mL) and heated to 40 °C. Hexane (200 mL) was added dropwise whereupon orange solid precipitated. The solid was filtered off, washed with hexane, and dried under vacuum the give the desired product as an orange solid (9.4 g, 97 %).

^{1}H NMR (500 MHz, $CDCl_3$) δ 8.16 (d, *J* = 2.0 Hz, 1H), 7.34 (dd, *J* = 8.6, 2.0 Hz, 1H), 6.72 (d, *J* = 8.6 Hz, 1H), 6.15 (br. s, 2H), 3.74 – 3.64 (m, 2H), 2.43 (t, *J* = 6.8 Hz, 2H), 1.79 – 1.57 (m, 4H), 1.40 (br. s, 1H).

^{13}C NMR (126 MHz, $CDCl_3$) δ 144.0, 138.5, 131.8, 129.3, 118.8, 112.9, 89.2, 79.2, 62.6, 32.0, 25.1, 19.3.

IR (ν_{max}): 3533 (N-H), 3444 (N-H), 3170 (ar. C-H), 2941 (C-H), 1636 (NO_2) cm^{-1}.

HRMS (ESI): *m*/*z* calcd for $C_{12}H_{14}N_2O_3+H^+$: 235.1077 [*M*+H]$^+$; found: 235.1091.

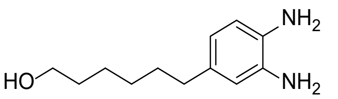

6-(3,4-diaminophenyl)hexan-1-ol (S6)

6-(3-amino-4-nitrophenyl)hex-5-yn-1-ol (9.4 g, 402 mmol) was added to a two-neck 1 L round bottom flask, which was purged with argon three times. Palladium on carbon was added (10 spatula tips) and the flask was put under vacuum and refilled with argon. Dry methanol (400 mL) was added and the solution sparged with 1.5 balloons of hydrogen. The reaction was then kept under a hydrogen atmosphere overnight and stirred at room temperature. TLC analysis indicated incomplete conversion, therefore more catalyst (10 spatula tips) was added, the reaction sparged with hydrogen and kept under a hydrogen atmosphere for another night. Then the reaction was filtered through celite and eluted with MeOH. The solvent was removed under vacuum to obtain the desired product as a purple solid (7.8 g, 93%).

^{1}H NMR (400 MHz, $CDCl_3$) δ 6.62 (d, *J* = 7.4 Hz, 1H), 6.54 – 6.50 (m, 2H), 3.63 (t, *J* = 6.6 Hz, 2H), 3.27 (br. s, 4H), 2.52 – 2.42 (m, 2H), 1.63 – 1.50 (m, 4H), 1.44 – 1.23 (m, 4H).

^{13}C NMR (101 MHz, $CDCl_3$) δ 135.2, 135.0, 132.3, 120.0, 117.1, 116.9, 63.2, 35.3, 32.9, 31.8, 29.2, 25.8.

IR (ν_{max}): 3375 (N-H), 3290 (broad, O-H), 2925 (C-H), 2852 (C-H), 1519 (C=C) cm^{-1}.

HRMS (ESI): *m*/*z* calcd for $C_{12}H_{20}N_2O+H^+$: 209.1648 [*M*+H]$^+$; found: 209.1649.

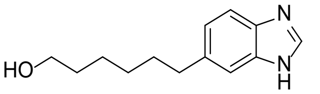

6-(1*H*-benzo[*d*]imidazol-6-yl)hexan-1-ol (S7)

6-(3,4-diaminophenyl)hexan-1-ol (370 mg, 1.8 mmol) was dissolved in formic acid (5 mL) and heated to reflux overnight. The solvent was removed under vacuum and the residue was dissolved in ethyl acetate (30 mL). The organic phase was washed with an aqueous solution of 5% wt NaOH and brine (30 mL). The solvent was removed under

vacuum and the residue purified by column chromatography (5 – 15% MeOH in DCM) to give the desired compound as a brown oil (157 mg, 40%).

^{1}H NMR (400 MHz, MeOD) δ 8.12 (br. s, 1H), 7.52 (br. s, 1H), 7.41 (br. s, 1H), 7.07 (d, *J* = 8.0 Hz, 1H), 3.52 (t, *J* = 6.6 Hz, 2H), 2.69 (t, *J* = 7.6 Hz, 2H), 1.70 – 1.58 (m, 2H), 1.56 – 1.43 (m, 2H), 1.43 – 1.27 (m, 4H).

^{13}C NMR (101 MHz, MeOD) δ 138.9, 124.8, 116.1, 115.0, 62.9, 49.6, 49.4, 48.4, 37.0, 33.6, 33.2, 30.1, 26.8.

IR (ν_{max}): 3195 (broad, O-H), 2925 (C-H), 2824 (C-H), 1603 (C=C) cm^{-1}.

HRMS (ESI): *m*/*z* calcd for $C_{13}H_{18}N_2O+H^+$: 219.1492 [*M*+H]$^+$; found: 219.1502.

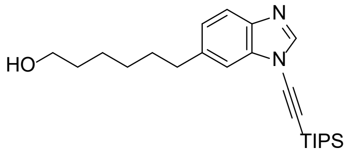

6-(1-((triisopropylsilyl)ethynyl)-1*H*-benzo[*d*]imidazol-6-yl)hexan-1-ol (S8)

6-(1H-benzo[d]imidazol-6-yl)hexan-1-ol (1.2 g, 5.4 mmol), CuI (51 mg, 0.27 mmol), caesium carbonate (2.1 g, 6.4 mmol) and PEG400 (214 mg, 0.54 mmol) were added to a flask and put under vacuum. Dry dioxane (50 mL) was added under argon atmosphere and the reaction degassed under vacuum (3 × 5 min). (Bromoethynyl)triisopropylsilane (2.2 g, 8.6 mmol) was added and the reaction heated to reflux for 2 days. The solvent was removed under vacuum and ethyl acetate (70 mL) and a saturated solution of EDTA (pH 3, 60 mL) were added and the phases were separated. The organic phase was washed with water (70 mL) and the combined aqueous phases extracted with ethyl acetate (70 mL). The organic phases were combined, washed with brine (70 mL) and then dried over Na_2SO_4. The solvent was removed under vacuum to give a brown oil, which was purified by column chromatography (10 – 50% ethyl acetate in hexane) to give the desired compound as a clear oil (383 mg, 18%).

^{1}H NMR (500 MHz, $CDCl_3$) δ 8.02 (s, 1H), 7.69 (d, *J* = 8.2 Hz, 1H), 7.32 (d, *J* = 1.5 Hz, 1H), 7.18 (dd, *J* = 8.3, 1.5 Hz, 1H), 3.64 (t, *J* = 6.6 Hz, 2H), 2.78 (t, *J* = 7.7 Hz, 2H), 1.75 – 1.65 (m, 2H), 1.63 – 1.53 (m, 2H), 1.47 – 1.38 (m, 4H), 1.24 – 1.09 (m, 21H).

^{13}C NMR (126 MHz, $CDCl_3$) δ 143.3, 140.2, 140.1, 134.9, 125.1, 120.4, 110.3, 90.5, 73.0, 63.1, 36.2, 32.9, 31.8, 29.1, 25.8, 18.8, 11.4.

IR (ν_{max}): 3304 (broad, O-H), 2927 (C-H), 2803 (C-H), 2184 (C≡C), 1500 (C=C) cm^{-1}.

HRMS (ESI): *m*/*z* calcd for $C_{24}H_{38}N_2OSi+H^+$: 399.2826 [*M*+H]$^+$; found: 399.2824.

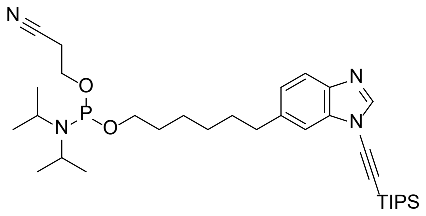

2-cyanoethyl (6-(1-((triisopropylsilyl)ethynyl)-1*H*-benzo[*d*]imidazol-6-yl)hexyl) diisopropylphosphoramidite (S9)

6-(1-((triisopropylsilyl)ethynyl)-1H-benzo[d]imidazol-6-yl)hexan-1-ol (200 mg, 0.5 mmol) was dissolved in dry THF

(4 mL) under argon in a vacuum-dried flask. Dry DIPEA (583 mg, 4.5 mmol) was added, followed by 2-cyanoethyl *N,N*-diisopropylchlorophosphoramidite (356 mg, 1.5 mmol) and the reaction was stirred at room temperature for 1 h. The solvent was removed under vacuum and the residue purified by column chromatography (5 – 20% ethyl acetate in hexane with 1% TEA) to give the desired compound as a colourless oil (208 mg, 71%).

^{1}H NMR (500 MHz, $CDCl_3$) δ 8.01 (s, 1H), 7.69 (d, *J* = 8.2 Hz, 1H), 7.32 (d, *J* = 1.5 Hz, 1H), 7.17 (dd, *J* = 8.2, 1.5 Hz, 1H), 3.89 – 3.75 (m, 2H), 3.69 – 3.52 (m, 4H), 2.77 (t, *J* = 7.6 Hz, 2H), 2.63 (t, *J* = 6.5 Hz, 3H), 1.75 – 1.56 (m, 5H), 1.47 – 1.35 (m, 4H), 1.26 – 1.12 (m, 32H).

^{13}C NMR (126 MHz, $CDCl_3$) δ 143.4, 140.2, 134.9, 125.0, 120.5, 117.8, 110.3, 90.5, 72.9, 63.8 (d, *J* = 17.2 Hz), 58.4 (d, *J* = 18.8 Hz), 43.2 (d, *J* = 12.1 Hz), 36.3, 31.8, 31.3 (d, *J* = 7.3 Hz), 29.1, 26.0, 24.8 (d, *J* = 7.3 Hz), 24.7 (d, *J* = 7.1 Hz), 20.5 (d, *J* = 6.9 Hz), 18.8, 11.4. Not phosphorous decoupled.

^{31}P NMR (162 MHz, $CDCl_3$) δ 147.3.

HRMS (ESI): *m*/*z* calcd for $C_{33}H_{56}N_4O_2PSi+H^+$: 599.3905 [*M*+H]$^+$; found: 599.3898.

1.2 General Protocol Peptide Synthesis

Solid-phase synthesis was performed using ChemMatrix® Rink amide resin (0.52 mmol/g) using a Protein Technologies Tribute automated synthesizer. Synthesis was performed using the standard Fmoc/tBu protected amino acids and were obtained from Merck without further purification.

The general experimental protocol for automated synthesis was as follows:

The resin was swollen for 10 min in DCM (2 mL). Fmoc deprotection was achieved using 20% piperidine in DMF (3 × 5 min, 2 mL). Each amino acid (3.0 equiv) was activated with HATU (3.0 equiv) and DIPEA (0.5 M in DMF, 5 mL) for 5 min. The solution was then added to the resin and shaken for 1 h, then washed with further DMF (2 mL). Deprotection, coupling, and washing (DMF, 3 × 5 min, 2 mL) procedures were repeated until the final amino acid had been coupled to the peptide chain. The peptide was then cleaved from the resin (see individual peptide sections for conditions), precipitated by addition of cold MTBE, and centrifuged at 4000 rpm for 5 min. The pellet was resuspended in cold MTBE, sonicated, and collected by centrifugation; this was repeated a total of three times. Peptides were then purified by RP-HPLC using a Dionex UltiMate 3000 HPLC equipped with a C18 column (Phenomenex Kinetex, 150 × 21.2 mm, 5 µm, 15 mL/min, A = H_2O (0.1% TFA), B = MeCN (0.1% TFA)). For purification HPLC method see individual peptide sections.

HPLC Method for Purity Analysis of Peptides

Column Specifications: Phenomenex Aeris Widepore C18, 250 × 4.6 mm, 3.6 μm

Column Temperature: 40 °C

Mobile Phase A: 0.1% v/v TFA in water

Mobile Phase B: 0.1% v/v TFA in MeCN

Flow rate: 1 mL/min

Injection Volume: 10 μL

**Time** (min)	**B%**
0 – 35	5 – 95
35 – 40	95
40 – 40.5	95 – 5
40.5 - 50	5

Penetratin-Az (6)

Synthesis was performed using the general procedure given previously (100 mg resin, 50.0 μmol). After the coupling the resin was split in half and one portion deprotected (2 × 5 min 20% piperidine in DMF, 3 mL), washed (2 × DCM, 2 × DMF, 5 mL each) and then cleaved (90% TFA, 2.5% H_2O, 2.5% TIPS, 2.5% DTT, 2.5% Phenol, v/v/v/w/w, 3 mL total) for 4 h. The resin was filtered off and the peptide was precipitated with MTBE. The residue was purified by reverse phase semi-preparative chromatography. The relevant fractions were collected then pooled and the solvent removed by lyophilisation to give the target peptide as a colourless, amorphous solid.

HPLC purity (Rt = 11.36 min): 98% (254 nm), 97% (210 nm).

HRMS (ESI): *m/z* calcd for $C_{112}H_{182}N_{40}O_{21}S+4H^+$: 614.8604 $[M+4H]^{4+}$; found: 614.8603.

Gradient for HPLC purification (A = H_2O (0.1% TFA), B = MeCN (0.1% TFA)):

**Time** (min)	**B%**
0 - 16	5 – 40
16 - 17	40 – 95
17 - 21	95
21 - 22	95 - 5
22 - 25	5

TP2-Az (7)

Synthesis was performed using the general procedure (100 mg resin, 50.0 μmol). After the coupling the resin was split in half and one portion deprotected (2 × 5 min 20% piperidine in DMF), washed (2 × DCM, 2 × DMF, 5 mL each) and then cleaved (90% TFA, 5% H_2O, 2.5% TIS, 2.5% Phenol, v/v/v/w, 2 mL total) for 2 h. The resin was filtered off and the peptide precipitated with MTBE. The residue was purified by reverse phase semi-preparative

chromatography. The relevant fractions were collected then pooled and the solvent removed by lyophilisation to give the target peptide as a colourless, amorphous solid.

HPLC purity (Rt = 15.59 min): 95% (210 nm)

HRMS (ESI): *m*/*z* calcd for $C_{78}H_{128}N_{24}O_{15}$+3H$^+$: 548.0070 [*M*+3H]$^{3+}$; found: 548.0066.

Gradient for HPLC purification (A = H_2O (0.1% TFA), B = MeCN (0.1% TFA)):

**Time** (min)	**B%**
0 – 11	5 – 50
11 – 12	50 – 95
12 – 15	95
15 – 16	95 – 5
16 - 19	5

Pico-TP2-Az (12)

The second half of the TP2-Az resin was deprotected (2 x 5 min 20% piperidine in DMF) and washed (2 × DCM, 2 × DMF). 4-((2-(azidomethyl)pyridin-4-yl)amino)-4-oxobutanoic acid (19 mg, 75 µmol) was preincubated with DIC (10 mg, 75 µmol) and OxymaPure® (11 mg, 75 µmol) for 1 h. The mixture was added to the resin and heated in the microwave to 75 °C for 1 h. The resin was washed (5 × DMF, 5 × DCM, 5 mL each) and then cleaved (90% TFA, 5% H_2O, 2.5% TIS, 2.5% Phenol v/v/v/w, 2 mL) for 2.5 h. The resin was filtered off and the peptide precipitated with MTBE. The residue was purified by reverse phase semi-preparative chromatography. The relevant fractions were collected then pooled and the solvent removed by lyophilisation to give the target peptide as a colourless, amorphous solid.

HPLC purity (Rt = 19.47 min): 100% (254 nm), 98% (210 nm).

HRMS (ESI): *m*/*z* calcd for $C_{88}H_{137}N_{29}O_{17}$+3H$^+$: 625.0322 [*M*+3H]$^{3+}$; found: 625.0322.

Gradient for HPLC purification (A = H_2O (0.1% TFA), B = MeCN (0.1% TFA)):

**Time** (min)	**B%**
0 – 11	20 – 60
11 – 12	60 – 95
12 – 15	95
15 – 16	95 – 20
16 - 19	20

1.3 General Protocol for Oligonucleotide Synthesis

Compound number	Modification	Sequence 5'-3'	Calcd Mass [M+H]+	MALDI
**S10** ODN	Ynamine (Yn)	Yn-CGC GAT ATC GCG	3949.75	3949.94
**S12** ODN	Triazole (Tz)	Tz-CGC GAT ATC GCG	4212.83	4211.82
**16** ODN	Ynamine and DBCO	Yn-CGC GAT(DBCO) ATC GCG	4418.99	4419.73
**17** ODN	Mono-Adduct Triazole	Yn-CGC GAT(DBCO) ATC GCG	Not isolated	NA
**19** ODN	Bis-Adduct Triazole	Yn(Tr)-CGC GAT(DBCO(Tr)) ATC GCG	5304.36	5304.41

ODN S10 was synthesized according to standard solid-phase oligonucleotide-synthesis protocols on a 1 μM scale using an ABI 392 synthesizer. Coupling efficiency was monitored after removal of the dimethoxytrityl (DMTr) 5′-OH protecting groups. Standard phosphoramidites and controlled pore glass (CPG, 1000Å/110mm) supports loaded with standard nucleosides were purchased from LINK-LGC Biosearch Technologies.

Standard phosphoramidites (1 g bottle) were dissolved in acetonitrile to obtain a 0.1 M solution. Ynamine phosphoramidite **S9** (101 mg) was dissolved in 1.7 mL of acetonitrile to obtain a 0.1 M solution. Standard cycle 1 μM was used for the coupling procedure.

After the solid phase synthesis was finished the TIPS protecting group was manually removed on solid phase. TBAF (50 μL, 1 M in THF) was mixed with 1.95 mL of acetonitrile and this mixture was taken up in a plastic syringe. The mixture was repeatedly passed over the column containing the CPG support for 3 min. Next, the CPG support was washed with MeCN (3 × 5 mL) and dried with air.

For cleaving and deprotection 1 mL of 28% NH_4OH aqueous solution was added, and the suspension was shaken for 16 h at room temperature. (ATTENTION: Higher Temperatures led to degradation of the ynamine modifiers and need to be avoided!). The mixture was filtered, and the CPG support was then washed with water (2 × 1.5 mL). The combined aqueous phase was concentrated under reduced pressure to obtain crude **ODN S10** as a white solid. Oligonucleotides were purified by reverse-phase HPLC on a Dionex UltiMate 3000 System using a Phenomenex Clarity Oligo-RP column (250 × 10 mm, 5 μm, 5 mL/min, A = 0.1 M TEAA in water (pH 7), B = 0.1 M TEAA, 80% MeCN in water (pH 7)), lyophilised and then desalted using a NAP-25 Sephadex column.

HPLC purity (Rt = 4.27 min): 98% (260 nm).

MALDI (ToF): *m*/*z* calcd for $C_{131}H_{164}N_{48}O_{73}P_{12}+H^+$: 3949.75 $[M+H]^+$; found: 3949.94.

Gradient for HPLC purification (A = 0.1 M TEAA in water (pH 7), B = 0.1 M TEAA, 80% MeCN in water (pH 7)):

**Time** (min)	**B%**

0 – 16	10 – 40
16 – 17	40 – 95
17 – 21	95
21 – 22	95 – 10
22 – 27	10

ODN 16

The first half of **ODN 16** was synthesised using the standard protocol described for **ODN S10**. The introduction of DBCO in the position 7 of the ODN was achieved using DBCO-dT-CE phosphoramidite (Glen Research 10-1539-02). The coupling time used was 12 min. DBCO-dT is susceptible to damage by iodine during oxidation. To prevent loss of the DBCO label during the second half of the ODN synthesis, 0.5 M 10-Camphorsulfonyl-Oxaziridine (CSO) in anhydrous acetonitrile (Glen Research 40-4632-52E) was use with a 3 min oxidation time.

For deprotection, cleavage and purification the used protocols were the same as described above for **ODN S10**.

HPLC purity (Rt = 6.10 min): 75% (260 nm)

MALDI (ToF): *m/z* calcd for $C_{160}H_{195}N_{51}O_{76}P_{12}$+$H^+$: 4418.99 [*M*+H]$^+$; found: 4419.73.

Gradient for HPLC purification (A = 0.1 M TEAA in water (pH 7), B = 0.1 M TEAA, 80% MeCN in water (pH 7)):

**Time** (min)	**B%**
0 – 1	35
1 – 11	35 – 40
11 – 12	40 – 95
12 – 14	95
14 – 15	95 – 35
15 – 20	35

Purity Analysis Method

Thermo Vanquish System

Column Specifications: Phenomenex bioZen™ Oligo column, 50 × 4.6 mm, 2.6 µm.

Column Temperature: 50 °C

Mobile Phase A: 0.1 M TEAA in water (pH 7)

Mobile Phase B: 0.1 M TEAA, 80% MeCN in water (pH 7)

Flow rate: 1.2 mL/min

Injection Volume: 10 µL

**Time** (min)	**B%**
0 – 0.5	5
0.5 – 6.8	5 – 50
6.8 – 7	50 – 95
7 – 8.2	95
8.2 – 8.3	95 – 5

8.3 – 9.5	5

The reaction conditions for the synthesis of **ODN S12**, **ODN 17** and **ODN 19** are described in section 2.3 Procedure for Oligonucleotide HPLC Assay.

2. HPLC Assay Protocol

2.1 Procedure for Small Molecule HPLC Assay

Stock solutions of $Cu(OAc)_2$ and GSH were prepared each day. Stock solutions of alkynes and azides were stored in the fridge and used up to 5 days.

Stock solutions:

[Alkyne] = 6 mM in organic solvent (*i.e.*, MeOH)

[Azide] = 30 mM in organic solvent (*i.e.*, MeOH)

[GSH] = 30 mM in 1X DPBS

[$Cu(OAc)_2$] = 10 mM in H_2O

[NaAsc] = 30 mM in 1X DPBS

Procedure:

Appropriate amounts of the stock solutions (see example) were added to reach specified concentrations to an HPLC vial and made up with MeOH and 1X DPBS buffer to a final volume of 1.5 mL. The addition order was as follows: MeOH, alkyne, benzyl azide, buffer, GSH, $Cu(OAc)_2$. The vials were then placed in the autosampler and sampled for the specified time.

Example:

Example of volumes added for the reactions in Figure 5(b).

Substrate	Desired Concentration (μM)	μL added from Stock Solution
Ynamine (**1a**)	200	50
Azide (**4a**)	500	25
Organic solvent	N/A	75
1X DPBS	N/A	1330
GSH	100	5
$Cu(OAc)_2$	100	15

Chromatography Conditions:

Column Specifications: Phenomenex Kinetex® C18, 50 x 4.6 mm, 2.6 μm

Column Temperature: 25 °C

Mobile Phase A: 0.1% TFA in water

Mobile Phase B: 0.1% TFA in MeCN

Flow rate: 1.5 mL/min

Gradient Profile:

**Time** (min)	**B%**
0 – 5	5 – 60
5 – 5.1	60 – 95
5.1 – 7.2	95
7.2 – 7.3	95 – 5
7.3 – 9.5	5

Conversion Calculations:

UV detection signal was recorded at 254 nm. Conversions were calculated by peak areas according to the following formula:

$$Conversion = \frac{Area\ (Product)}{Area\ (Starting\ Material) + Area\ (Product)} \times 100$$

Conversions for ynamine (**1a**) and corresponding triazole (**5a**), which were calculated using a calibration curve (Figure S1) according to the following formula:

$$Conversion = \frac{[Product]}{[Initial\ Starting\ Material] + [Initial\ Product]} \times 100$$

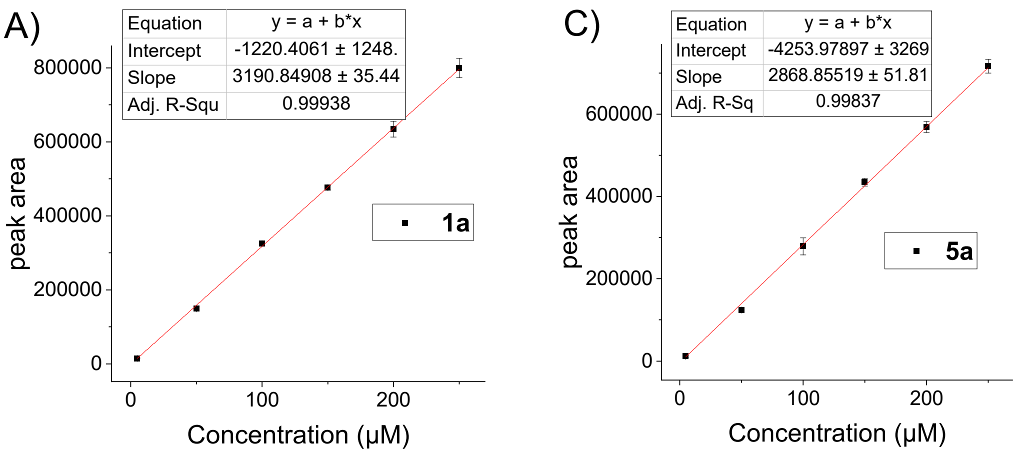

Figure S1. A) Calibration curve of ynamine (**1a**). B) Calibration curve of triazole (**5a**).

Exemplary Chromatograms

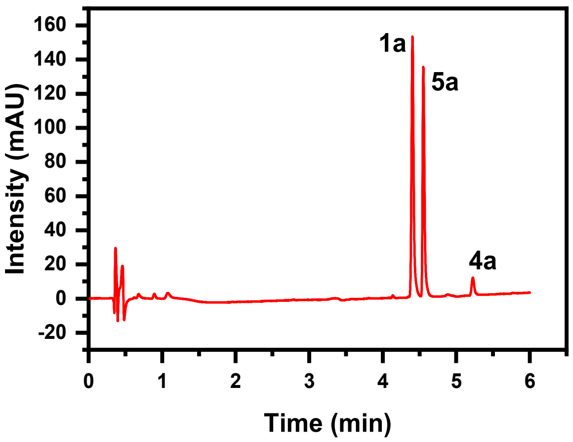

Figure S2. Example of a typical UV-trace obtained from the reaction of ynamine (**1a**) with benzyl azide (**4a**) to form triazole (**5a**) after 1 h. Only elution gradient shown (up to 6 min). No further compounds eluted after 6 min. *Conditions:* **1a** (200 µM), $Cu(OAc)_2$ (100 µM), GSH (100 µM), **4a** (500 µM), 10% MeOH in 1X DPBS, rt, 2.5 h. Buffer pH = 7.4.

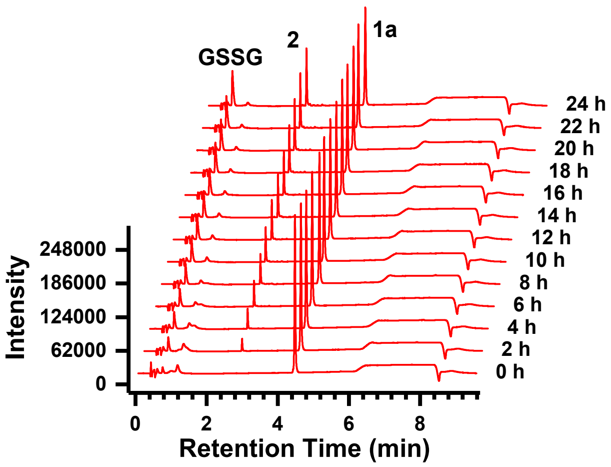

Figure S3. Waterfall plot of stacked chromatograms showing continuous production of GSSG and **2** in the presence of $Cu(OAc)_2$ (350 µM). *Conditions:* **1a** (200 µM), $Cu(OAc)_2$ (350 µM), GSH (10 mM), 10% MeOH in 1X DPBS, rt, 24 h. Buffer pH = 7.4.

2.2 Procedure for Peptide HPLC Assay

Stock solutions of $Cu(OAc)_2$ and GSH were prepared each day. Stock solutions of alkynes and azides were stored in the fridge and used up to 5 days.

The concentration of Penetratin-Az (**6**) was calculated using a Thermofisher Nanodrop (calculated extinction coefficient: 11080 M^{-1} cm^{-1}).[8] TP2-Az (**7**) and Pico-TP2-Az (**12**) were weighed out on a microbalance and diluted to the desired concentration (molecular weights were calculated as the triple TFA salt; **7** = 1981.08 Da and **12** = 2215.32 Da).

Stock solutions:

[Ynamine/DBCO (**8**/**11**)] = 4 mM in organic solvent

[Penetratin-Az (**6**)] = 1.3 mM in water

[TP2-Az (**7**)] = 2 mM in 1:1 H_2O/organic solvent

[GSH] = 4 mM in 1X DPBS

[$Cu(OAc)_2$] = 4 mM in H_2O

Procedure (Mono-labelling (Figure 6c) and Competition (Figure S13)):

Appropriate amounts of the stock solutions were added to reach the desired concentrations to an HPLC vial (polypropylene, v-shaped 200 µL) and made up with organic co-solvent and 1X DPBS buffer to a final volume of 200 µL. The addition order was as follows: ynamine, DBCO (if applicable), organic solvent, buffer, GSH, $Cu(OAc)_2$ and peptide. The vials were then placed in the autosampler and sampled for the specified time.

Example:

Example of volumes added in Figure S13(b).

Substrate	Desired Concentration (µM)	µL added from Stock Solution
Ynamine (**8**)	200	10
DBCO (**11**)	200	10
Organic solvent	N/A	-
1X DPBS	N/A	100
GSH	100	25
$Cu(OAc)_2$	100	25
Penetratin-Az (**6**)	200	30

Chromatography Conditions:

Column Specifications: Phenomenex Luna® Omega Polar C18 (100 × 4.6 mm, 3 µm).

Column Temperature: 40 °C

Mobile Phase A: 0.1% TFA in H_2O

Mobile Phase B: 0.1% TFA in MeCN

Injection volume: 10 µL

Flow rate: 1.2 mL/min

Gradient Profile:

**Time** (min)	**B%**
0 – 9	10 – 70
9 – 9.5	70 – 95
9.5 – 12	95
12 – 12.1	95 – 10

12.1 – 14.5	10

Conversion Calculations:

UV detection signal was recorded at 254 nm. Normalised product rations were calculated by peak areas according to the following formula:

$$Product\ Ratio = \frac{Area\ (Product)}{Area\ (Ynamine) + Area\ (Product)} \times 100$$

Exemplary chromatogram

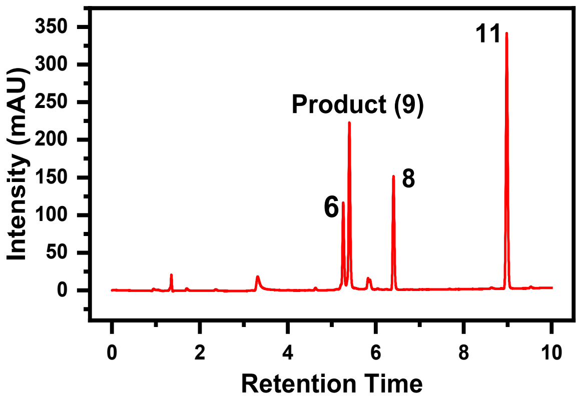

Figure S4. HPLC trace for the selective labelling of Penetratin-Az (**6**) with ynamine (**8**) in the presence of DBCO (**11**) after 1 h. *Conditions:* **6** (200 μM), **8** (200 μM), **11** (200 μM), $Cu(OAc)_2$ (500 μM), GSH (500 μM), 10% HFIP in 1X DPBS, rt, 4 h.

Procedure (Sequential-labelling; experiments with Pico-TP2-Az (12)):

Appropriate amounts of the stock solutions were added to reach the desired concentrations to an HPLC vial (polypropylene, v-shaped 200 μL) and made up with HFIP and 1X DPBS buffer to a final volume of 100 μL. The addition order was as follows: ynamine, DBCO, GSH, $Cu(OAc)_2$, 5 – 10 min wait, HFIP, 1X DBPS and Pico-TP2-Az (**13**). The vials were then placed in the autosampler and sampled for the specified time. Then a EDTA solution (10% wt, pH 10) was added after 2 h.

Example (Sequential-labelling):

Example of volumes used for reaction in Figure 7(b).

Substrate	Desired Concentration (μM)	μL added from Stock Solution
Ynamine (**8**)	220	5.5
DBCO (**11**)	220	5.5
Organic solvent	N/A	4
1X DPBS	N/A	50

GSH	500	12.5
$Cu(OAc)_2$	500	12.5
Pico-TP2-Az (**12**)	200	10
EDTA (10% wt)	5000	1.5

Chromatography Conditions:

Column Specifications: Phenomenex Luna® Omega Polar C18 (100 × 4.6 mm, 3 μm).

Column Temperature: 40 °C

Mobile Phase A: 0.1 % v/ v TFA in H_2O

Mobile Phase B: 0.1 % v/ v TFA in MeCN

Injection volume: 5 μL

Flow rate: 1.2 mL/min

Gradient Profile:

**Time** (min)	**B%**
0 – 9	30 – 70
9 – 9.5	70 – 95
9.5 – 12	95
12 – 12.1	95 – 30
12.1 – 14.5	30

Conversion Calculations:

The product ratio for the sequential labelling of Pico-TP2-Az (**12**) were calculated according to the to the following formula (all products = mono-ynamine (**13**) and DBCO (M-DBCO) labelled peptide, double-ynamine (D-YNA) and double DBCO (D-DBCO) labelled peptide and desired product (**15**)):

$$Product\ Ratio = \frac{Area\ (Product)}{Area\ (Peptide) + Area\ (All\ Products)} \times 100$$

Exemplary Chromatograms:

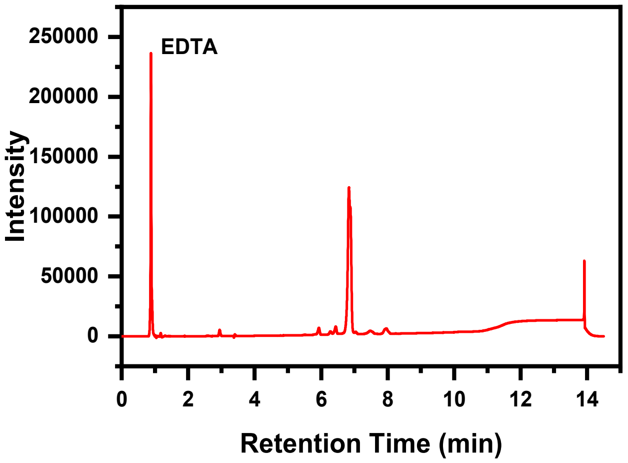

Figure S5. Typical UV-trace (HPLC) obtained after the completion of the sequential labelling of Pico-TP2-Az (**12**) with ynamine (**8**) and DBCO (**11**). *Conditions:* **8** (220 µM), **11** (220 µM), **12** (200 µM), $Cu(OAc)_2$ (500 µM), GSH (500 µM), 10% HFIP in 1X DPBS, rt, 6 h.

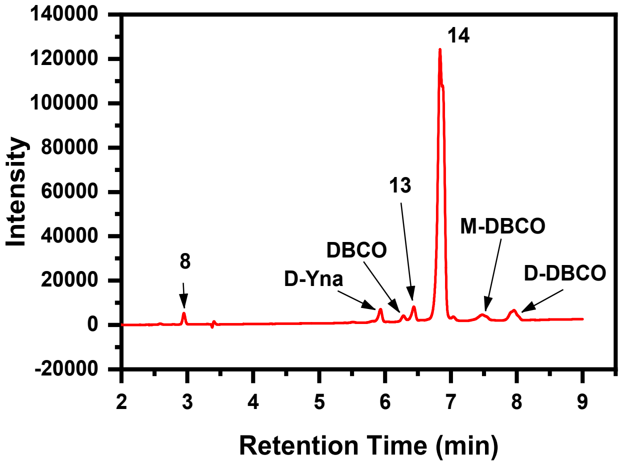

Figure S6. Zoom in (2 – 9 min) of the UV-trace (HPLC) obtained after the completion of the sequential labelling of Pico-TP2-Az (**12**) with ynamine (**8**) and DBCO (**11**). Identity of species were confirmed by UPLC-MS or injection of standards.

2.3 Procedure for Oligonucleotide HPLC Assay

After purification and desalting the oligonucletiodes were dissolved in ultrapure H_2O (MiliQ) and the concentration measured using a Thermofisher Nanodrop (extinction coefficients: 112700 M^{-1} cm^{-1} (**S10**) and 122336 M^{-1} cm^{-1} (**16**); calculated using IDT OligoAnalyzer™ Tool and stated extinction coefficient (Glen Research) of DBCO-dT-CE Phosphoramidite (18800 M^{-1} cm^{-1}).

1X DPBS (20 mM $MgCl_2$) was prepared by adding 39.7 mg of $MgCl_2$•$6H_2O$ to a volumetric flask and adding 1X DPBS up to the 10 mL mark. Stock solutions of $Cu(OAc)_2$ and GSH were prepared each day. Stock solutions of alkynes and azides were kept in the freezer and used up to 5 days.

Stock solutions:

[ODN (**S10**)] = 0.35 mM in H_2O

[ODN (**16**)] = 0.31 mM in H_2O

[NBD azide (**15**)] = 2 mM in HFIP

[Biotin azide (**18**)] = 2.5 mM in 1X DPBS (20 mM $MgCl_2$)

[GSH] = 4 mM in 1X DPBS (20 mM $MgCl_2$)

[$Cu(OAc)_2$] = 4 mM in H_2O

Procedure (ODN (S10) labelling with NBD azide (15)

Appropriate amounts of the stock solutions were added to reach the desired concentrations to an HPLC vial (polypropylene, v-shaped 200 µL) and made up with HFIP and buffer to a final volume of 100 µL. The addition order was as follows: ODN (**S10**), GSH, $Cu(OAc)_2$, buffer, HFIP, azide (**15**). The vials were then placed in the autosampler and sampled for the specified time.

Example:

Example of volumes added for the reaction in Figure S15(b). Note: the calculated extinction coefficients do not account for the ynamine moiety, therefore the addition volume of the oligonucleotide stock was increased by ~15% (from calculated 4.3 µL to 5 µL).

Substrate	Desired Concentration (µM)	µL added from Stock Solution
ODN (**S10**)	20	5
NBD azide (**15**)	50	2.5
HFIP	N/A	2.5
buffer	N/A	87.5
GSH	50	1.25
$Cu(OAc)_2$	50	1.25

Chromatography Conditions:

Column Specifications: Phenomenex bioZen™ Oligo column (50 × 4.6 mm, 2.6 µm).

Column Temperature: 60 °C

Mobile Phase A: 0.1 M TEAA in H_2O (pH 7)

Mobile Phase B: 0.1 M TEAA, 80% MeCN in H_2O (pH 7)

Injection volume: 3 µL

Flow rate: 1.5 mL/min

Gradient Profile:

**Time** (min)	**B%**
0 – 0.5	10
0.5 – 7	10 – 95
7 – 8.2	95
8.2 – 8.3	95 – 10
8.3 – 9.5	10

Calculation of Conversion:

The conversion for the reaction of ODN (**S10**) and NBD azide (**15**) was calculated according to the to the following formula:

$$Conversion = \frac{Area\ (Product)}{Area\ (Starting\ Material) + Area\ (Product)} \times 100$$

Exemplary chromatogram

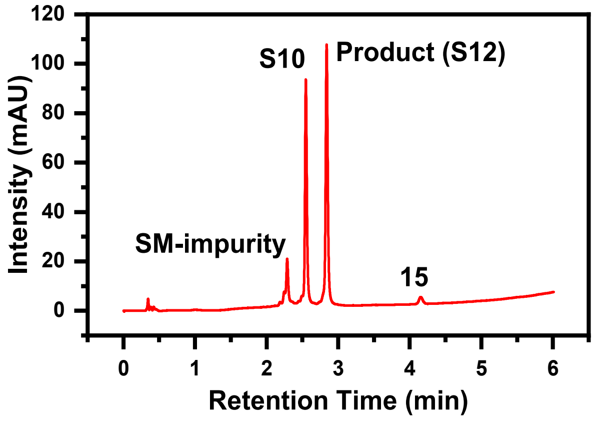

Figure S7. HPLC trace for the reaction of ODN (**S10**) with NBD azide (**15**) after 30 minutes. Reaction complete after ~2 h. *Conditions:* **S10** (20 µM), **15** (50 µM), $Cu(OAc)_2$ (50 µM), GSH (50 µM), 5% HFIP in 1X DPBS (20 mM $MgCl_2$), rt, 4 h.

Procedure (Sequential labelling of ODN (16)):

Appropriate amounts of the stock solutions were added to reach the desired concentrations to an HPLC vial (polypropylene, v-shaped 200 µL) and made up with HFIP and buffer to a final volume of 100 µL. The addition order was as follows: NBD azide (**15**), HFIP, buffer and ODN (**16**). The vials were then placed in the autosampler and sampled for the specified time. Then after 1 h: GSH, $Cu(OAc)_2$, and biotin azide (**18**) were added.

Example:

Example of volumes added for the reaction in Figure 8(b). Note: due to degradation issues of DBCO the addition volume of the oligonucleotide stock was increased by ~35% (from calculated 6.4 μL to 10 μL) to achieve the desired concentration.

**Substrate**	**Desired Concentration (μM)**	**μL added from Stock Solution**
ODN (**16**)	20	10
NBD azide (**15**)	20	1
HFIP	N/A	4
buffer	N/A	85
GSH	50	1.25
$Cu(OAc)_2$	50	1.25
Biotin azide (**18**)	30	1.2

Chromatography Conditions:

Column Specifications: Phenomenex bioZen™ Oligo column (50 × 4.6 mm, 2.6 μm).

Column Temperature: 50 °C

Mobile Phase A: 0.1 M TEAA in H_2O (pH 7)

Mobile Phase B: 0.1 M TEAA, 80% MeCN in H_2O (pH 7)

Injection volume: 3 μL

Flow rate: 1.5 mL/min

Gradient Profile:

**Time** (min)	**B%**
0 – 0.5	22
0.5 – 6	22 – 45
6 – 6.1	45 – 95
6.1 – 7.5	95
7.5 – 7.6	95 – 22
7.6 – 9.5	22

Calculation of Conversion:

The product ratio for the reaction of oligonucleotide (**16**) and NBD azide (**15**) were calculated according to the to the following formula:

$$Product\ Ratio = \frac{Area\ (Product)}{Area\ (SM) + Area\ (Product\ mono) + Area\ (Product\ final)} \times 100$$

Exemplary Chromatogram

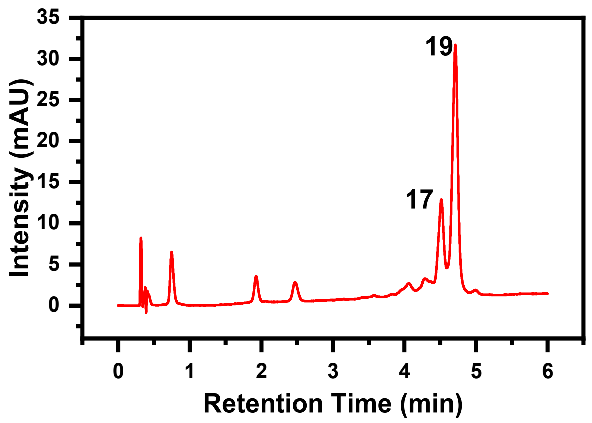

Figure S8. Exemplary HPLC trace for the sequential labelling of ODN (**16**) after 1.66 h. *Conditions:* **16** (20 µM), **15** (20 µM), $Cu(OAc)_2$ (50 µM), GSH (50 µM), **18** (30 µM), 5% HFIP in 1X DPBS (20 mM $MgCl_2$), rt, 3 h.

3. Supplementary Figures

3.1 HPLC data

Influence of Cu(II) on the Stability of Ynamine (1a) in the Presence of GSH

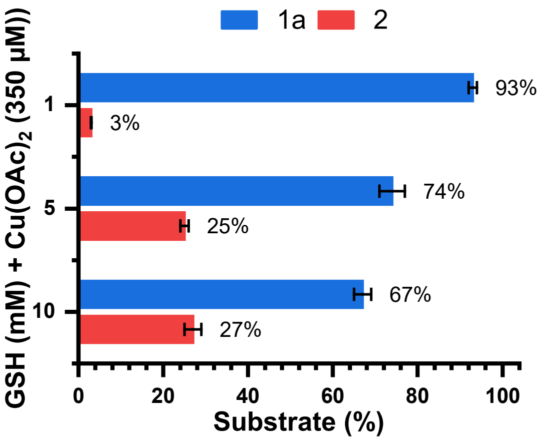

Figure S9. Influence of $Cu(OAc)_2$ (350 µM) addition on ynamine **1a** stability in the presence of GSH (1, 5 and 10 mM). *Conditions:* **1a** (200 µM), $Cu(OAc)_2$ (350 µM), GSH (1, 5 or 10 mM), 10% MeOH in 1X DPBS, rt, 24 h.

Influence of [GSH] on the CuAAC Reaction of Ynamine (1a) with Azide (4a)

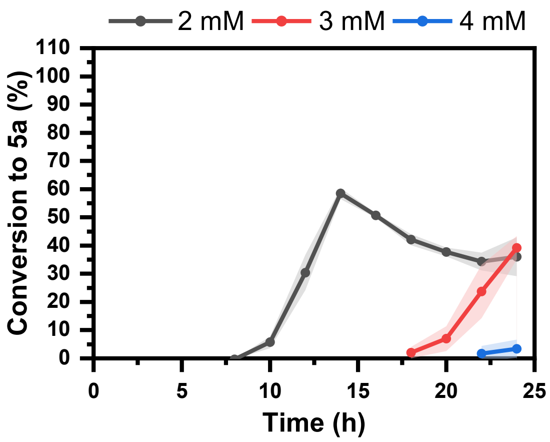

Figure S10. Effect of increasing GSH concentrations (2 – 4 mM) on the reaction of ynamine (**1a**) with benzyl azide (**4a**). Higher concentrations lead to a prolonged induction period. *Conditions:* **1a** (200 µM), $Cu(OAc)_2$ (350 µM), **4a** (500 µM), GSH (2 – 4 mM), 10% MeOH in 1X DPBS, rt, 24 h. Shaded areas represent the standard deviation calculated from three experiments.

Influence of NaAsc on Ynamine (1a) Reactivity with different Azides

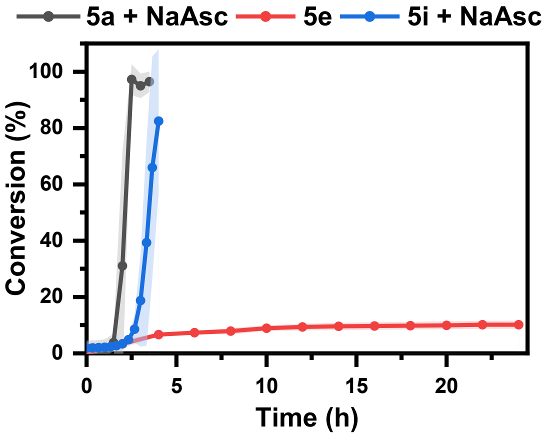

Figure S11. Effect of NaAsc addition on ynamine reactivity. NaAsc addition slightly slows reactivity of ynamine (**1a**) with benzyl azide (**4a**) and picolyl azide (**4c**). However, reaction with azido ethanol (**5e**) is sluggish without NaAsc addition (compared with Figure 5b). Conditions: **1a** (200 μM), $Cu(OAc)_2$ (350 μM), **4a**/**4b**/**4c** (500 μM), GSH (1 mM), NaAsc (1 mM, if applicable), 10% MeOH in 1X DPBS, rt, 3.5-24 h. Shaded areas represent the standard deviation calculated from three experiments.

Time courses of the DoE Experiments

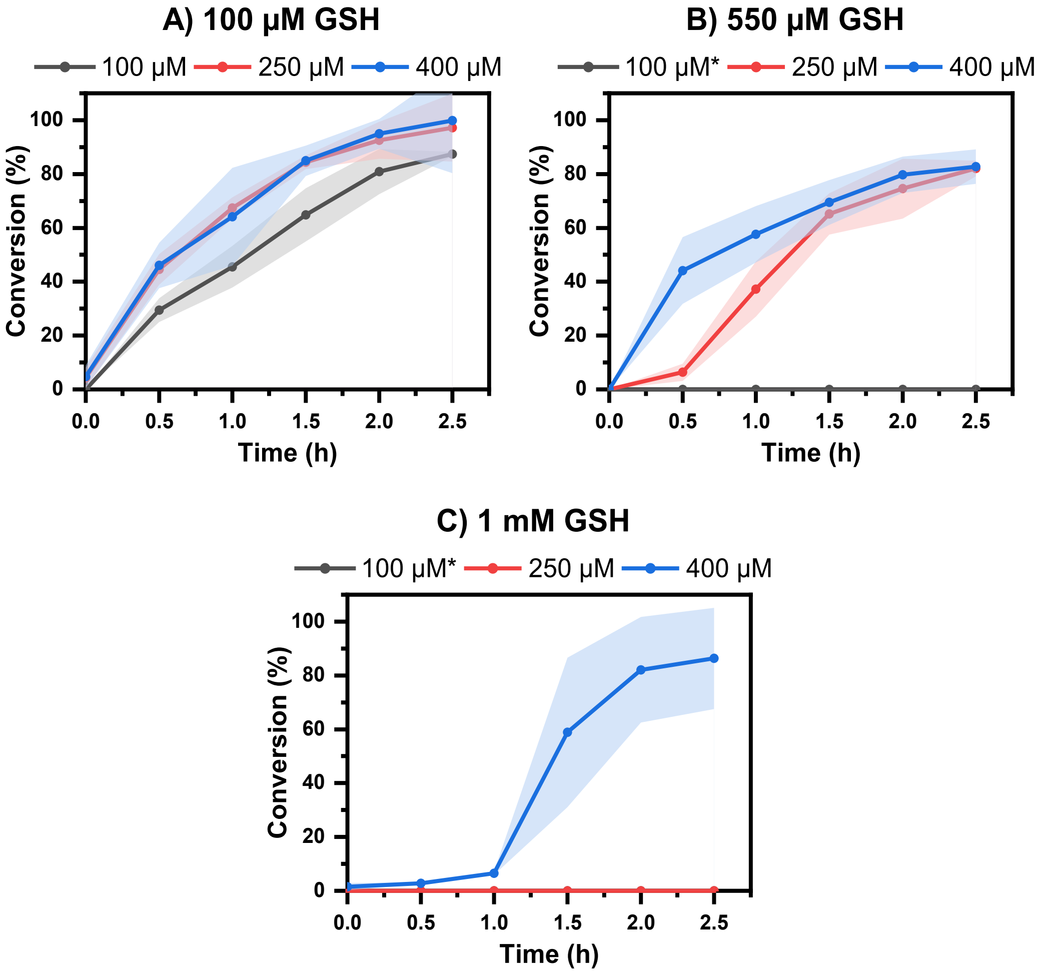

Figure S12. (A) Reaction of ynamine **1a** with benzyl azide (**4a**) using varied $Cu(OAc)_2$ (100, 250 and 400 µM) concentration in the presence of GSH (100 µM). *Conditions:* **1a** (200 µM), $Cu(OAc)_2$ (100, 250 or 400 µM), **4a** (500 µM), GSH (100 µM), 10% MeOH in 1X DPBS, rt, 2.5 h. (B) Reaction of ynamine **1a** with benzyl azide (**4a**) using varied $Cu(OAc)_2$ (100, 250 and 400 µM) concentration in the presence of GSH (550 µM). *No conversion observed, overlaps with X-axis. *Conditions:* **1a** (200 µM), $Cu(OAc)_2$ (100, 250 or 400 µM), **4a** (500 µM), GSH (550 µM), 10% MeOH in 1X DPBS, rt, 2.5 h. (C) Reaction of ynamine **1a** with benzyl azide (**4a**) using varied $Cu(OAc)_2$ (100, 250 and 400 µM) concentration in the presence of GSH (1 mM). *Conditions:* **1a** (200 µM), $Cu(OAc)_2$ (100, 250 or 400 µM), **4a** (500 µM), GSH (1 mM), 10% MeOH in 1X DPBS, rt, 2.5 h. Buffer pH = 7.4. Shaded areas represent the standard deviation calculated from three experiments. *No conversion observed, overlaps with X-axis.

Influence of Copper Source

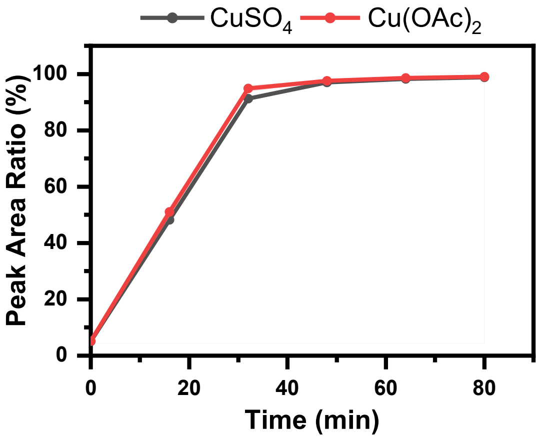

Figure S13. Reaction of ynamine **1a** with benzyl azide (**4a**) using either $CuSO_4$ or $Cu(OAc)_2$. *Conditions:* **1a** (200 μM), $Cu(OAc)_2$ (250 μM), **4a** (500 μM), GSH (100 μM), 10% MeOH in 1X DPBS, rt, 80 min. Shaded areas represent the standard deviation calculated from two experiments (deviation >1% so error bands are difficult to observe).

HFIP as CuAAC solvent

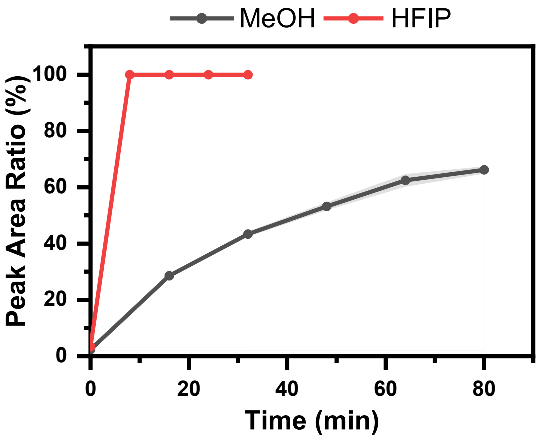

Figure S14. Reaction of alkyne **1d** with benzyl azide (**4a**) using either MeOH or HFIP as the co-solvent. *Conditions:* **1d** (200 μM), $Cu(OAc)_2$ (250 μM), **4a** (500 μM), NaAsc (1 mM), 10% MeOH or 10% HFIP in 1X DPBS, rt, 80 min. Shaded areas represent the standard deviation calculated from two experiments.

Controlling CuAAC and SPAAC Reactivity via the addition of GSH

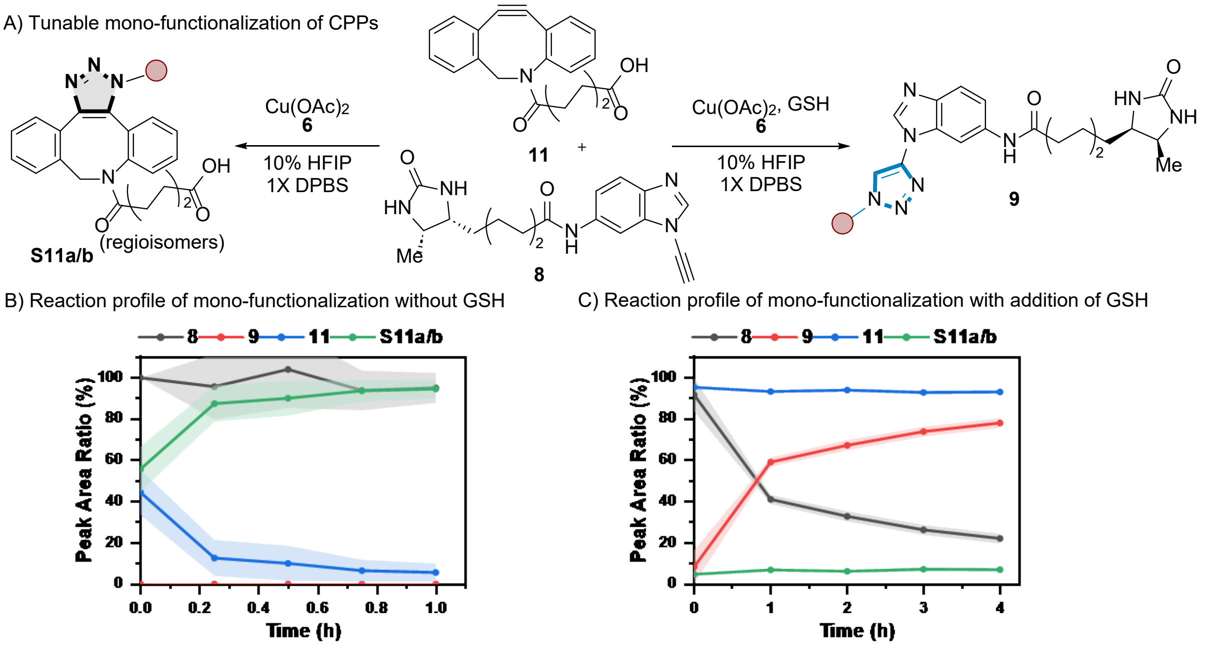

Figure S15. (A) SPAAC reactivity is inhibited by the addition of GSH. Therefore ynamine **8** can be selectively reacted with azide **6** in the presence of DBCO **11**. (B) GSH is not added to reaction and DBCO **11** reacts with azide **6** to form **S11a/b**. *Reaction Conditions:* **8** (200 μM), **11** (200 μM), **6** (200 μM), $Cu(OAc)_2$ (500 μM),10% HFIP in 1X DPBS, rt, 1 h. (C) Addition of GSH inhibits SPAAC reactivity and ynamine reacts with azide **6** to form triazole **9**. *Reaction Conditions:* **8** (200 μM), **11** (200 μM), **6** (200 μM), $Cu(OAc)_2$ (500 μM), GSH (500 μM), 10% HFIP in 1X DPBS, rt, 1 h.

Influence of Solvent % on the Selectivity of labelling Peptide 12

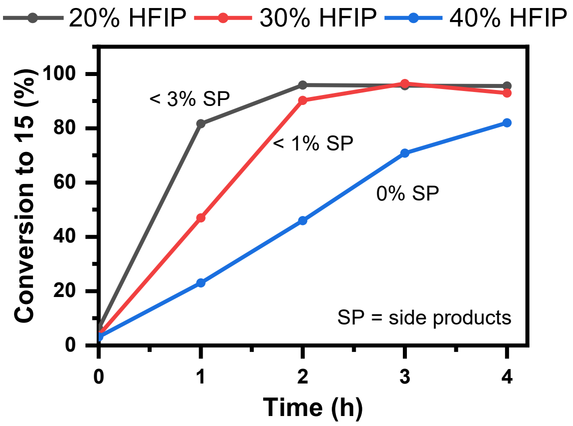

Figure S16. Reaction of ynamine (**8**) with Pico-TP2-Az (**12**) using different percentages of HFIP as co-solvent. Higher percentages slow the reaction and increase selectivity. 20% HFIP was chosen as a good compromise of reactivity and selectivity. Using only 10% HFIP more than 25% side product (SP, double ynamine labelled peptide) was observed (data not shown). *Conditions:* **8** (200 μM), $Cu(OAc)_2$ (500 μM), **12** (200 μM), GSH (500 μM), 20-40% HFIP in 1X DPBS, rt, 4 h.

Fluorescent labelling of ODN S9

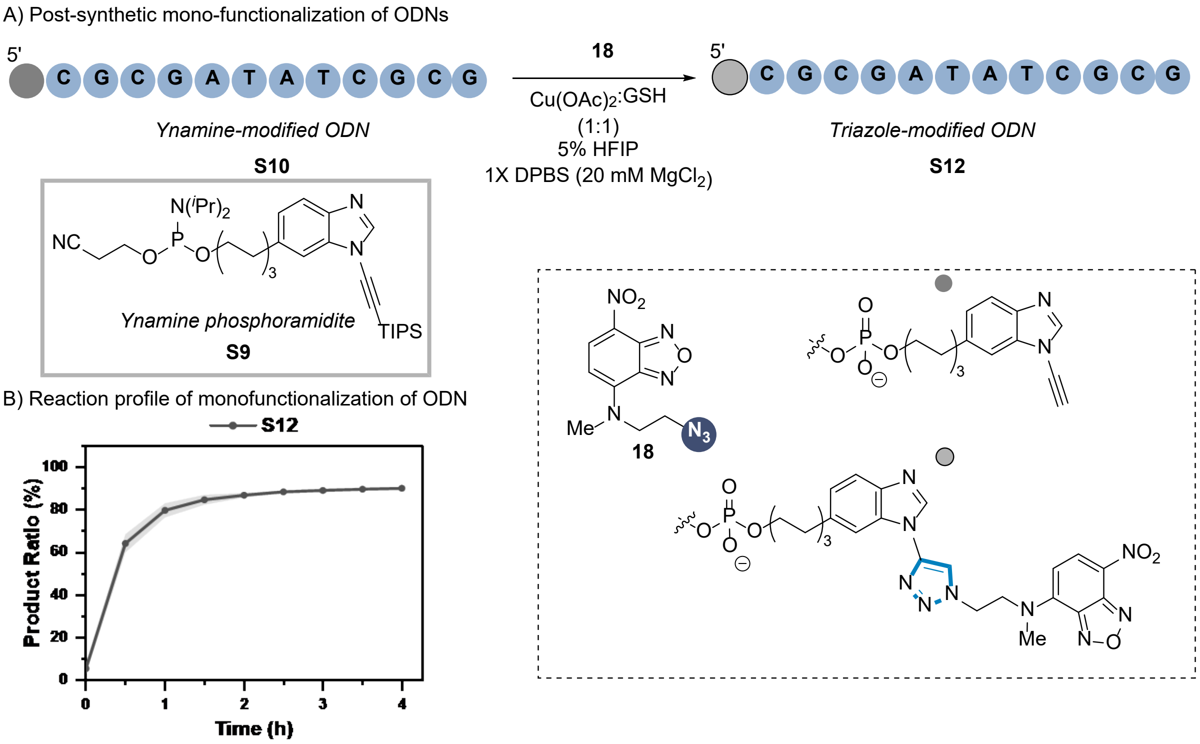

Figure S17. (A) Modification of monofunctionalized ODN (**S10**) with NBD azide (**15**) to form **S12**. (B) Reaction profile showing the rate of conversion of **S10** to **S12**. *Reaction conditions:* **S10** (20 µM), **15** (50 µM), $Cu(OAc)_2$ (50 µM), GSH (50 µM), 5% HFIP in 1X DPBS (20 mM $MgCl_2$), rt, 4 h.

3.2 EPR-Data

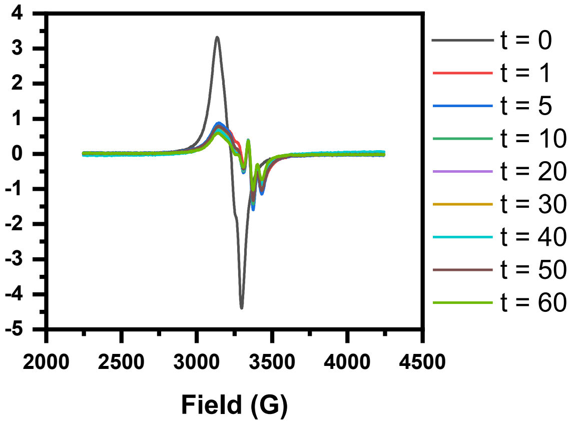

Figure S18. Stacked EPR spectra for the reaction ynamine (**1a**) with benzyl azide (**4a**) in MeOH catalysed by $Cu(OAc)_2$ and GSH (1:1). *Procedure:* **1a** (0.9 mg, 5 µmol, 1 equiv), GSH (0.8 mg, 2.5 µmol, 0.5 equiv) and **4a** (1.7 mg, 12.5 µmol, 2.5 equiv) were added to a vial to which a $Cu(OAc)_2$•H_2O solution (Water:MeOH 9:1) (5 mM, 0.5 mL, 2.5 µmol, 0.5 equiv) was added. Aliquots were then drawn into a 20 µL micropipette over time. *Note:* the same procedure was used to obtain the data for graph in Figure 5c, but MeOH was replaced with HFIP.

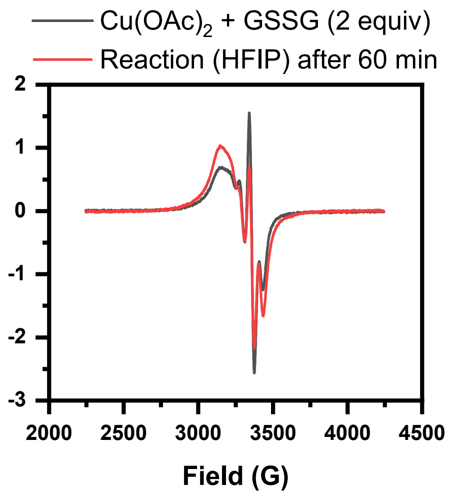

Figure S19. Graph comparing the EPR spectrum of $Cu(OAc)_2$ complex with the Ynamine-CuAAC reaction after 120 min. *Procedure:* $Cu(OAc)_2$•H_2O (1.0 mg, 5 µmol) and GSSG (6.1 mg, 10 µmol, 2 equiv) were added to a 1 mL volumetric flask and Water:HFIP (9:1) added. From this solution a 20 µL micropipette was filled and analysed.

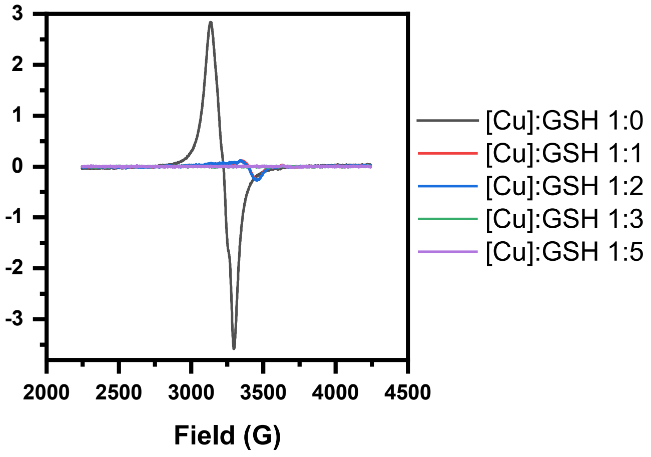

Figure S20. $Cu(OAc)_2$ mediated reduction by GSH. Full reduction of Cu(II) is observed when [Cu]:GSH > 1:3. *Procedure:* A stock solution of $Cu(OAc)_2$•H_2O (5 mM) was prepared in a 5 mL volumetric flask with Water:MeOH (9:1). From this stock solution, 0.5 mL was added to 1 mL vials containing varying equivalents of GSH. This was mixed for 15 mins before an aliquot was drawn into a 20 µL micropipette. (0.8 mg, 2.5 µmol, 1 equiv), (1.5 mg, 5.0 µmol, 2 equiv), (2.3 mg, 7.5 µmol, 3 equiv), (3.8 mg, 12.5 µmol, 5 equiv).

3.3 Supplementary UPLC-MS Data

UPLC-MS data for sequential labelling of peptide 12

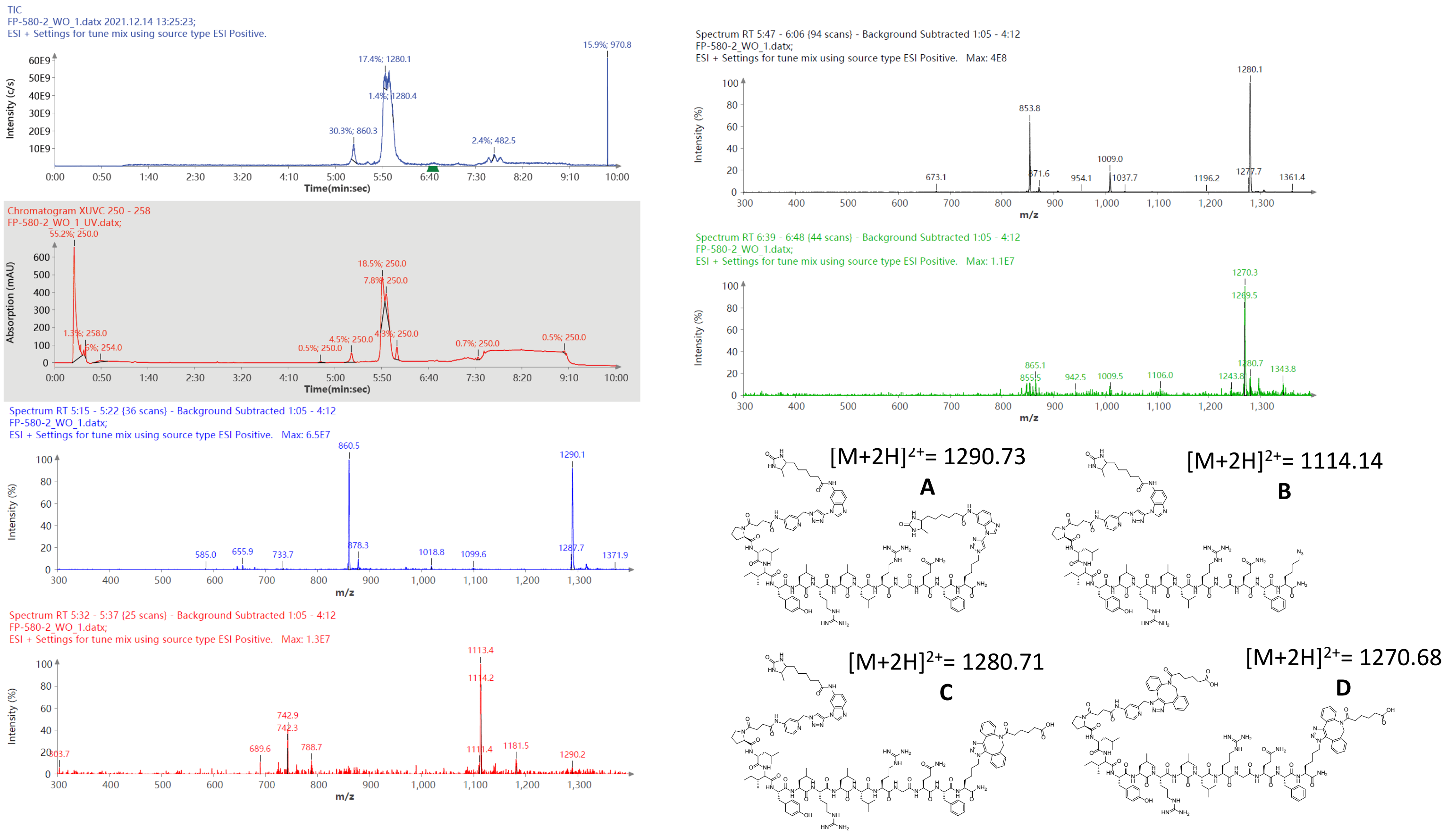

Figure S21. UPLC-MS traces after the completed sequential labelling of Pico-TP2-Az (**12**). *Conditions:* after reaction completion the solvent was removed on the SpeedVac™ and the residue redissolve in H_2O/MeCN (1:1, 0.1% formic acid, 100 uL) and analysed on an Advion UPLC coupled with a CMS Expression L mass spectrometer (ESI). Gradient elution using H_2O/MeCN (0.1% formic acid) on a Phenomenex C18 Luna® Polar column (100 × 2.1 mm, 1.6 µm).

UPLC-MS data for the digestion of peptide 12

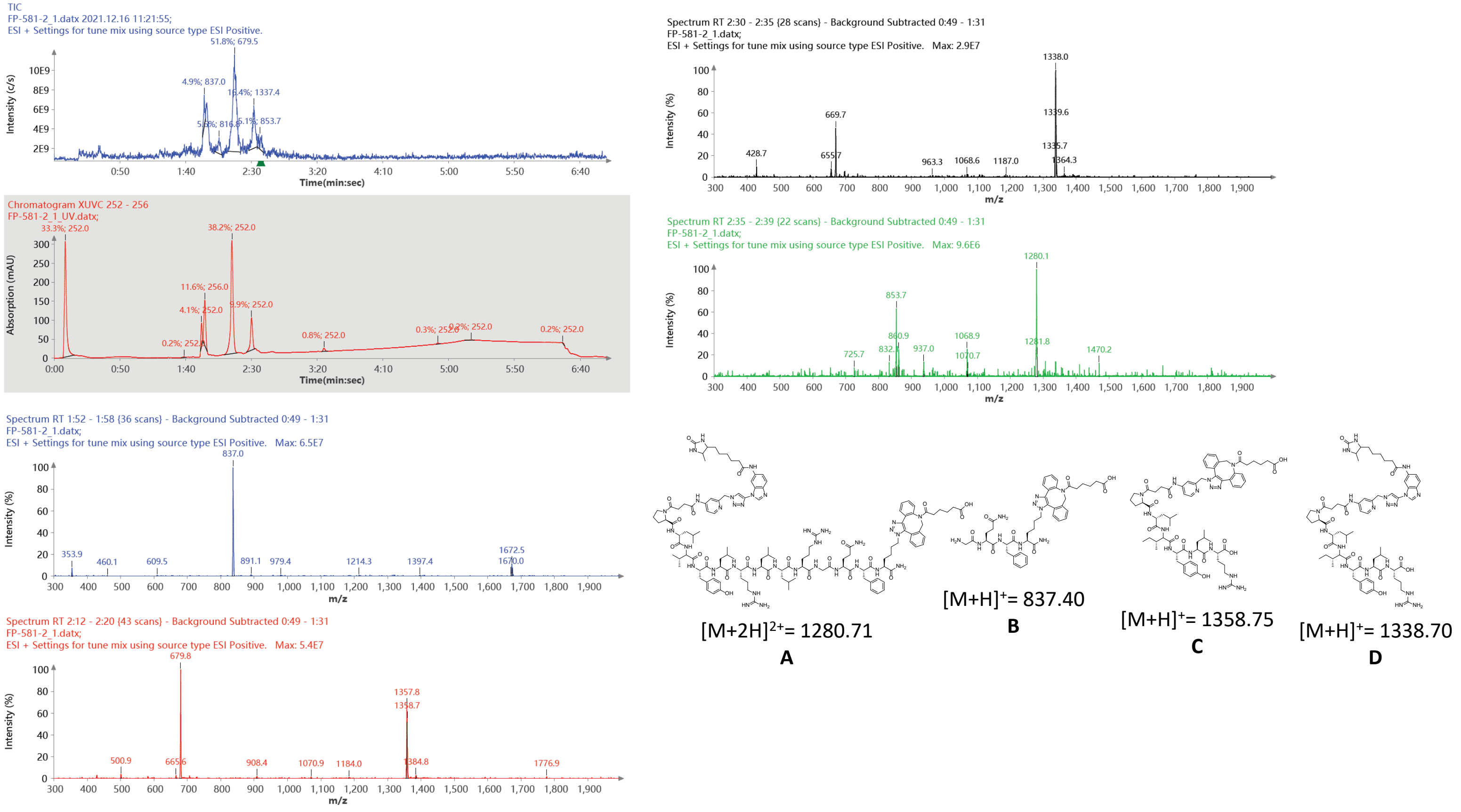

Figure S22. UPLC-MS traces after digestion of the sequential dual labelling of Pico-TP2-Az (**12**) with trypsin. *Conditions*: After the completion of the reaction, the solvent was removed using a SpeedVac™. The residue was redissolved in MeOH (20 µL), 1X DPBS (75 µL) and trypsin (5 µL, 1 mg/mL in H_2O) and left on a shaker overnight at 37 °C. The solvent was then removed using a SpeedVac™ and the residue dissolved in H_2O/MeCN (0.1% formic acid, 100 µL) and analysed on an Advion UPLC coupled with a CMS Expression L mass spectrometer (ESI). Gradient elution using H_2O/MeCN (0.1% formic acid) on a Phenomenex C18 Kinetex® column (30 × 2.1 mm, 2.6 µm).

MALDI data for the Enzymatic Digestion of ODN (19)

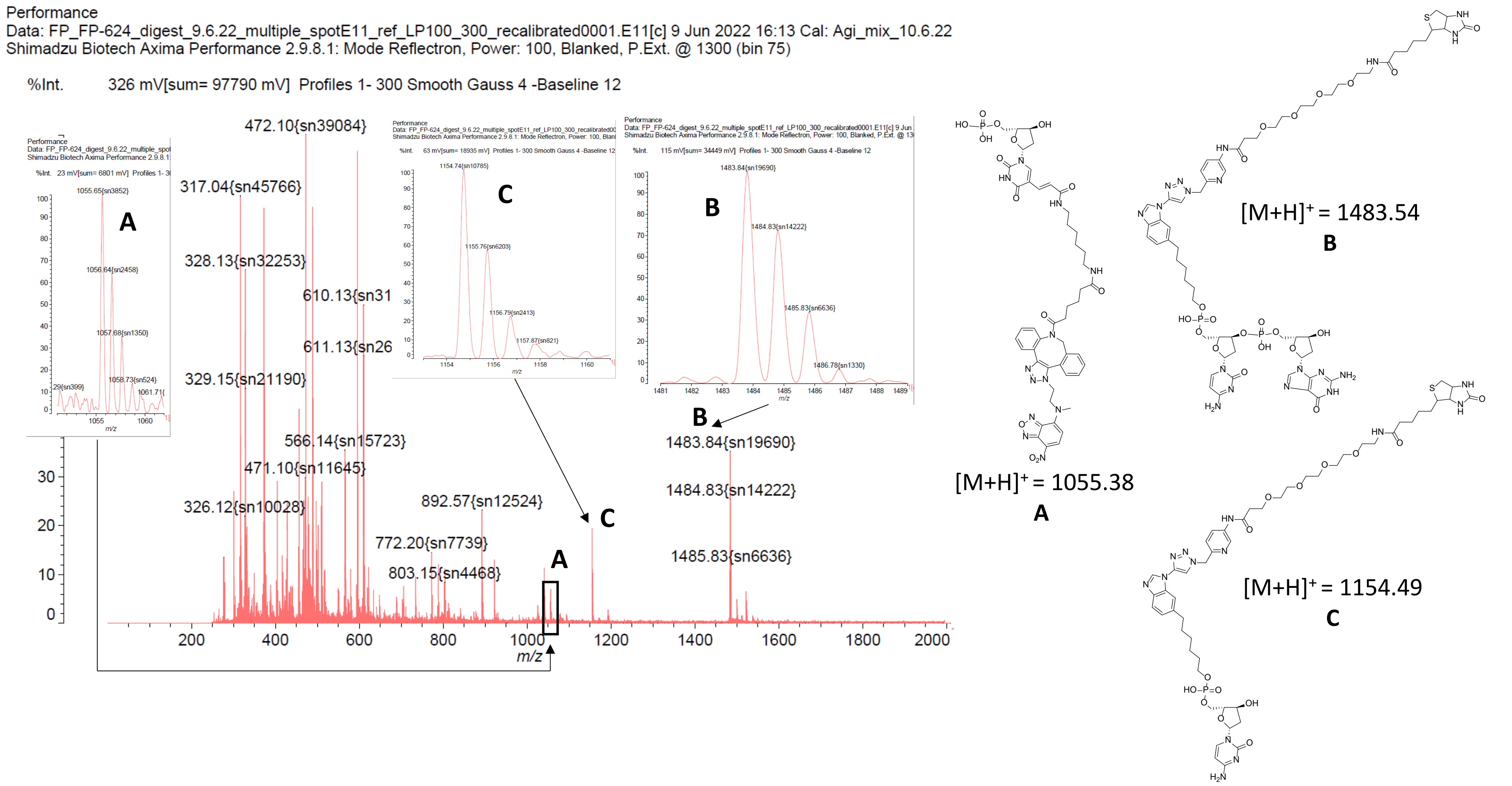

Figure S23. ODN (**19**) was purified by reverse phases chromatography and lyophilised. The residue was redissolved in purified water (45 μL) and 5 μL 10X reaction buffer was added followed by 0.2 μL of Exonuclease I (Thermo Scientific). The mixture was shaken at 37 °C for 1.5 h, then 0.2 μL of Exonuclease III (Thermo Scientific) was added. The

mixture was again shaken 37 °C for 1.5 h, then chloroform (50 µL) was added, and the mixture was vortexed for 2 min, then centrifuged for 5 min (13.4k rpm). The aqueous solution was pipetted off and again extracted with chloroform (50 µL, vortexed and centrifuged). The aqueous solution was pipetted into a fresh Eppendorf™ tube (0.5 mL DNA LoBind®). From the aqueous solution 5 µL were taken and mixed with 5 µL of 0.1 M TEAA in H_2O. The solution was desalted using a Ziptip® according to the manufacturer's instructions (Technical Note 225: Sample Preparation of Oligonucleotides Prior to MALDI-TOF MS using $ZipTip_{C18}$ and $ZipTip_{\mu\text{-}C18}$ Pipette Tips) and eluted with 5 µL (H_2O/MeCN = 1:1) and subsequently analysed by MALDI-TOF (Shimadzu Axima Performance™; Reflectron mode; matrix: 3-HPA (50 mg/mL), ammonium citrate dibasic (50 mg/ml) in H_2O/MeCN = 1:1).

4. Experimental Spectra

NMR spectra of compound **1a**

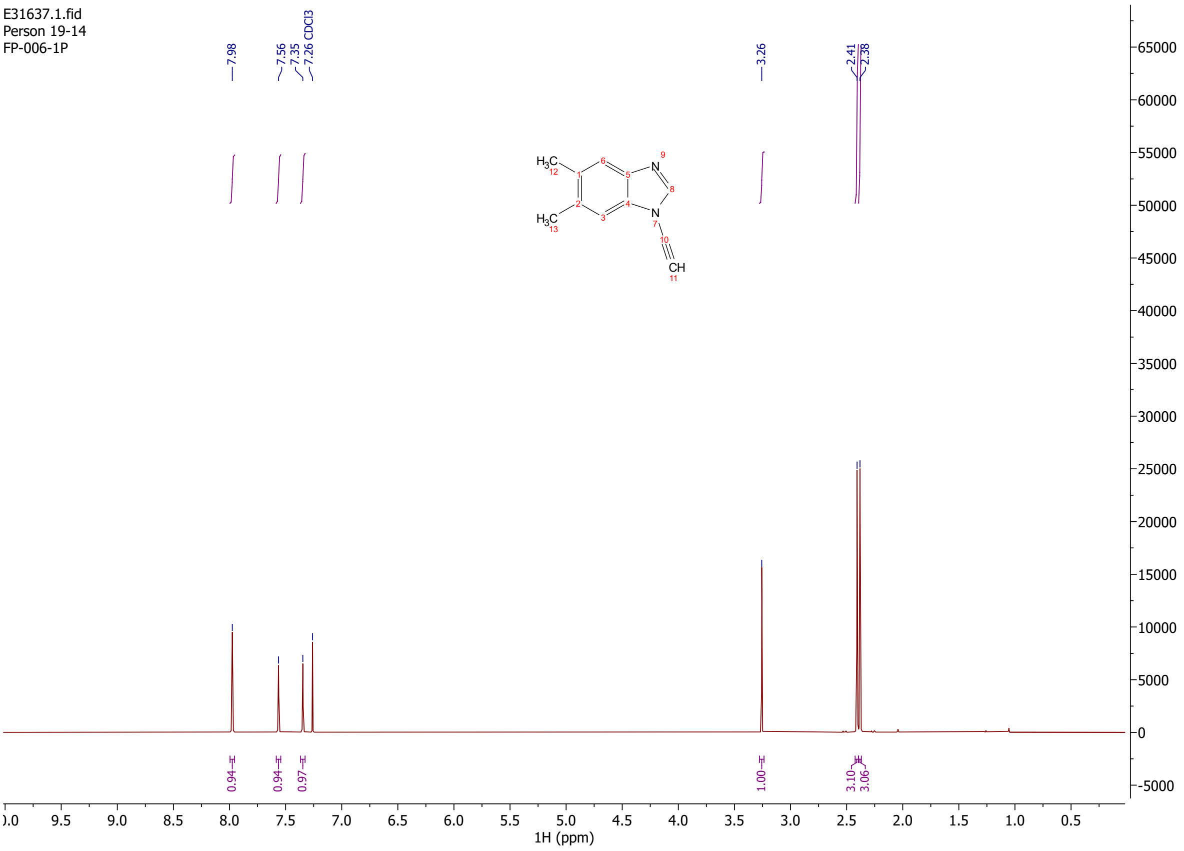

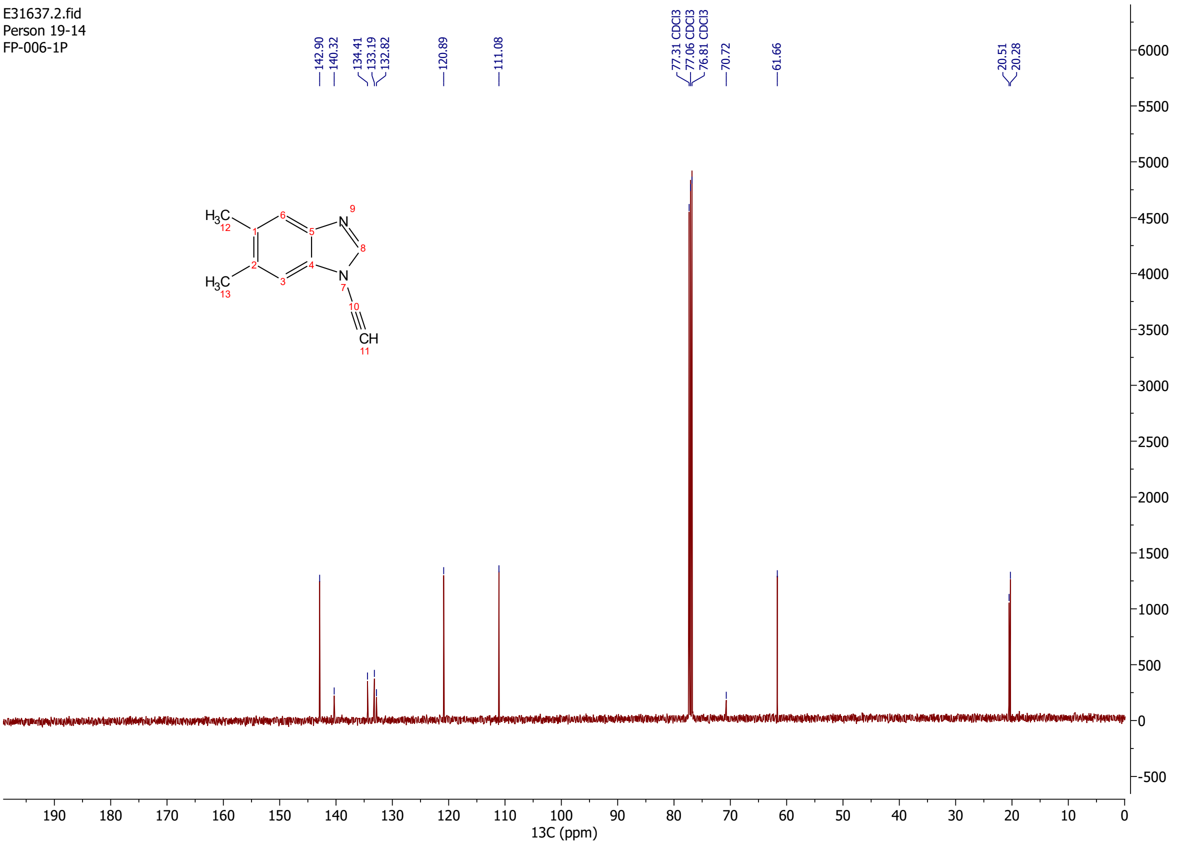

NMR spectra of compound **2**

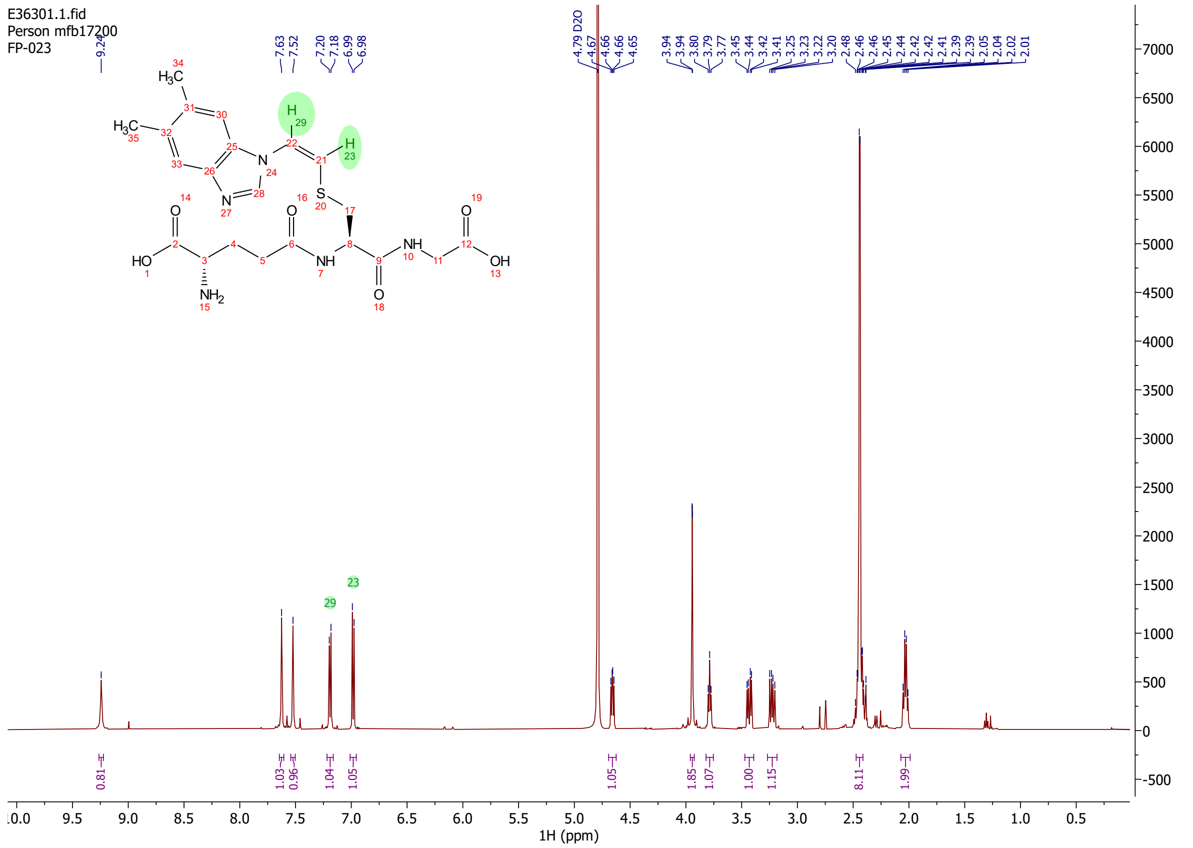

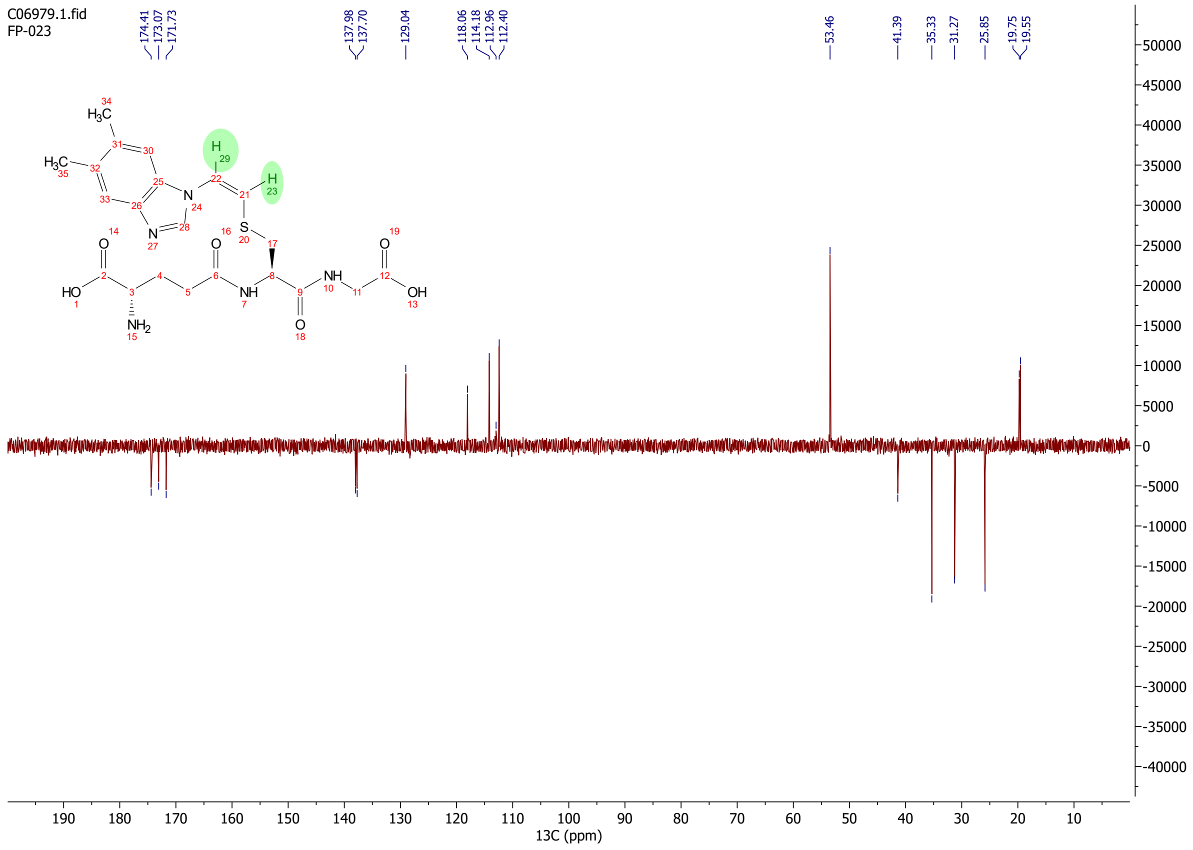

NMR spectrum of compound **3**

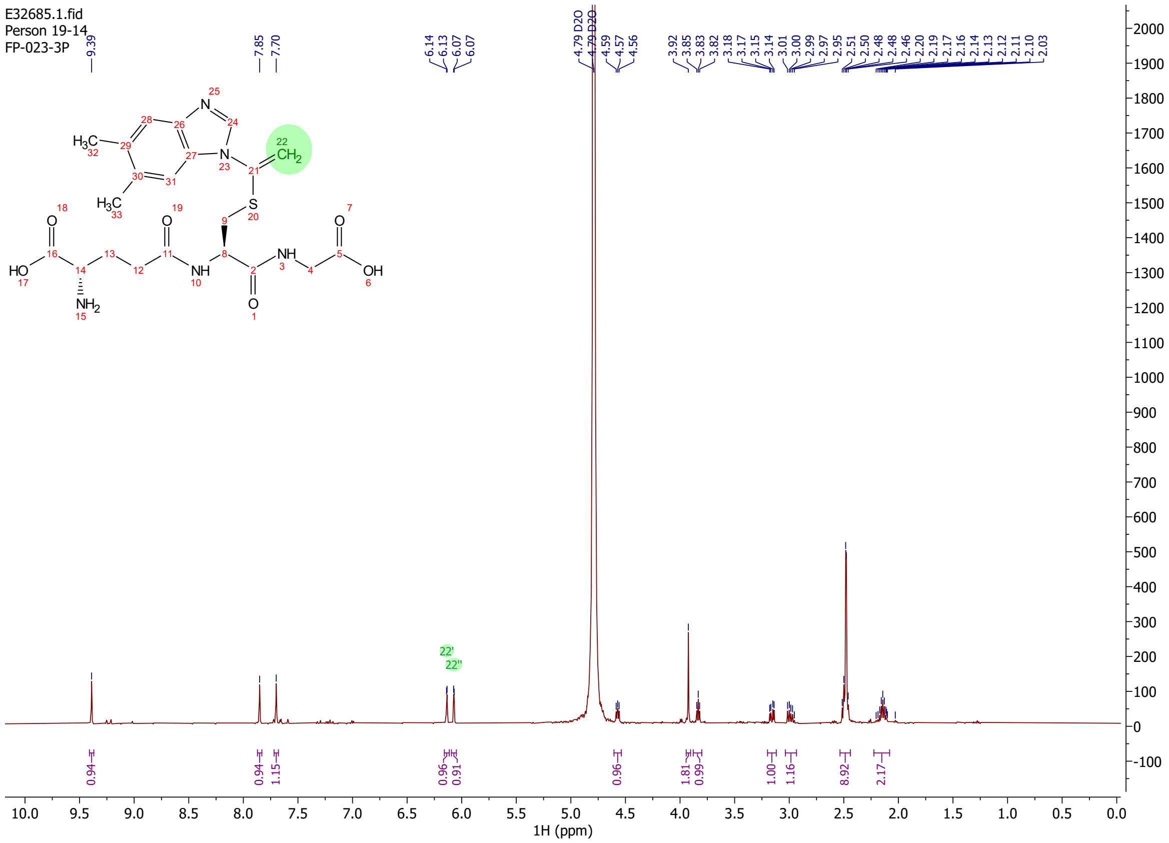

NMR spectra of compound **1b**

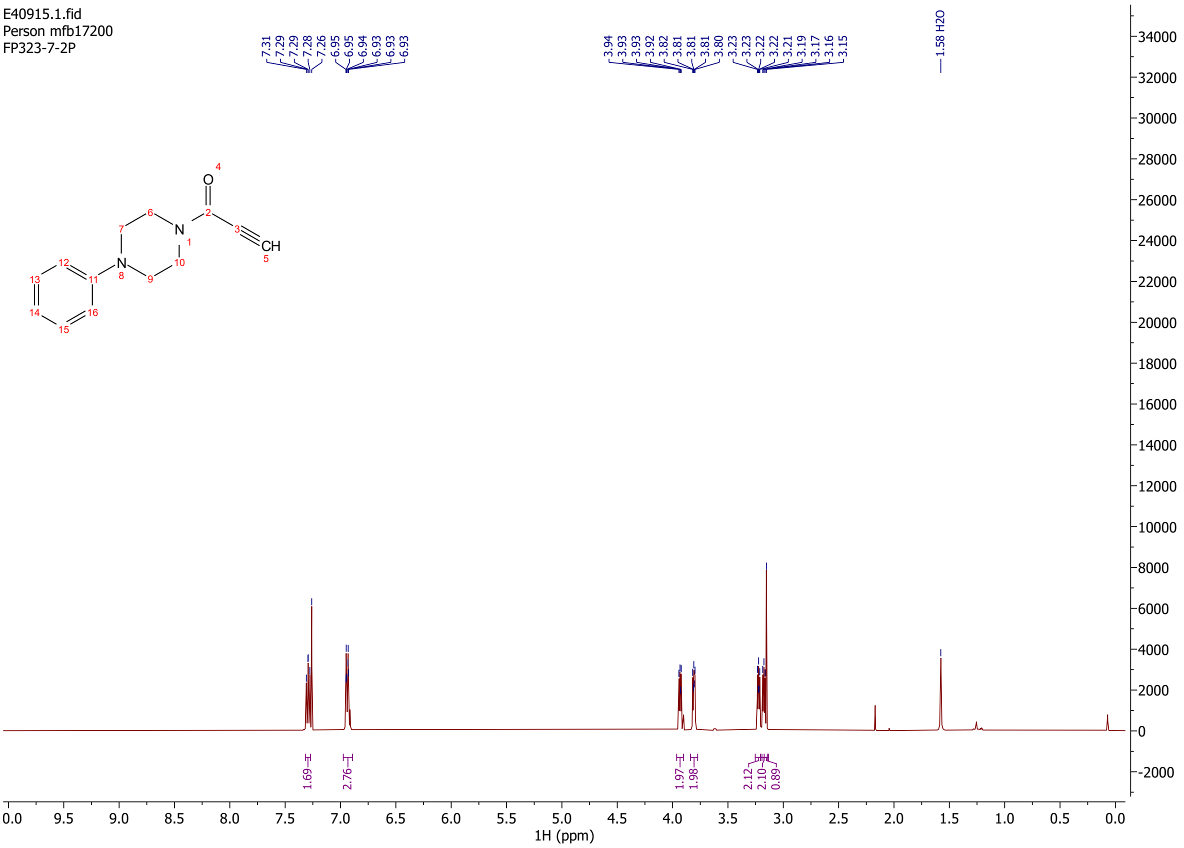

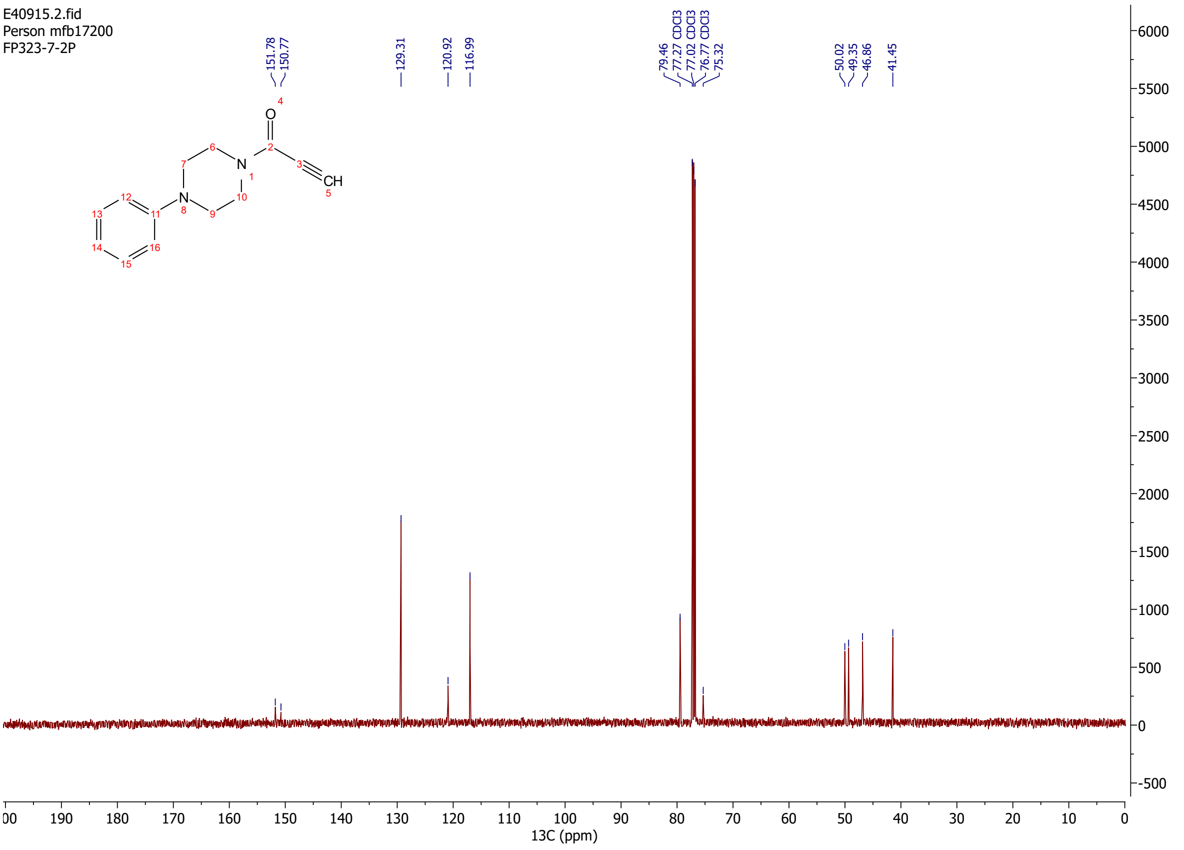

NMR spectra of compound **1c**

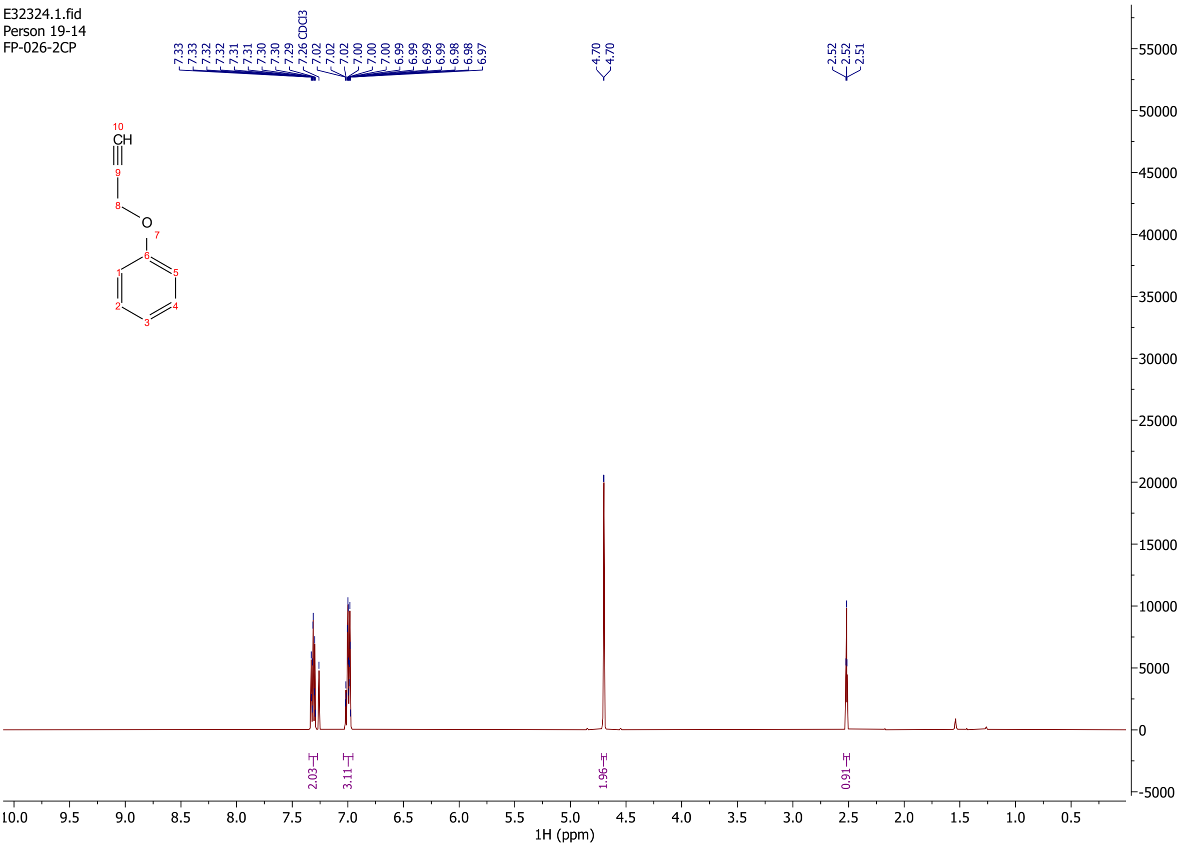

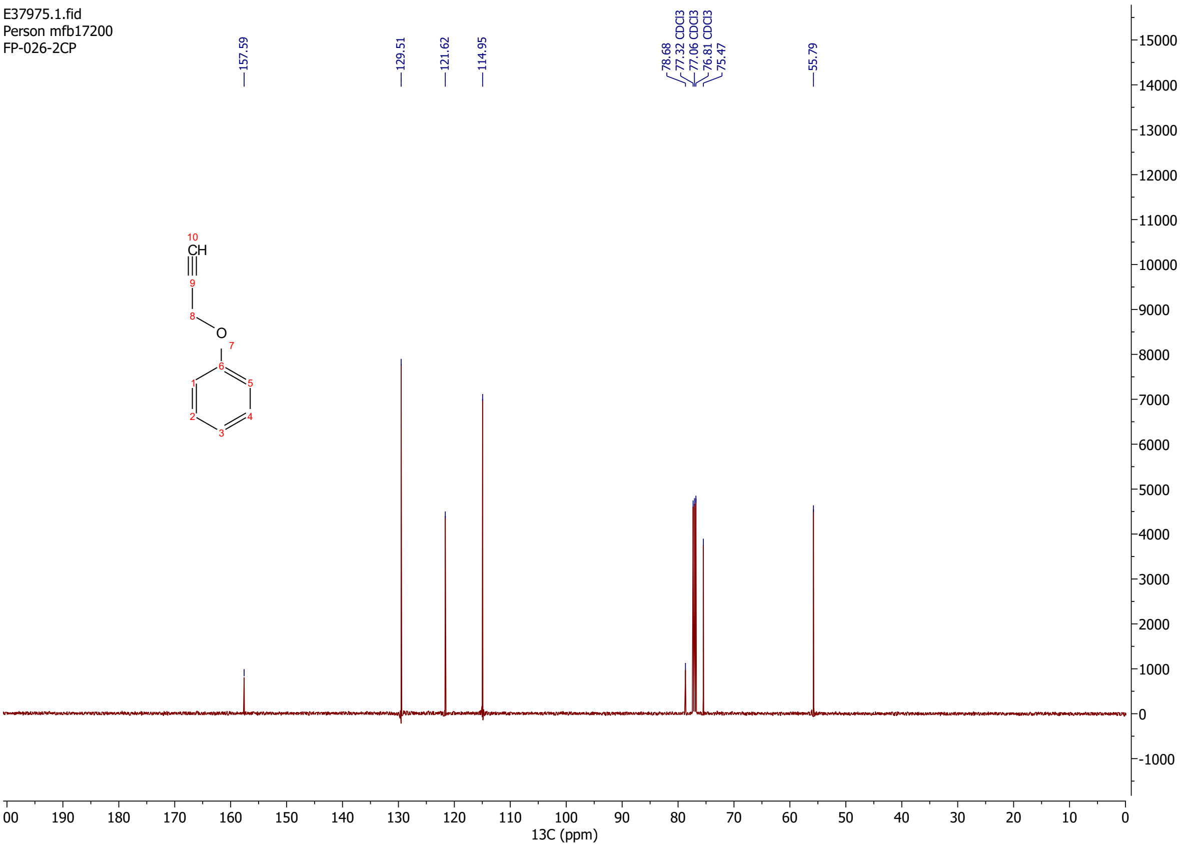

NMR spectra of compound **1d**

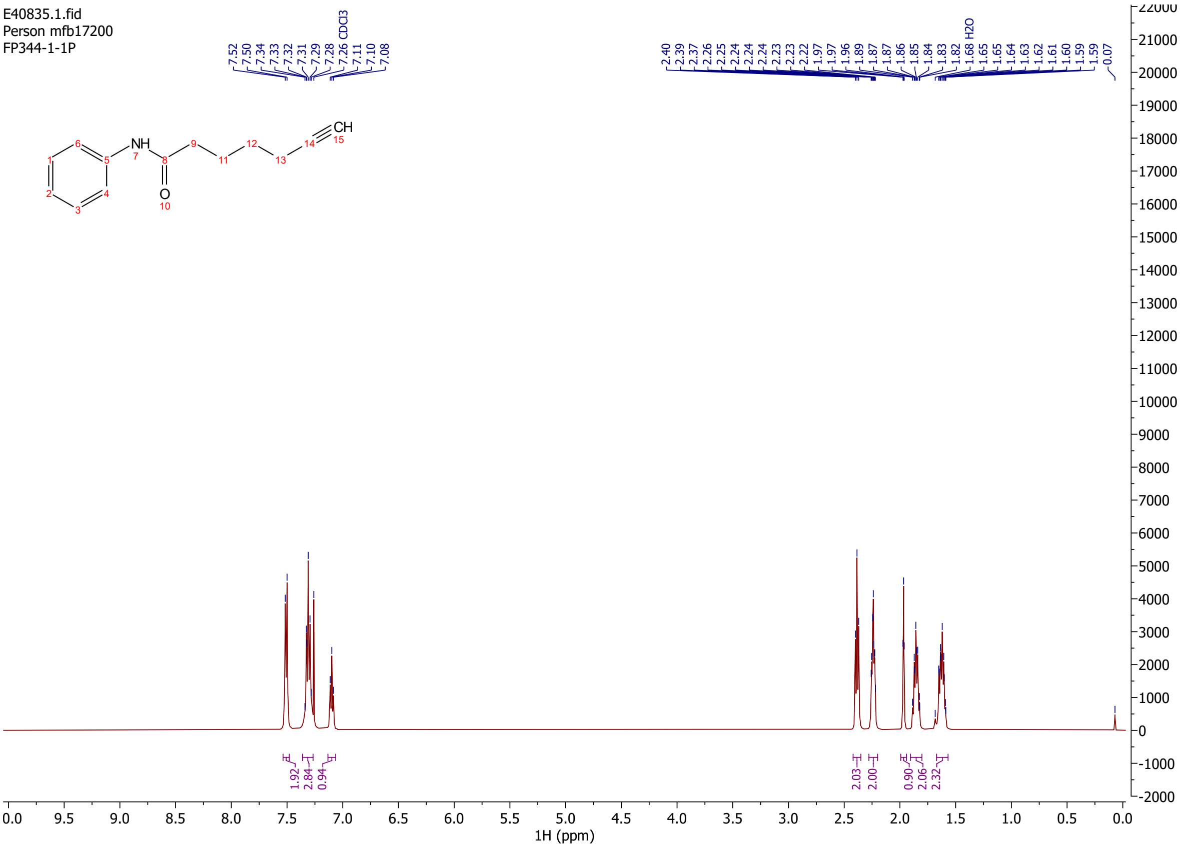

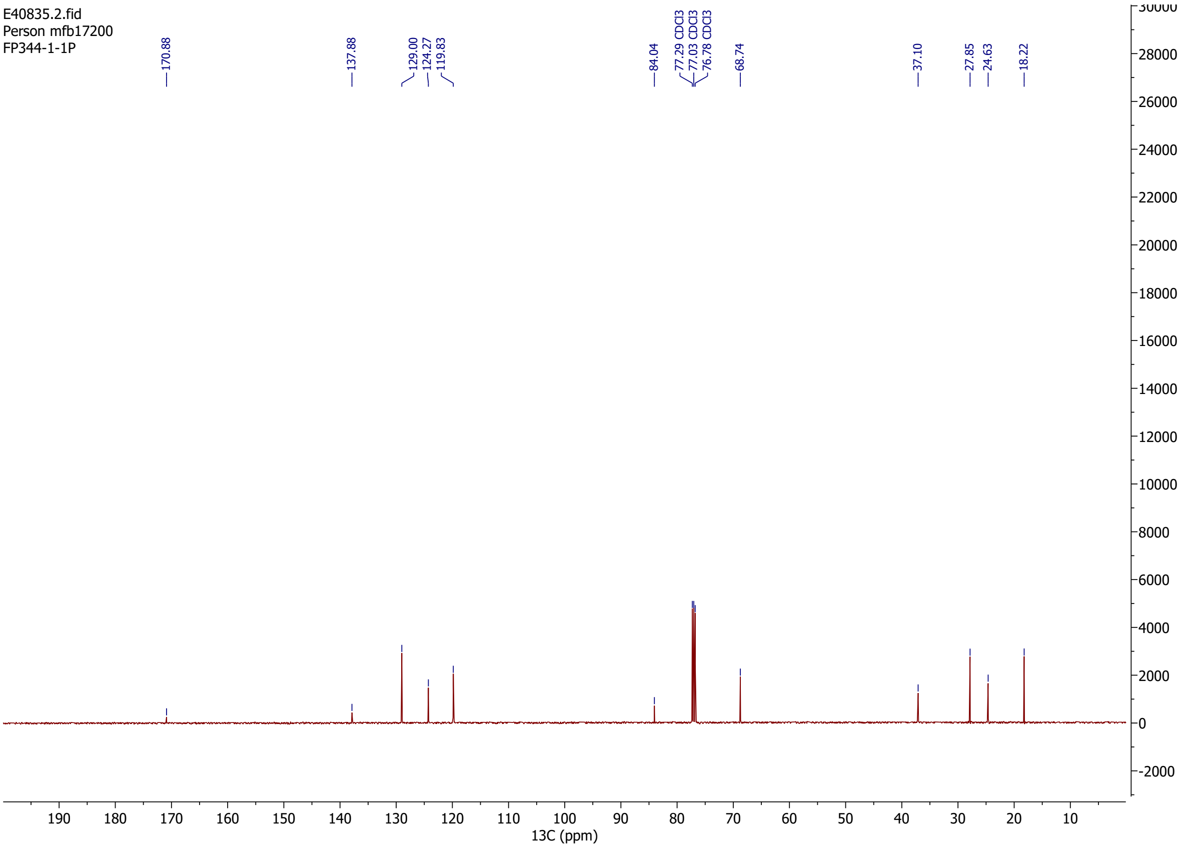

NMR spectra of compound **4b**

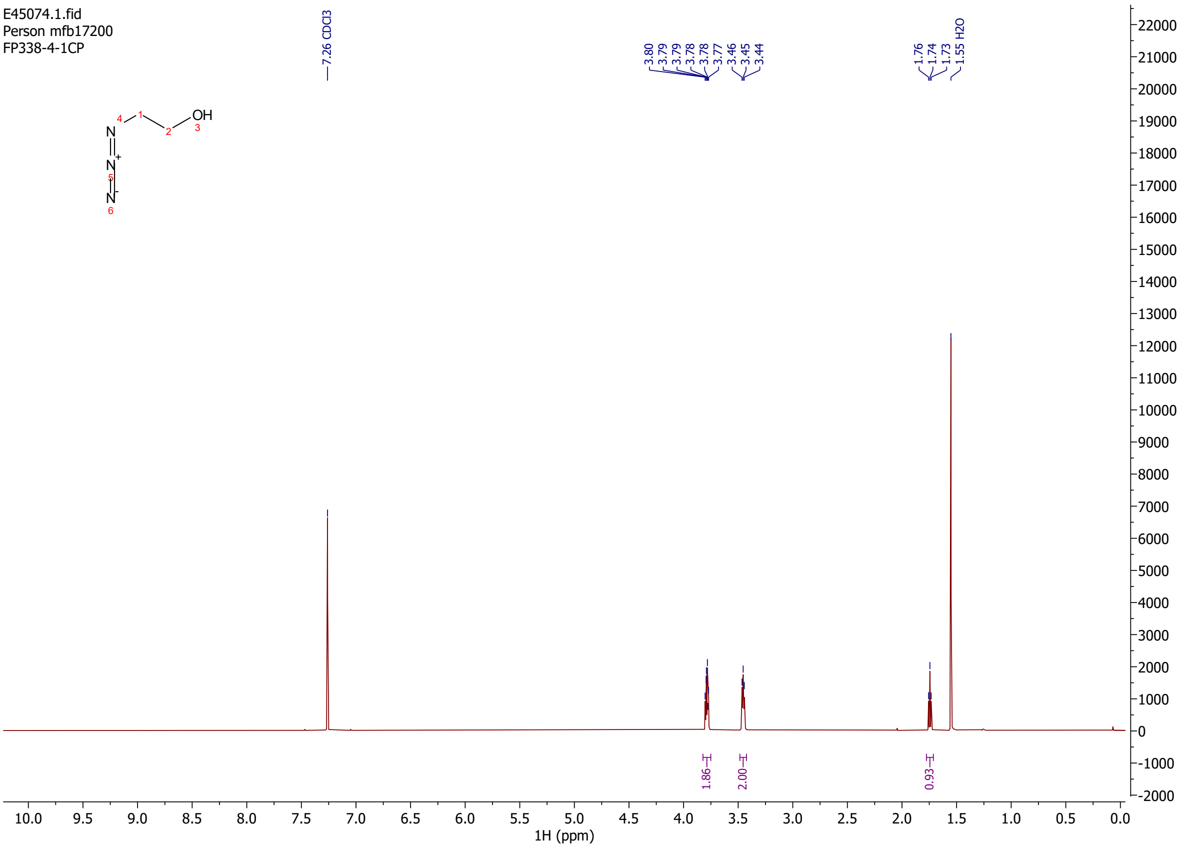

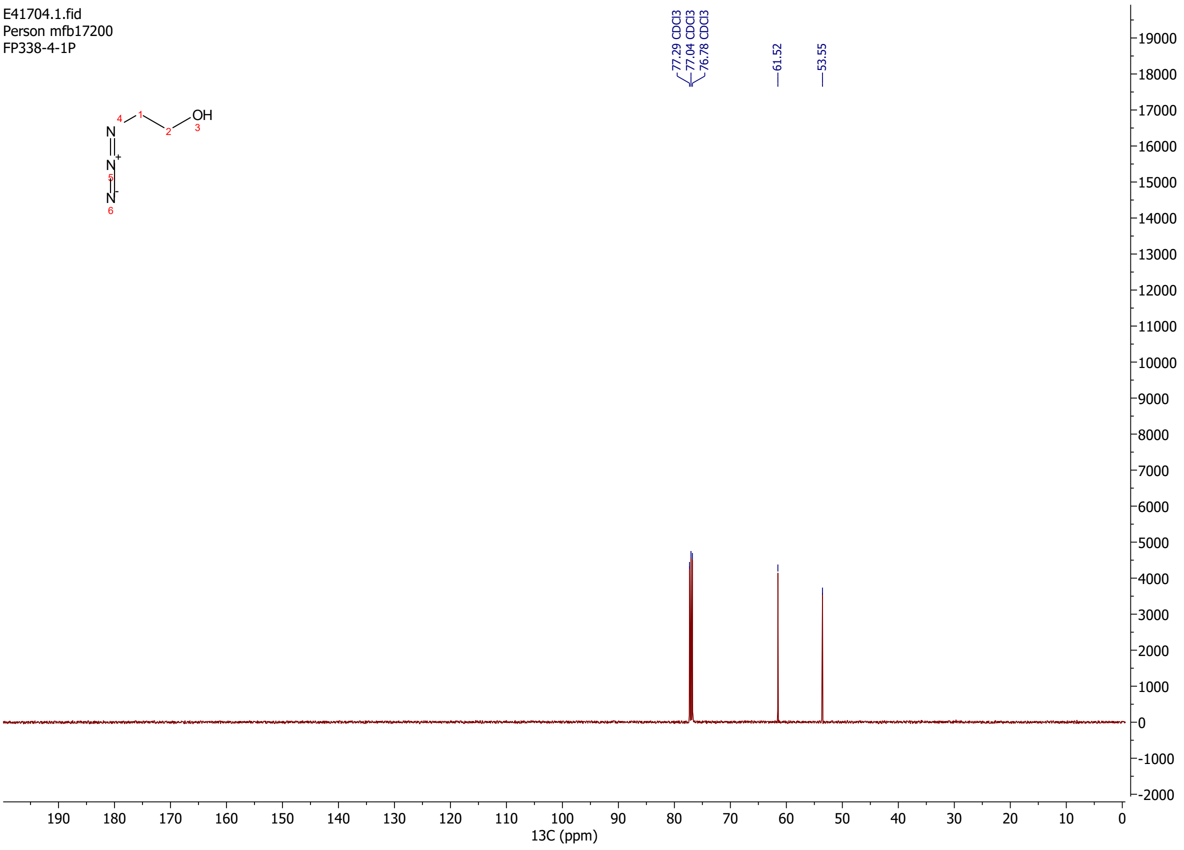

NMR spectra of compound **5a**

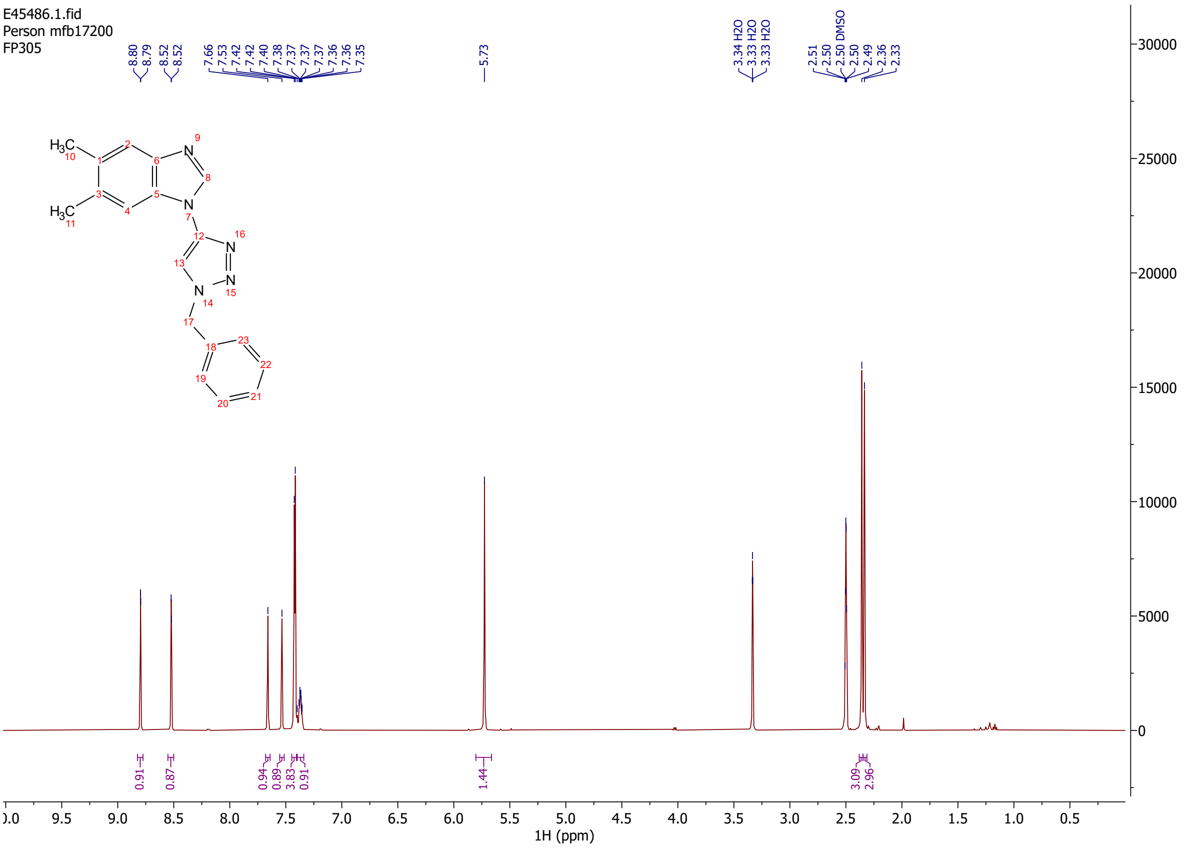

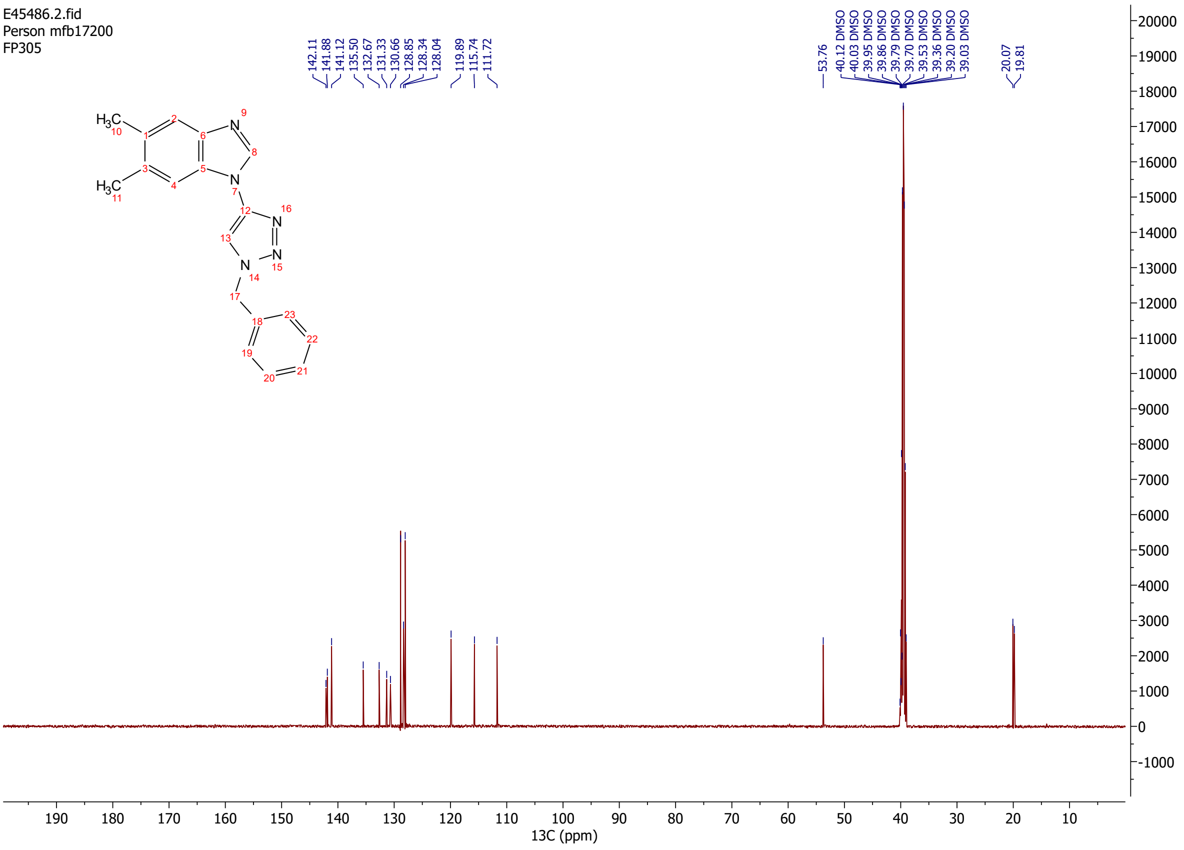

NMR spectra of compound **5b**

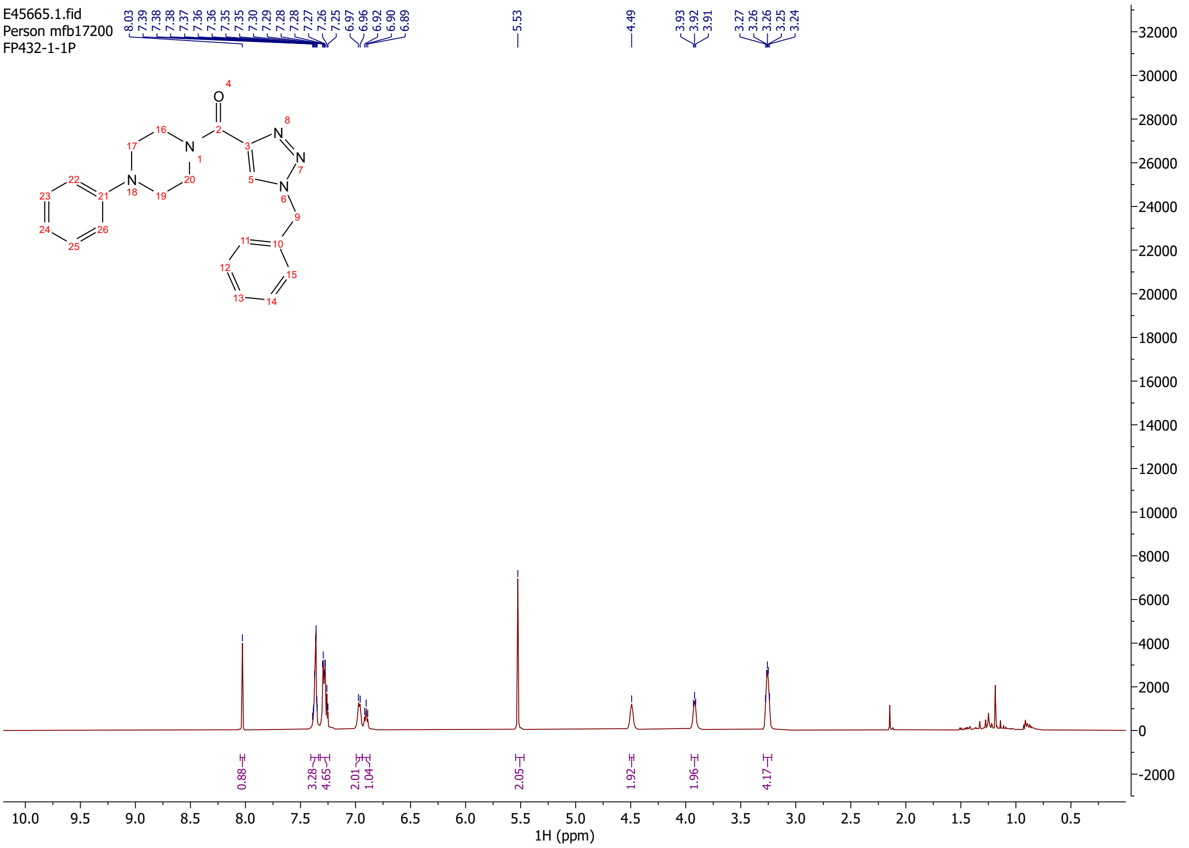

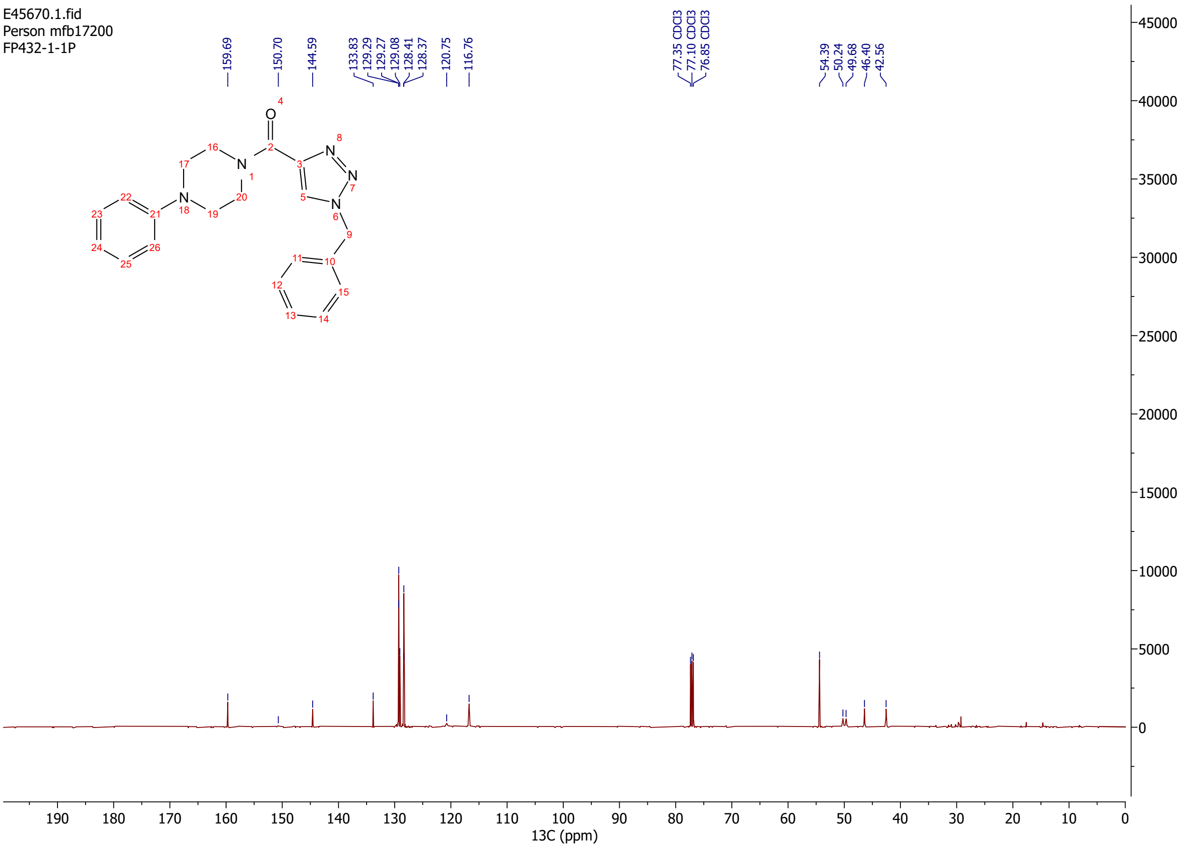

NMR spectra of compound **5c**

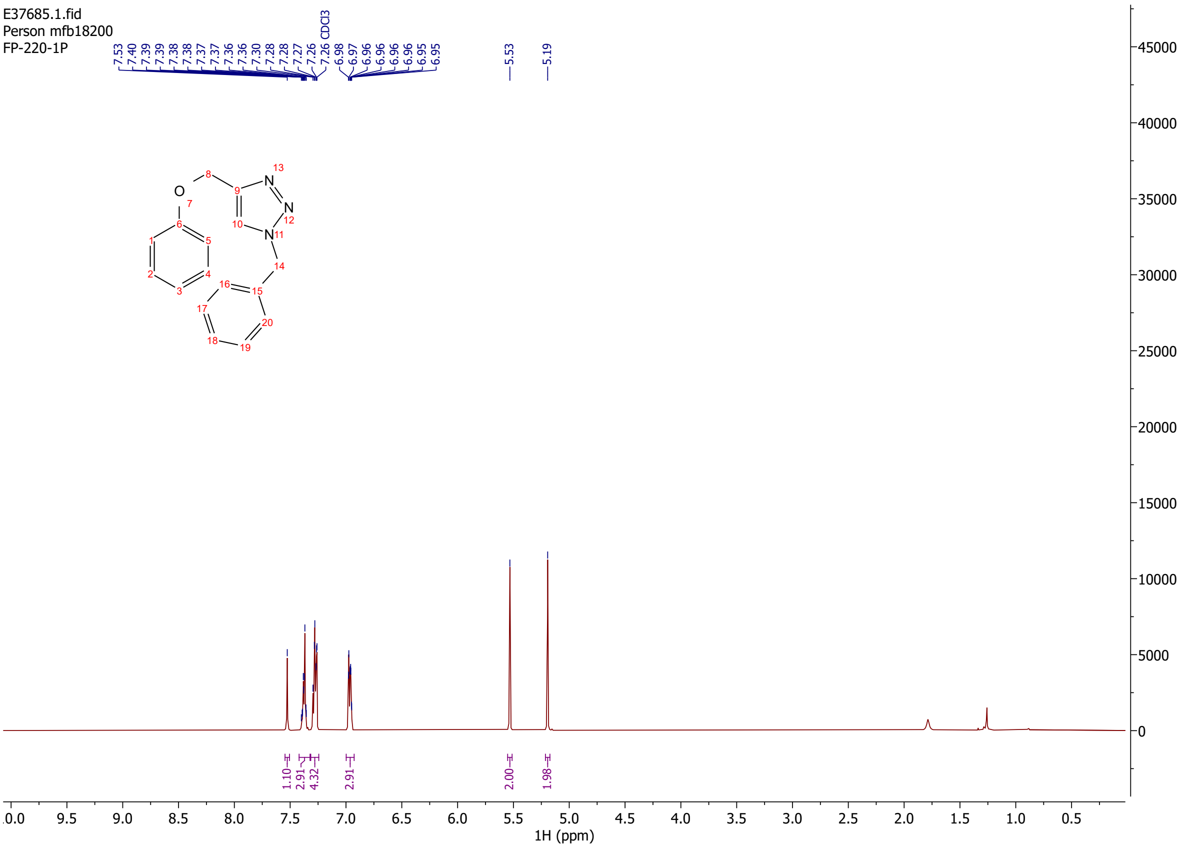

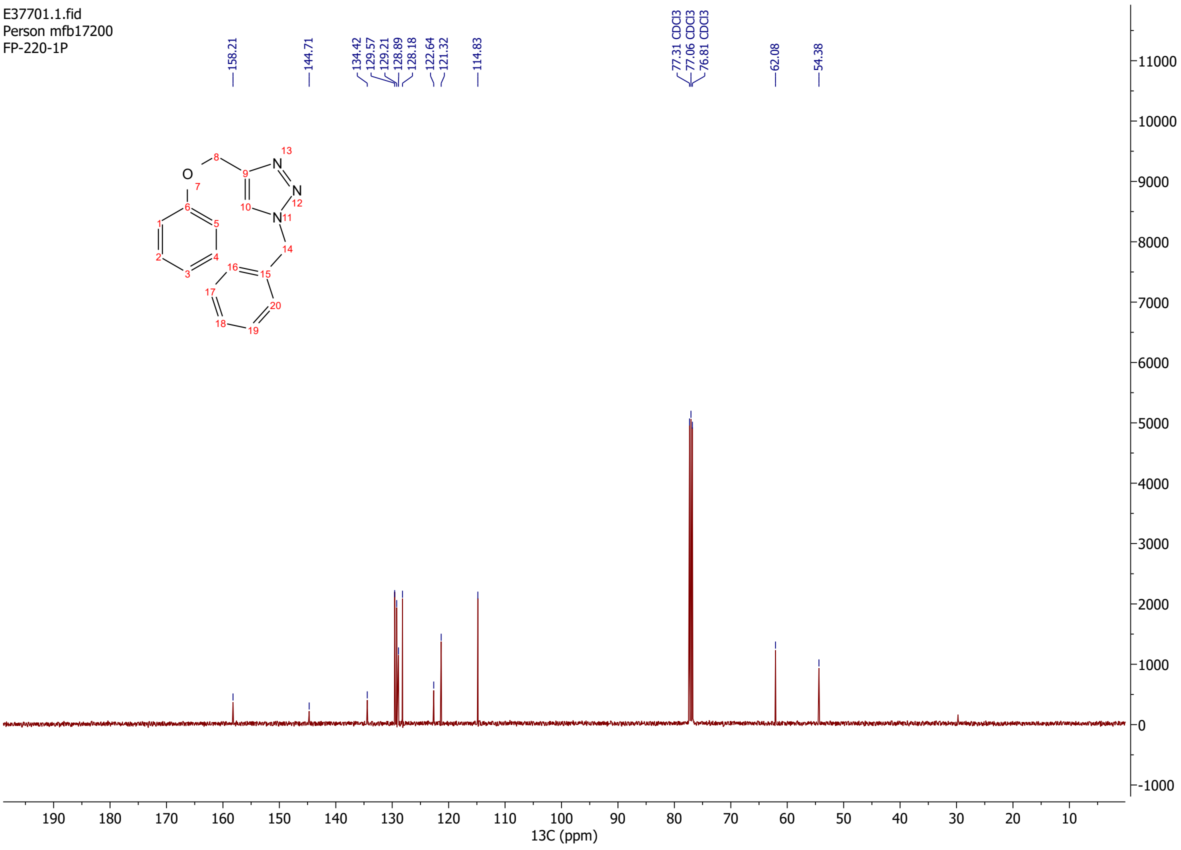

NMR spectra of compound **5d**

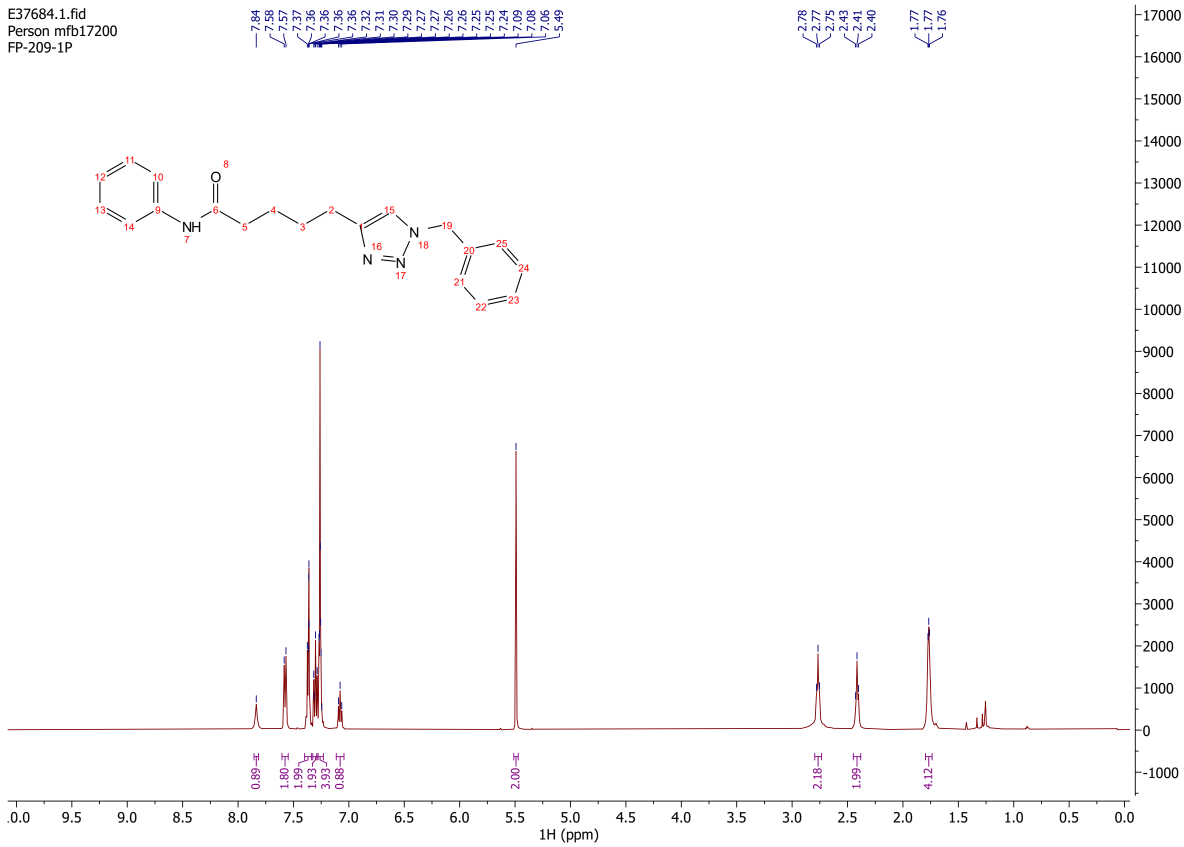

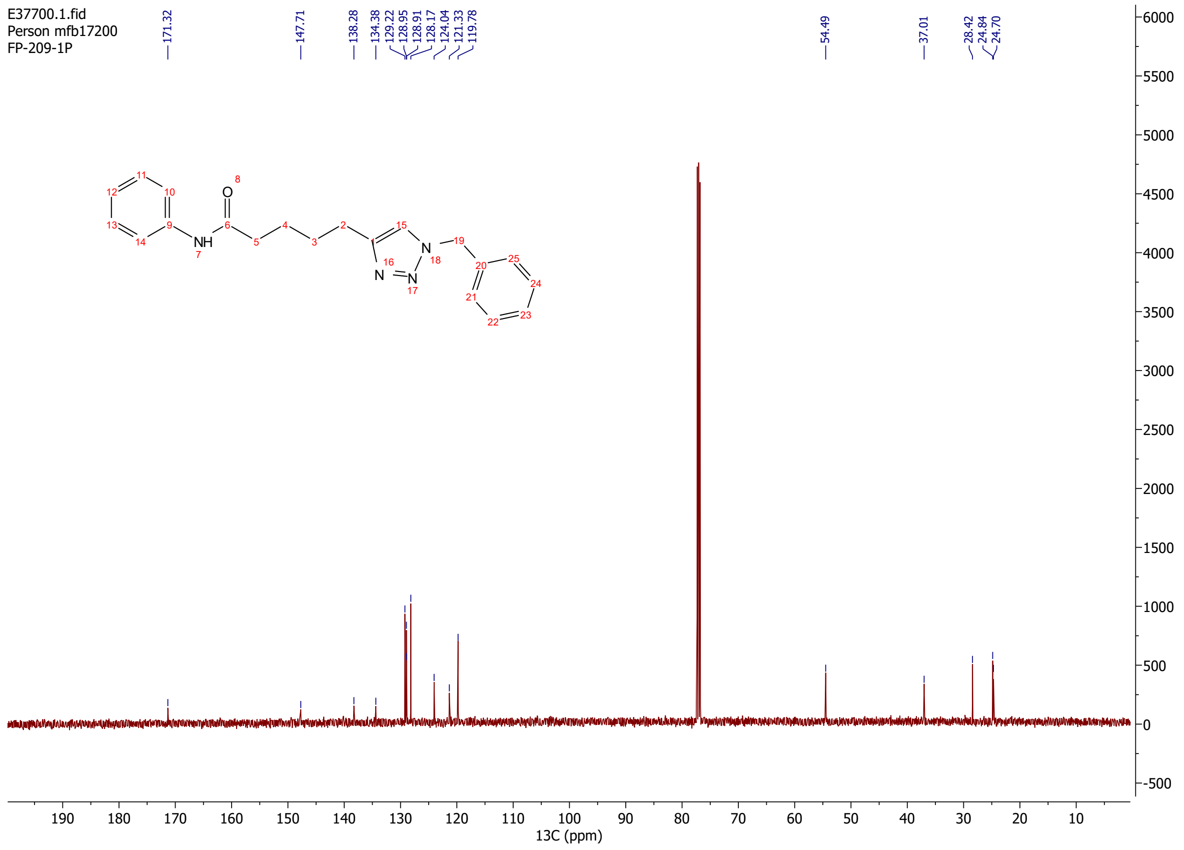

NMR spectra of compound **5e**

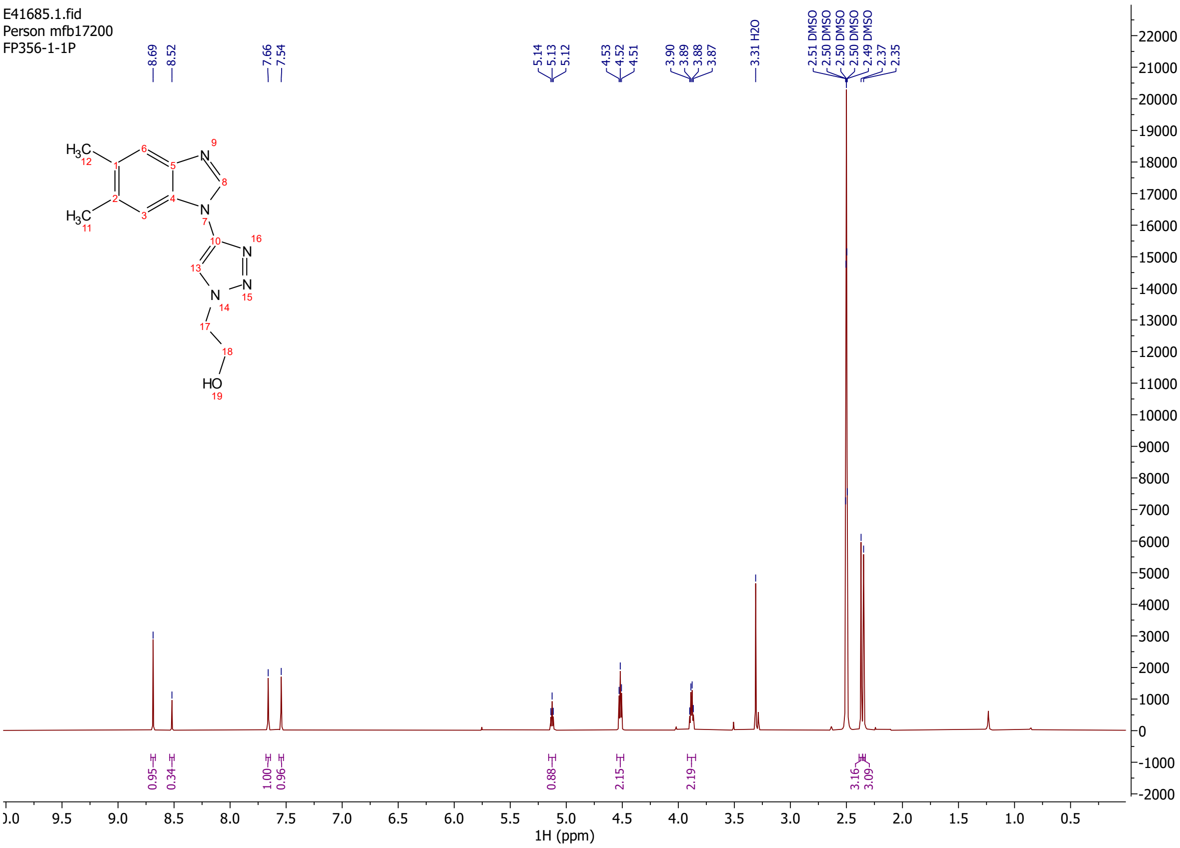

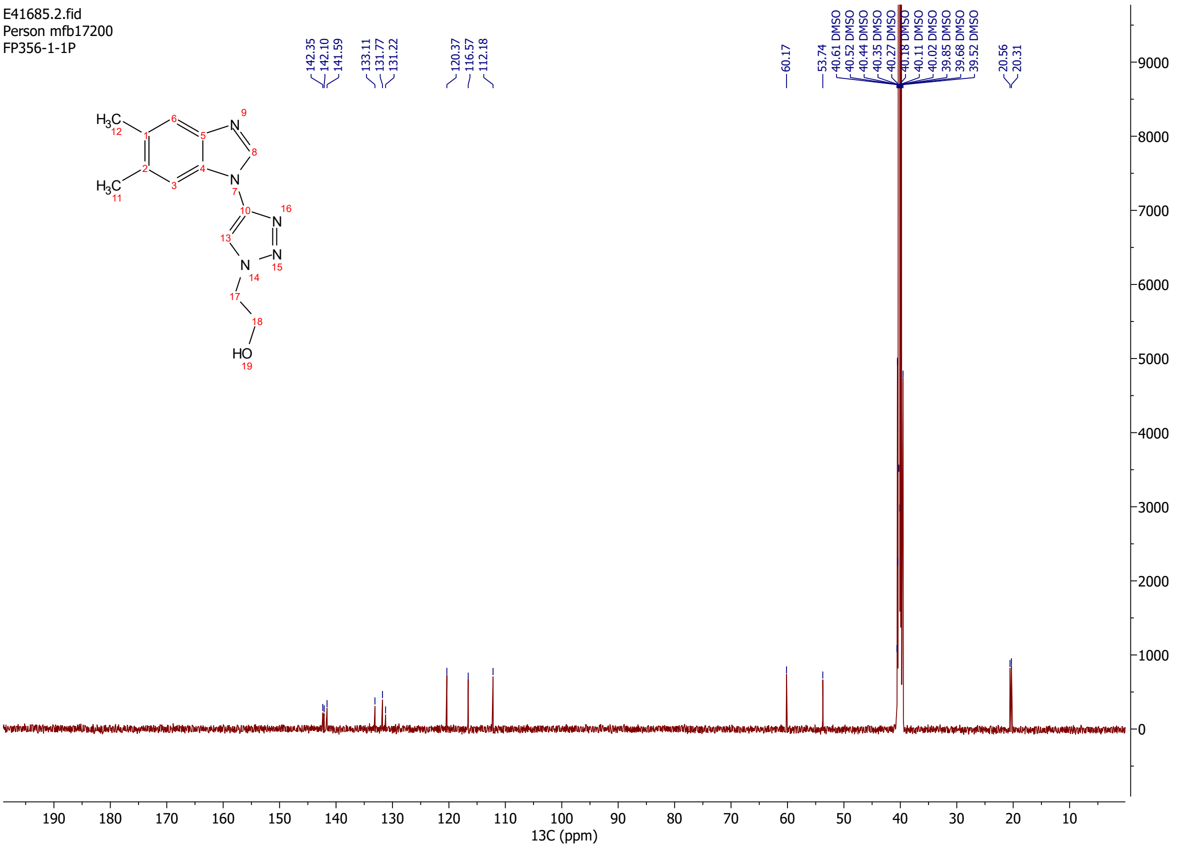

NMR spectra of compound **5f**

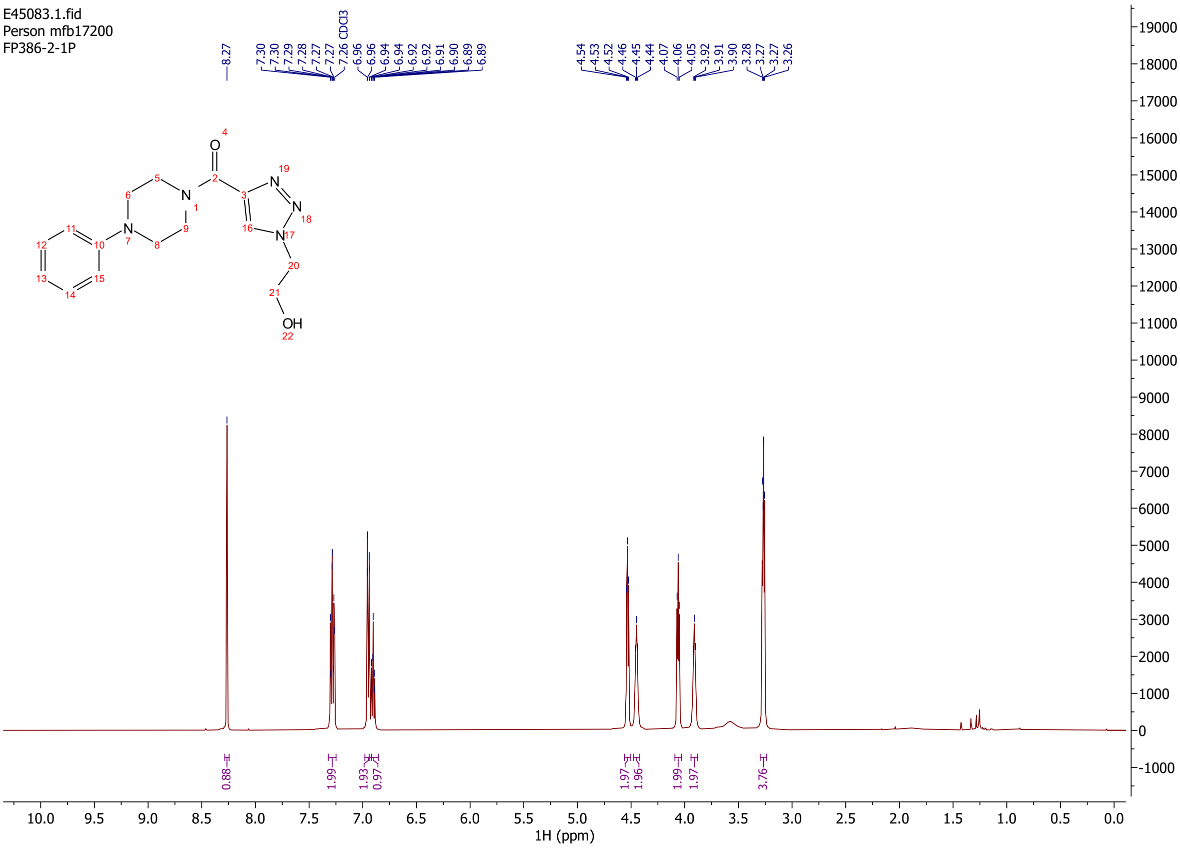

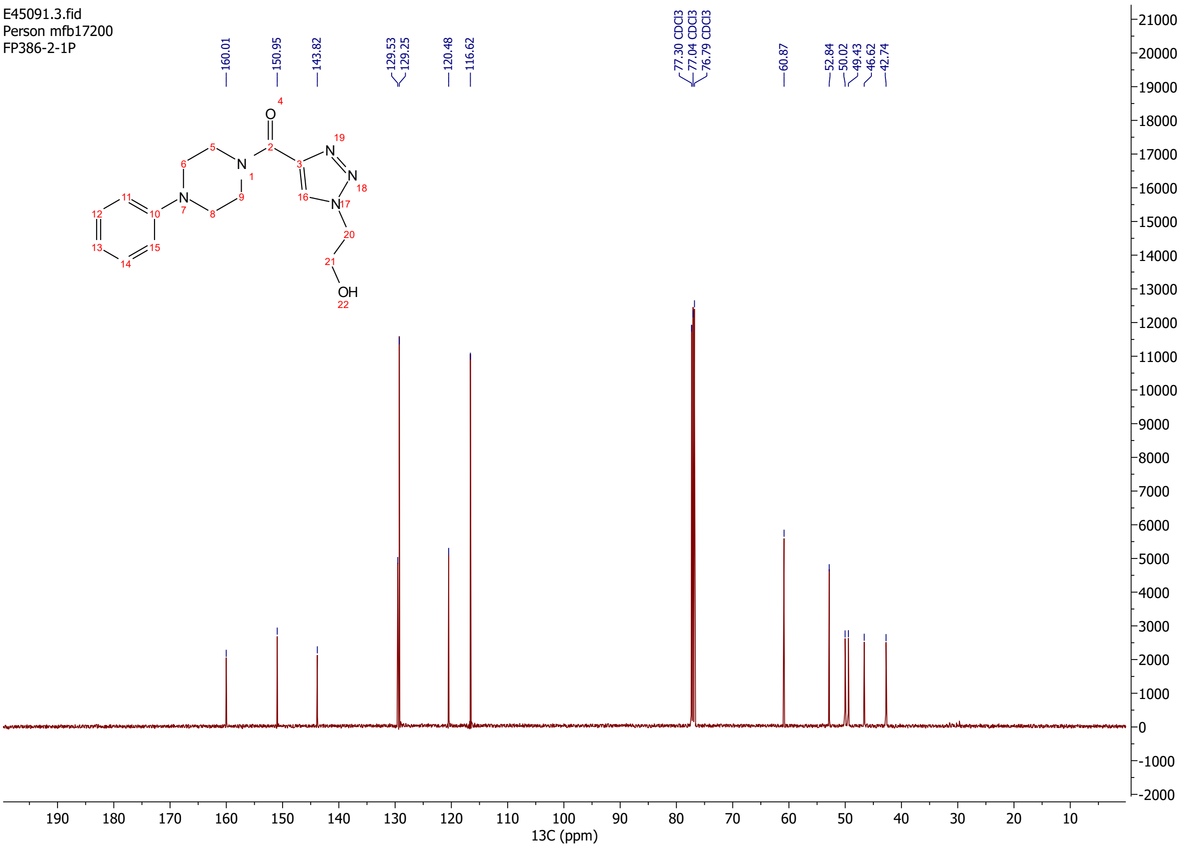

NMR spectra of compound **5g**

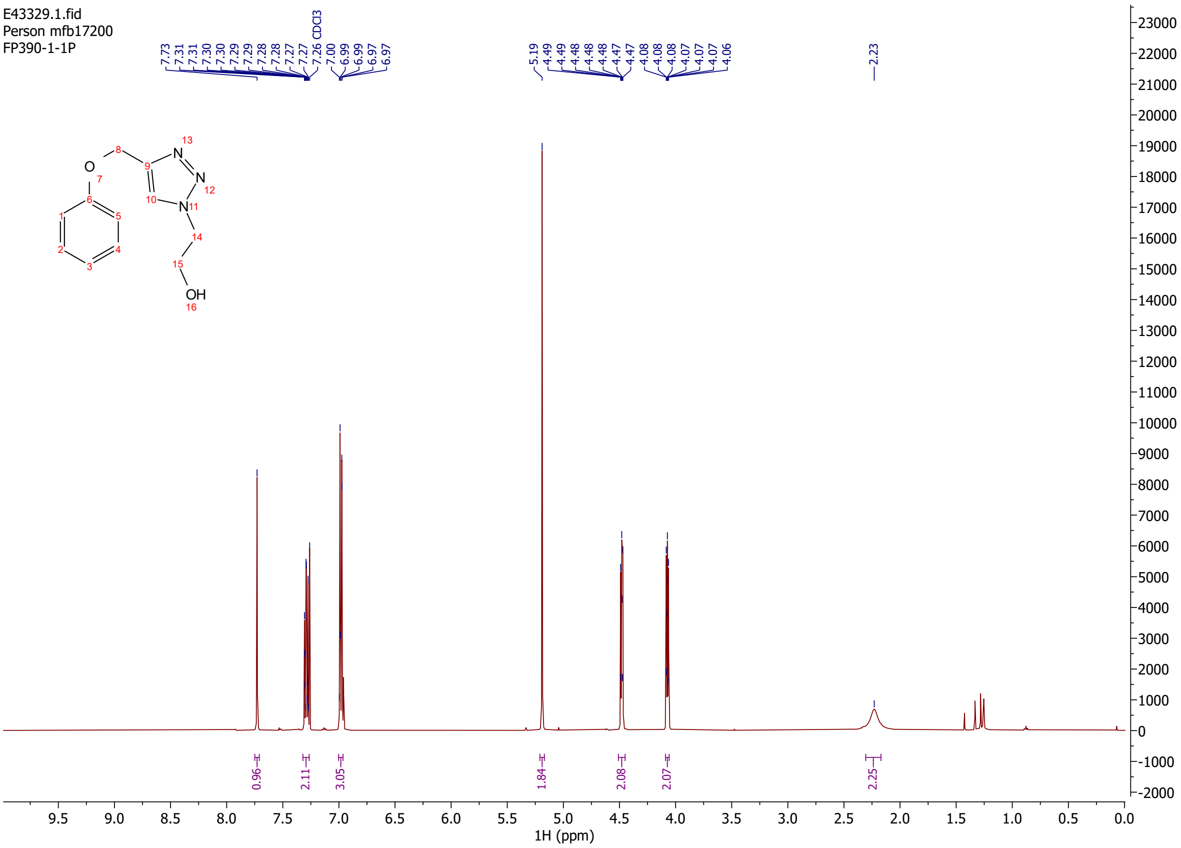

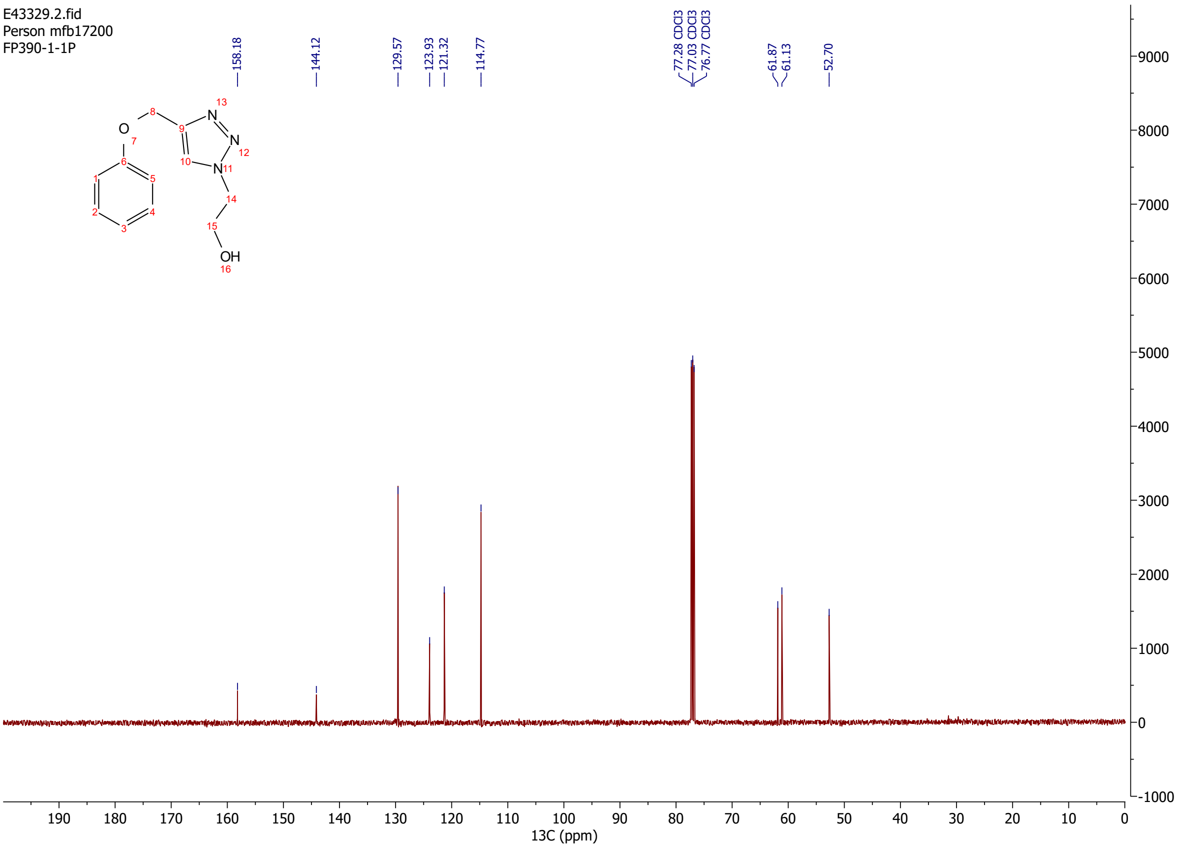

NMR spectra of compound **5h**

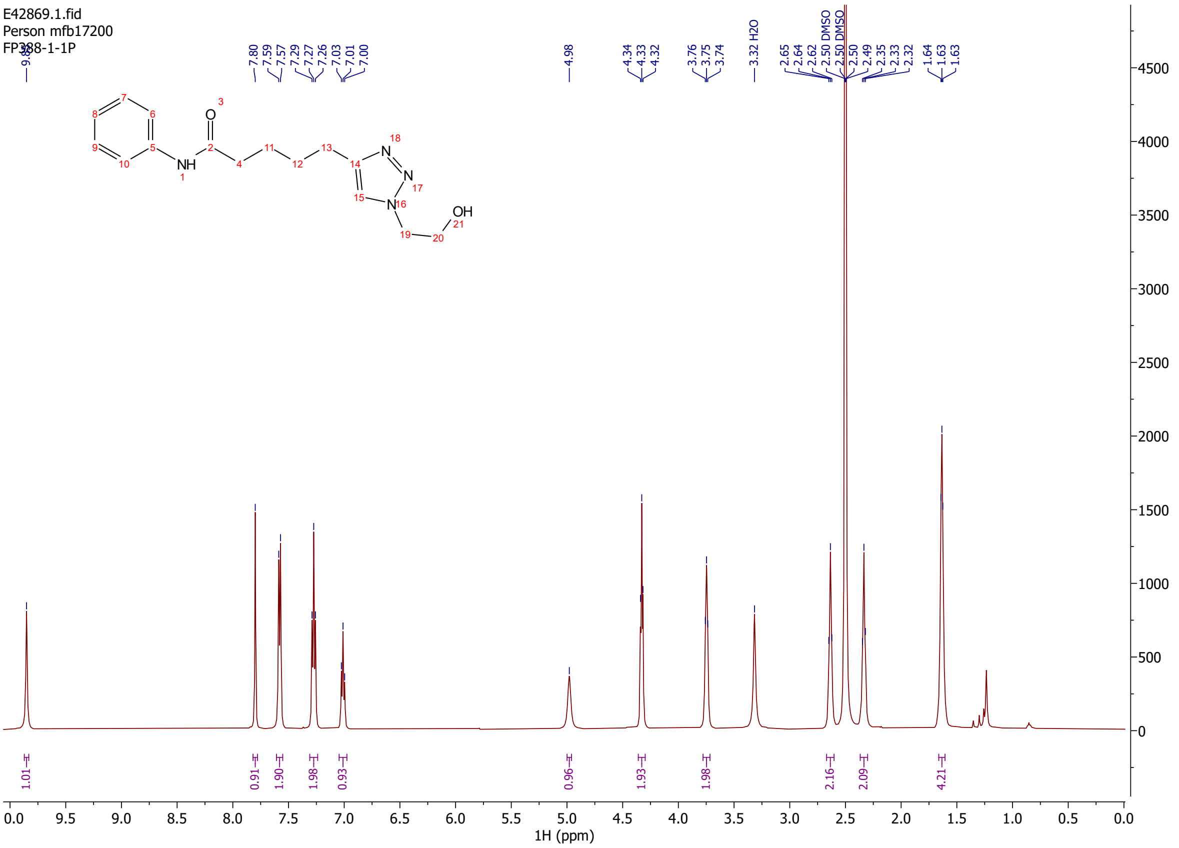

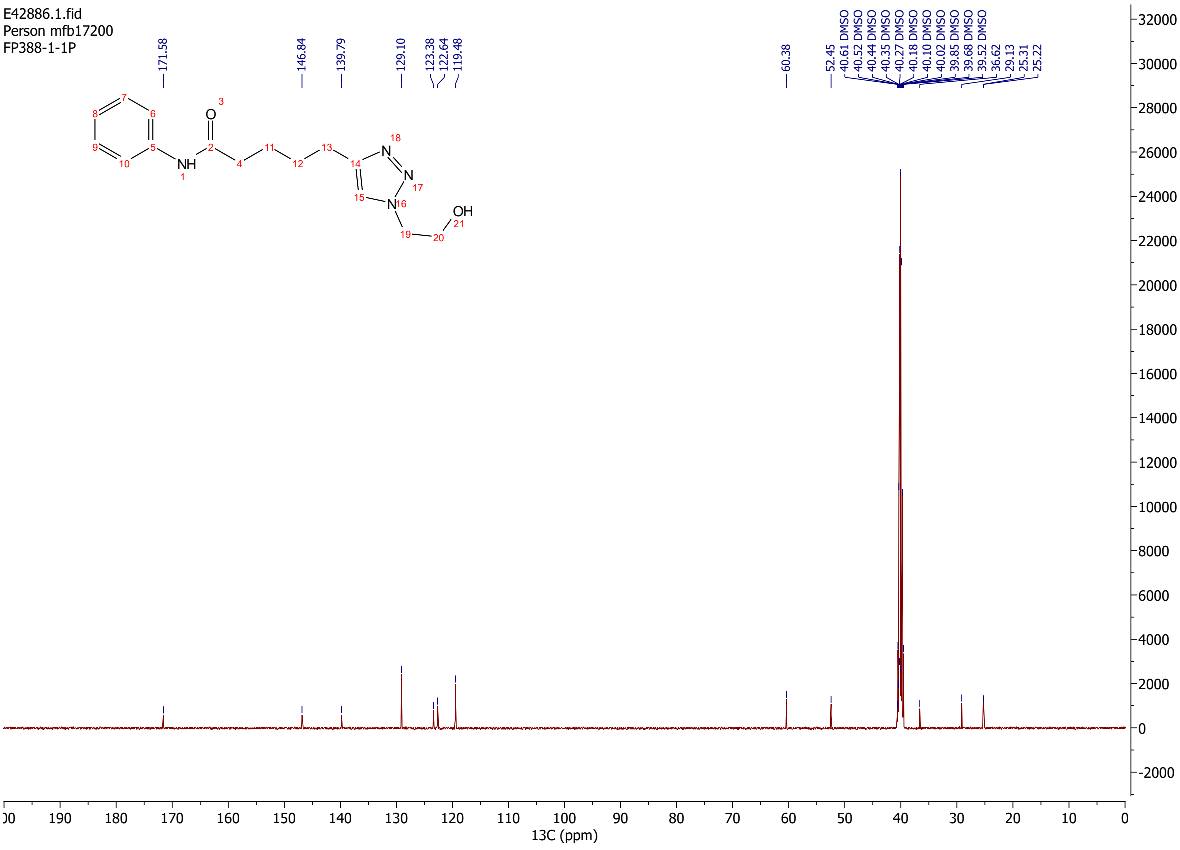

NMR spectra of compound **5i**

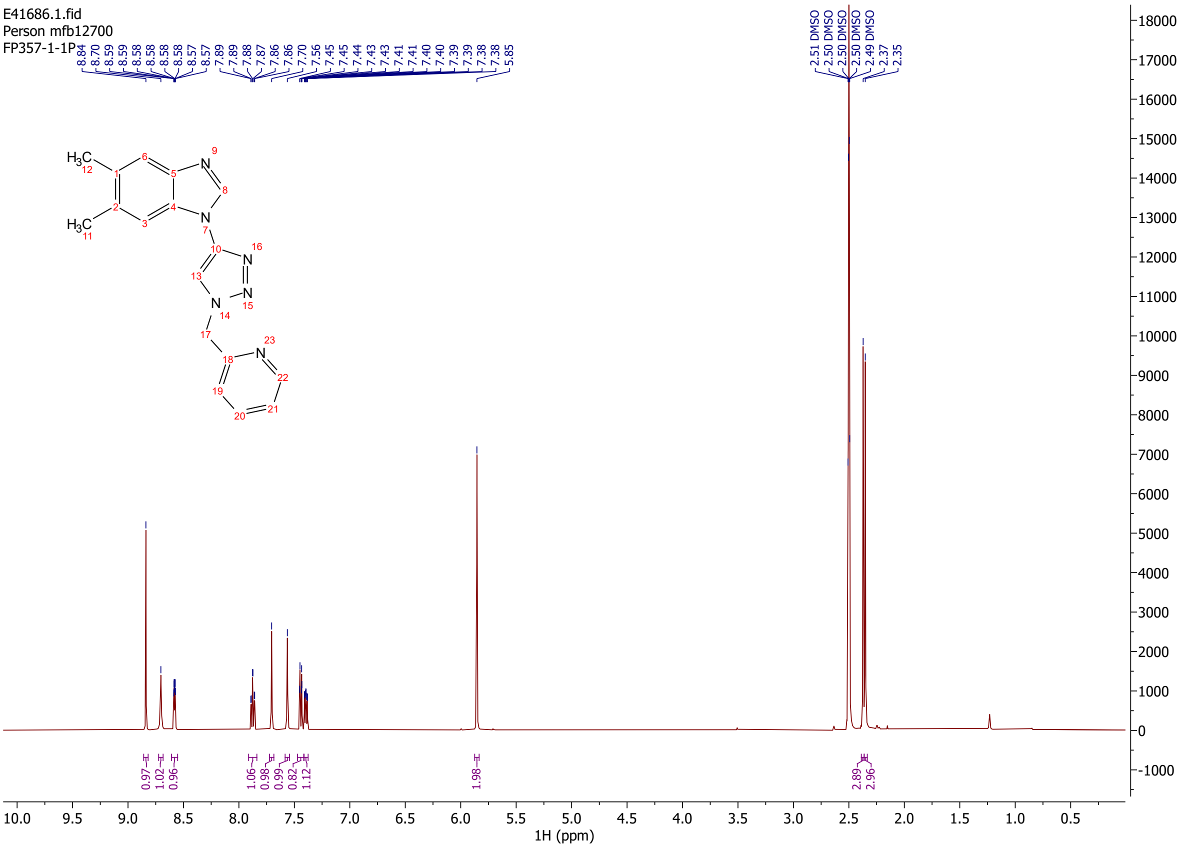

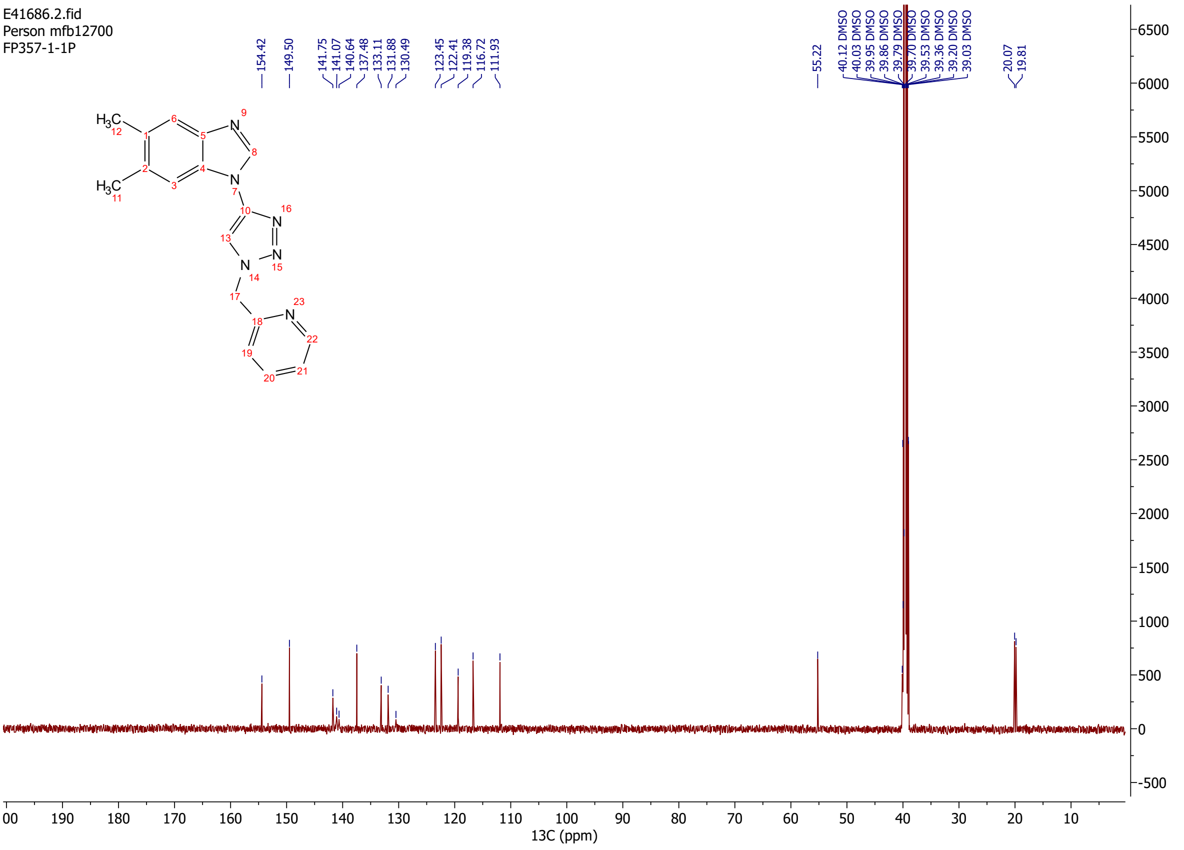

NMR spectra of compound **5j**

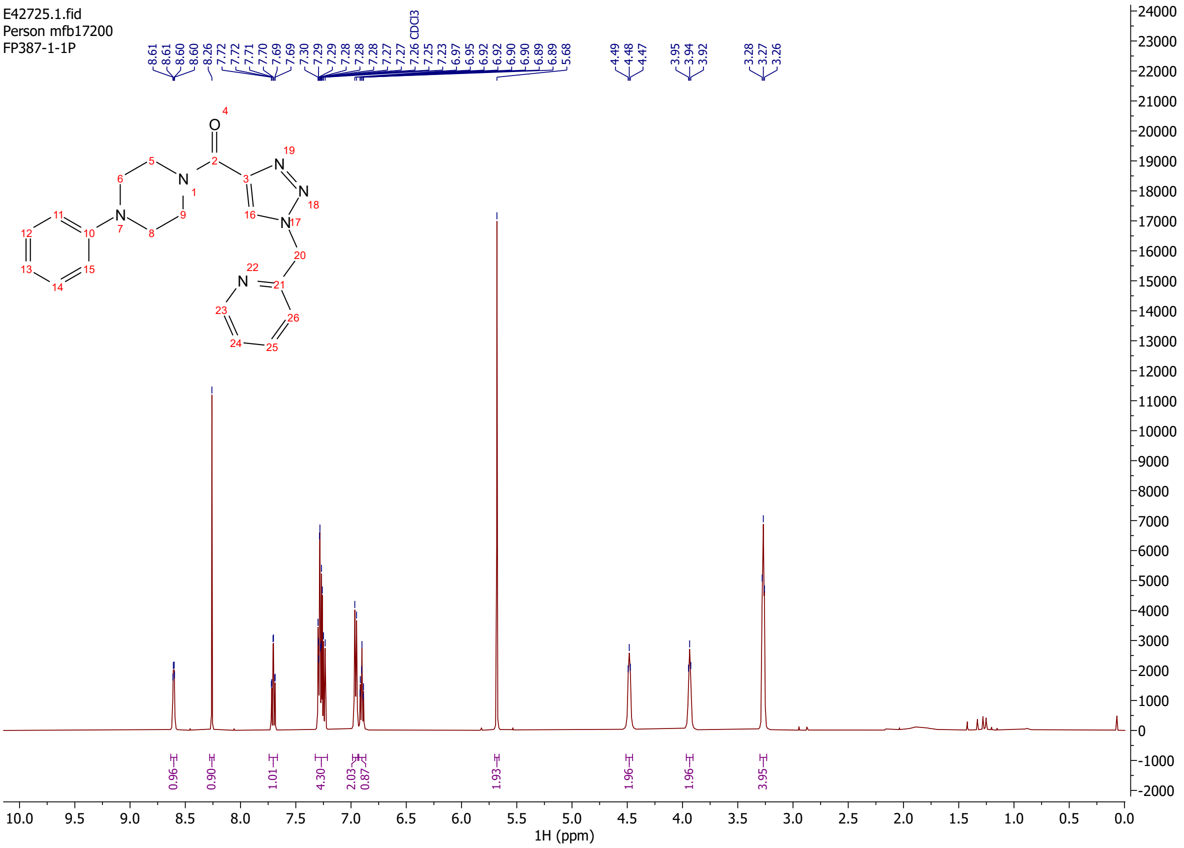

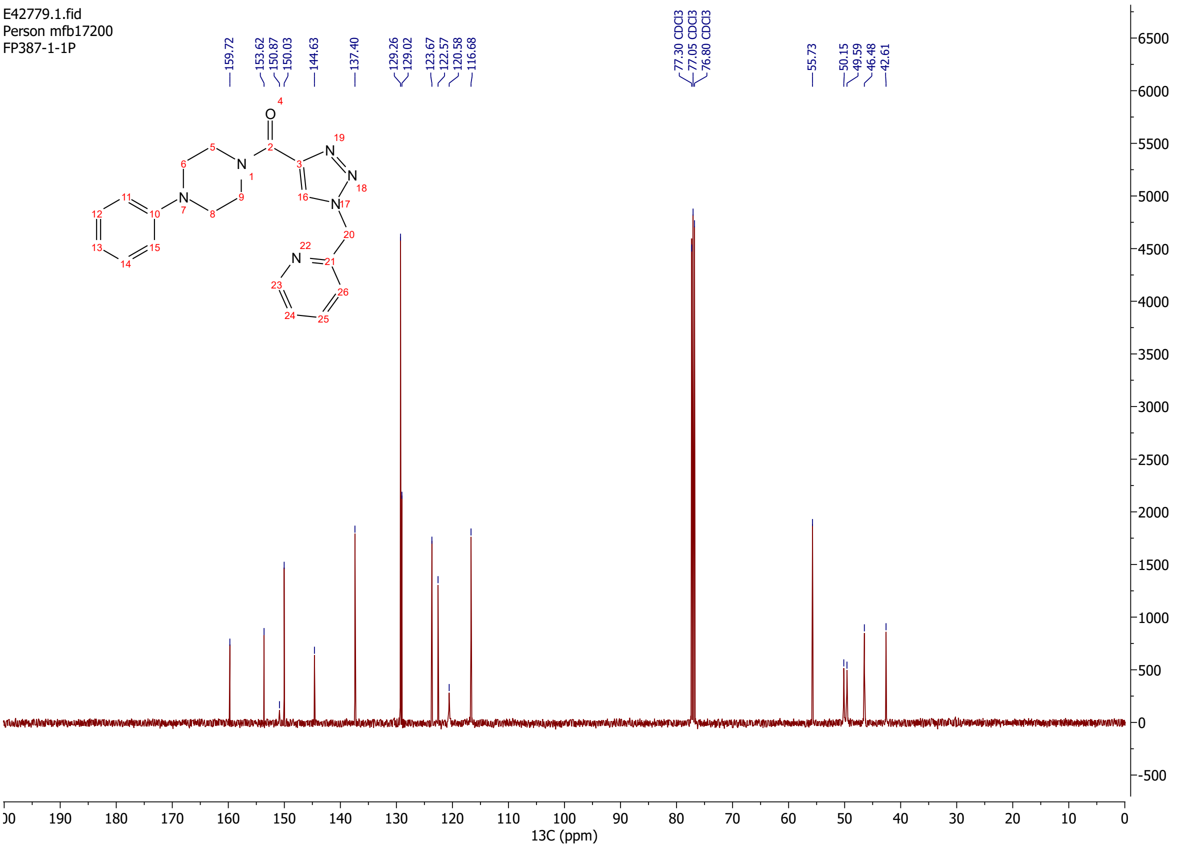

NMR spectra for compound **5k**

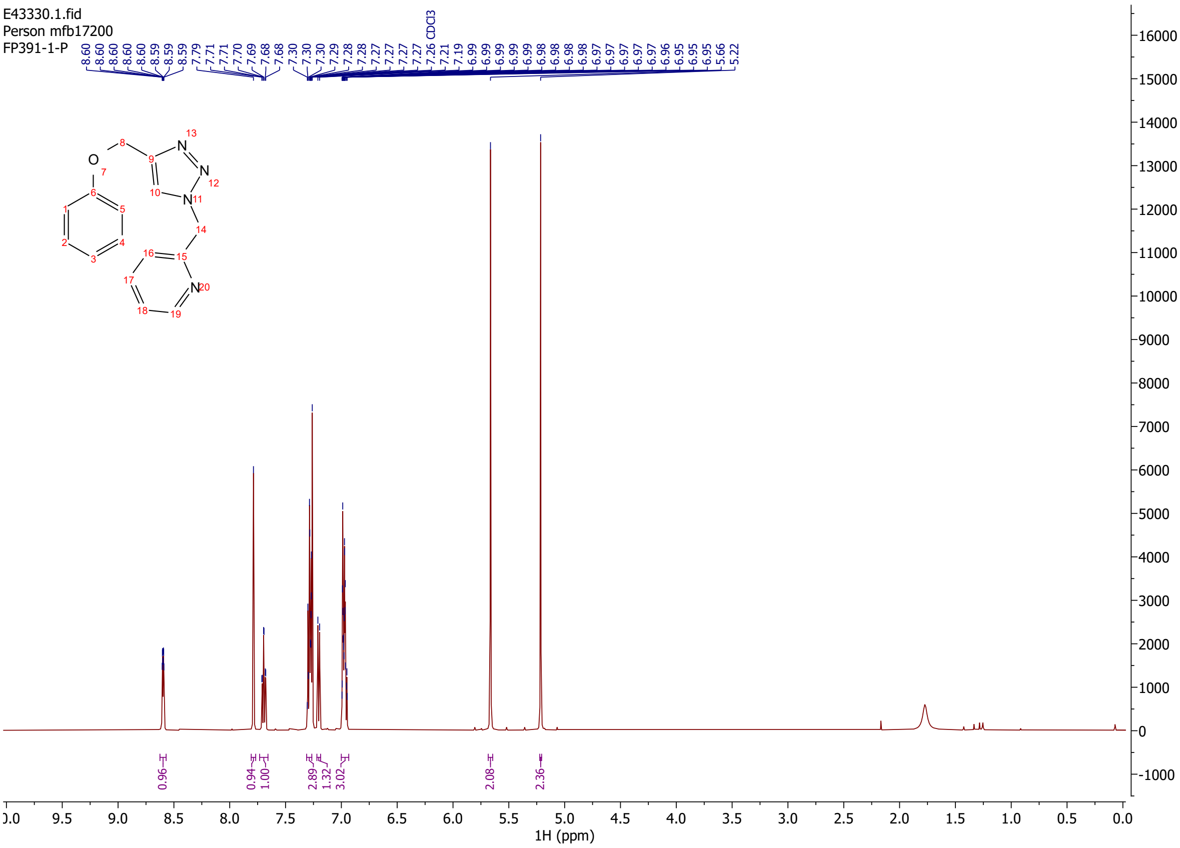

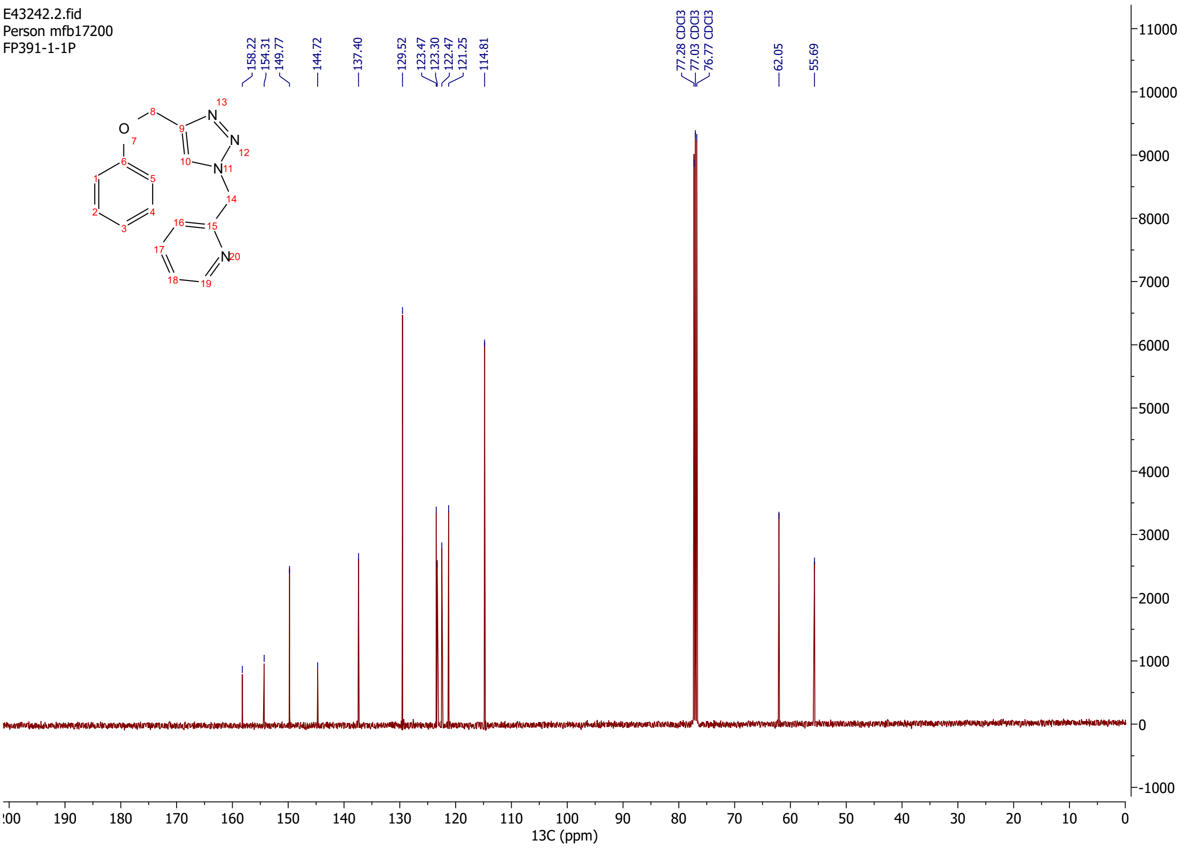

NMR spectra for compound **5l**

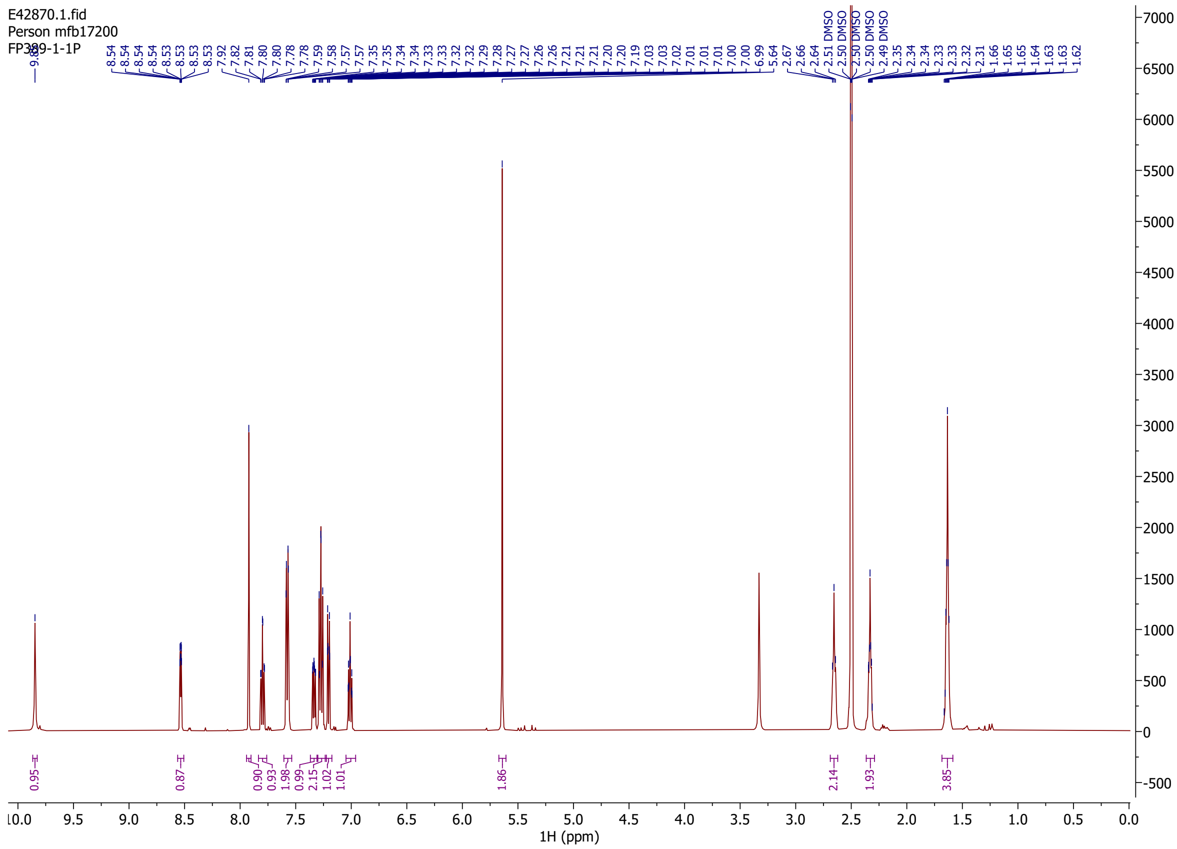

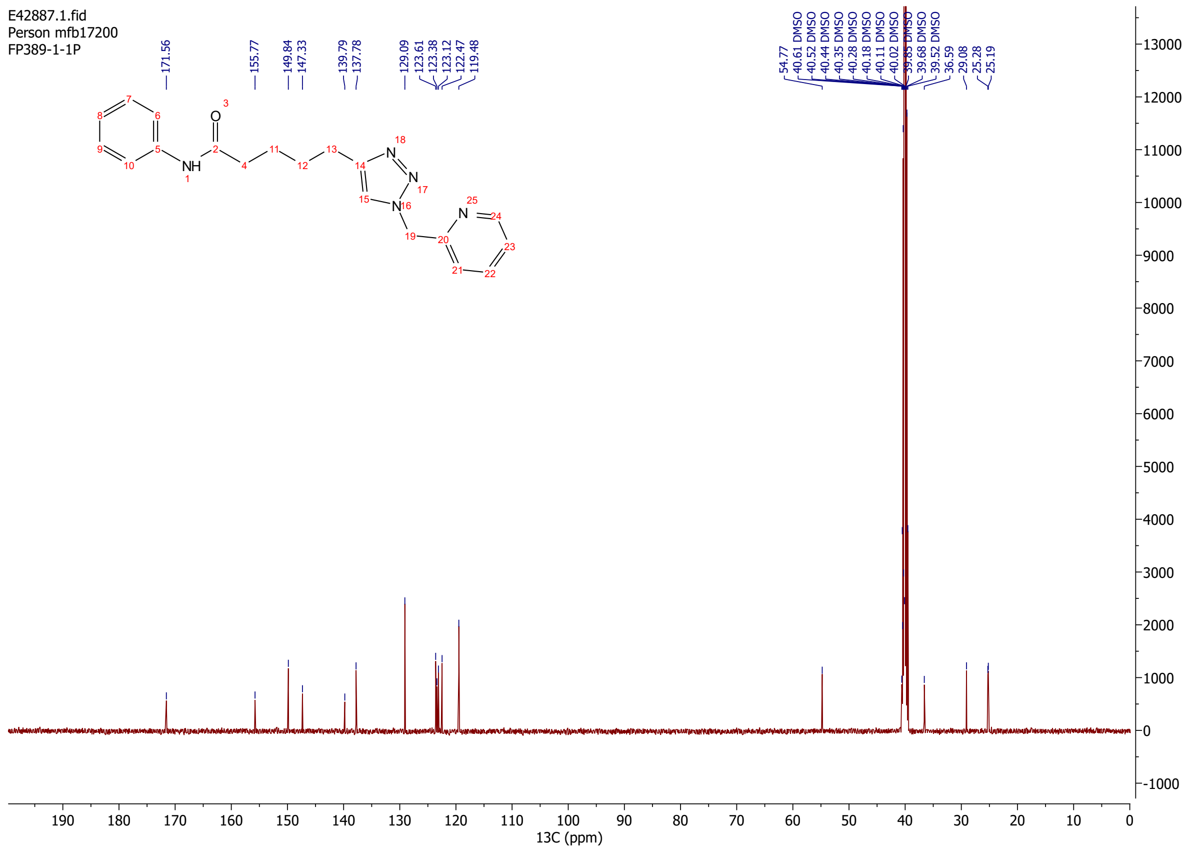

NMR spectra for compound **S2**

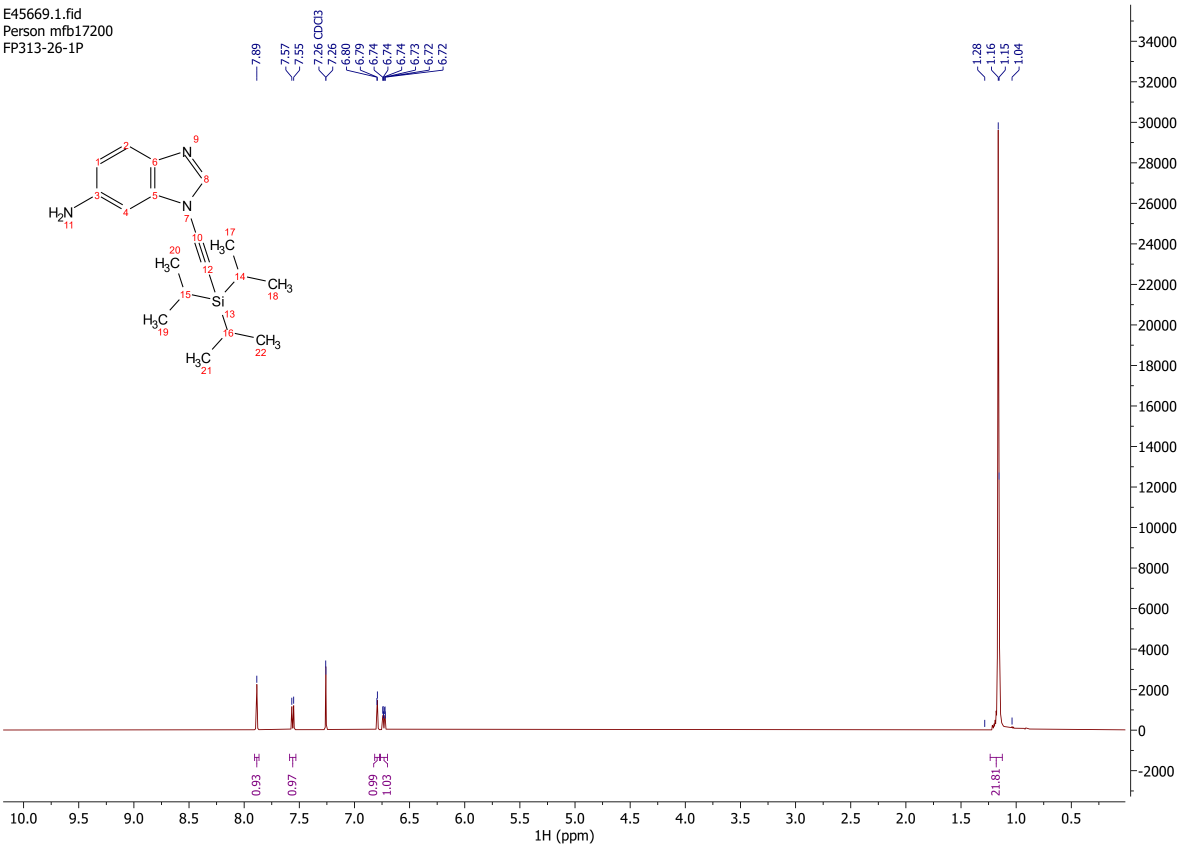

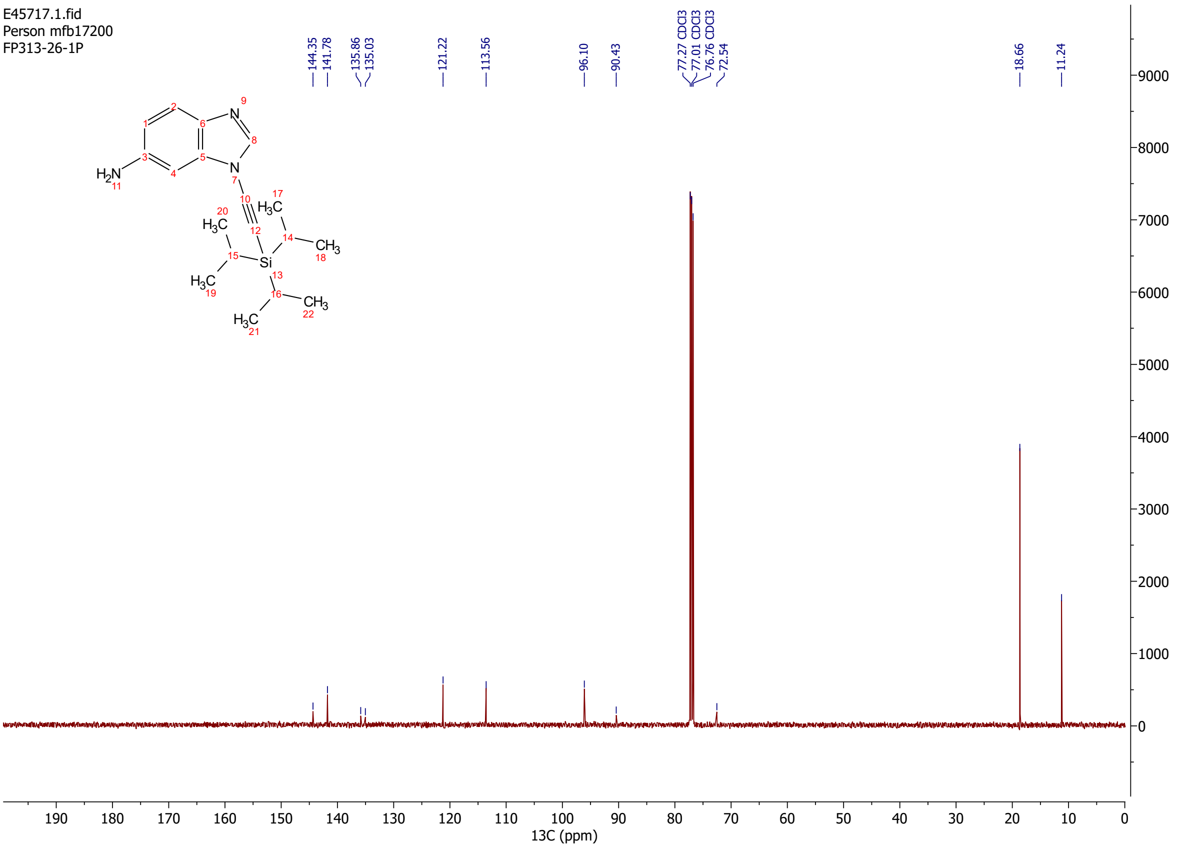

NMR spectra of compound **S3**

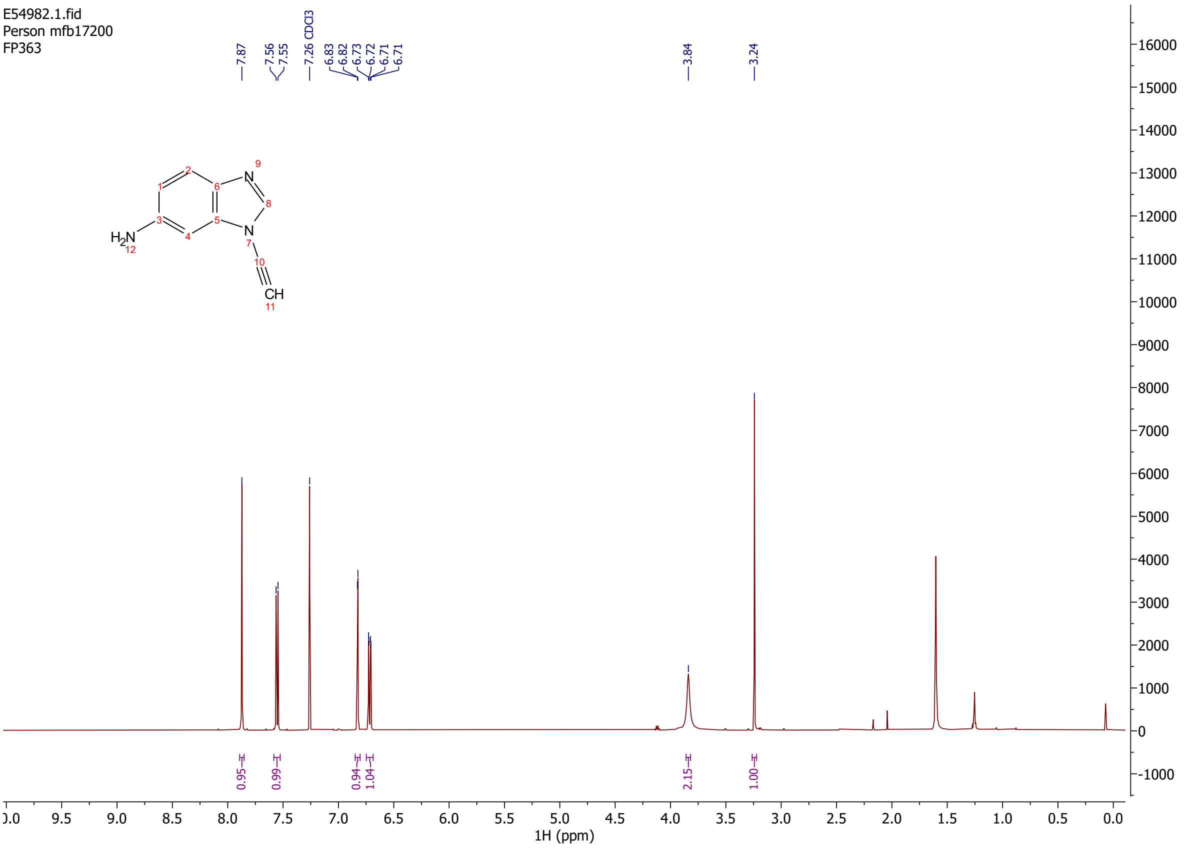

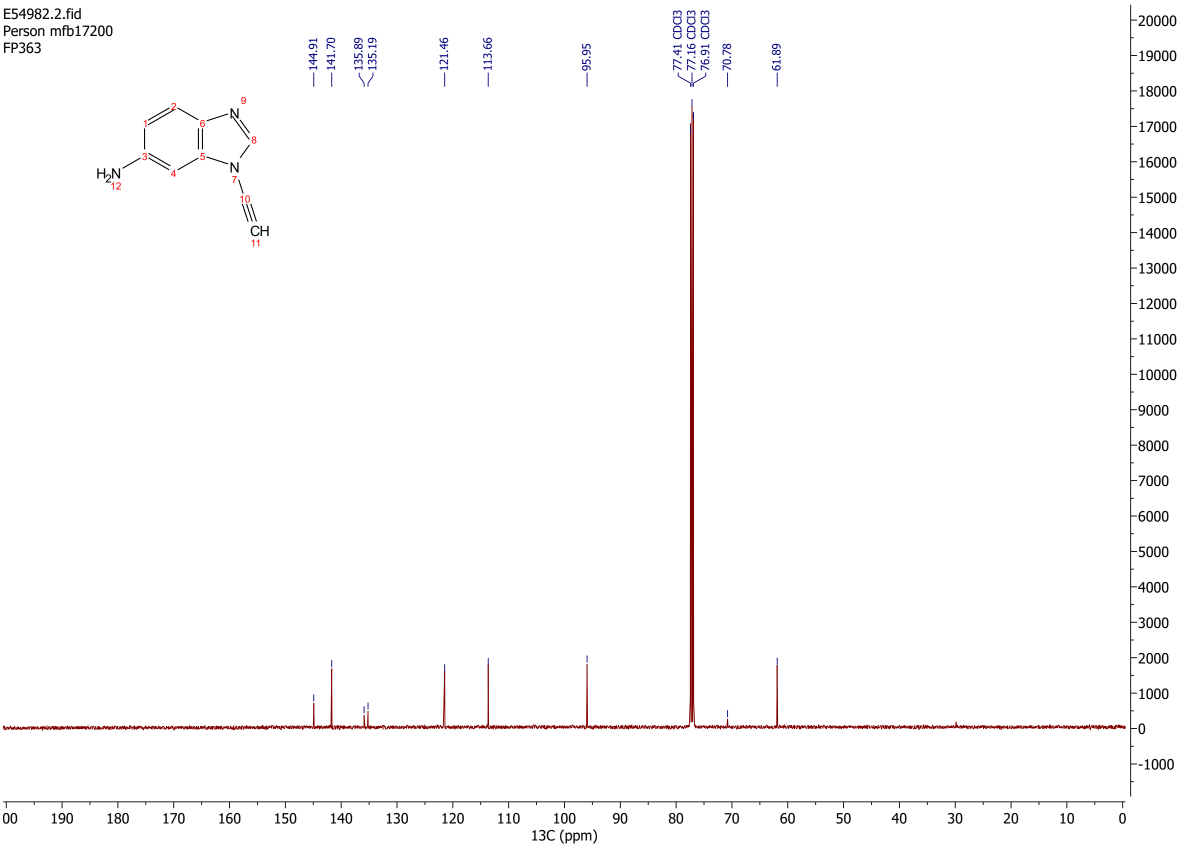

NMR spectra of compound **8**

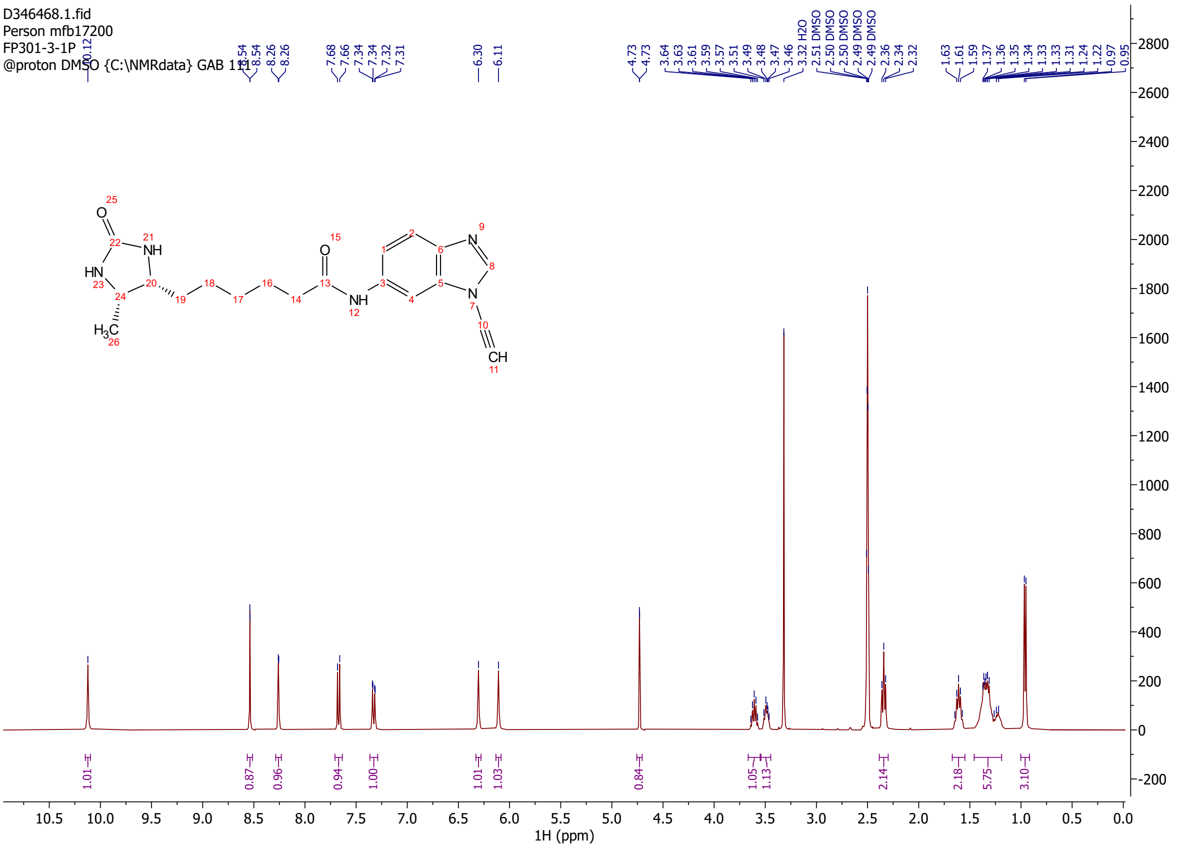

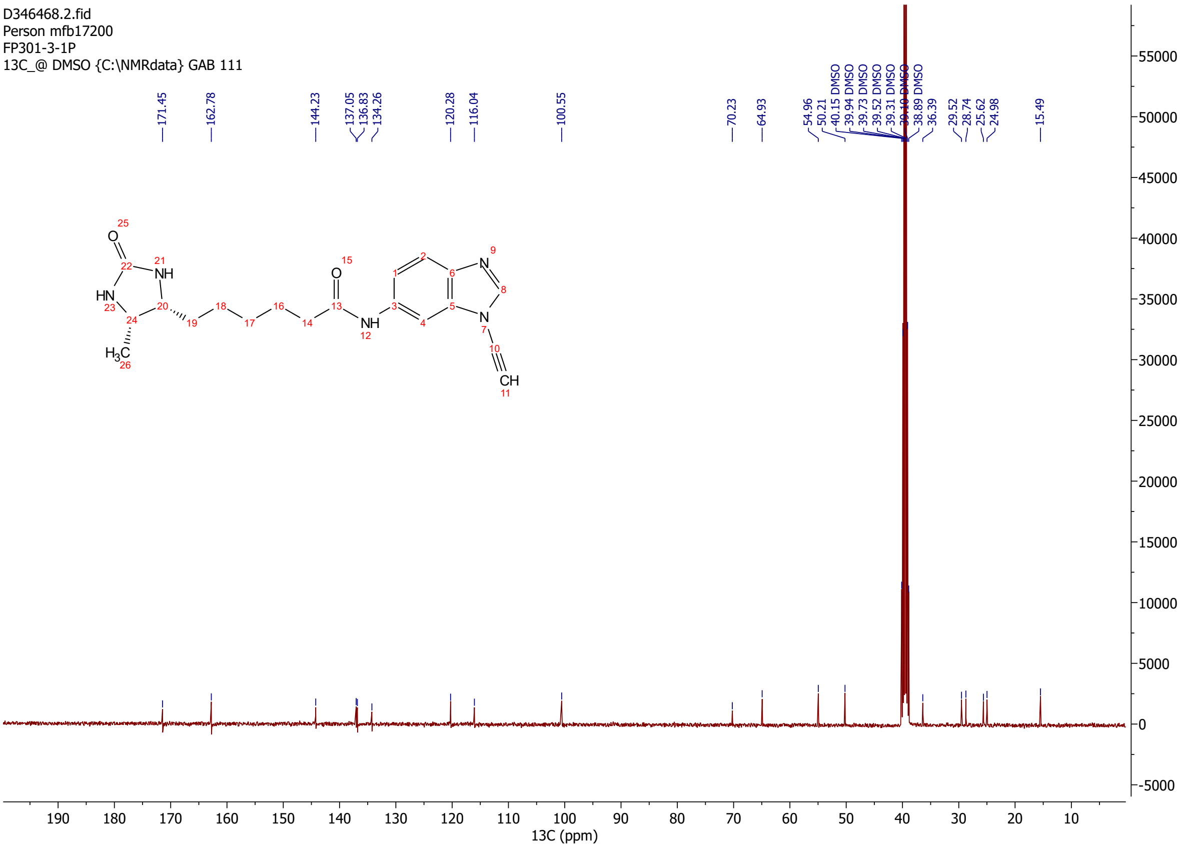

NMR spectra of compound **S5**

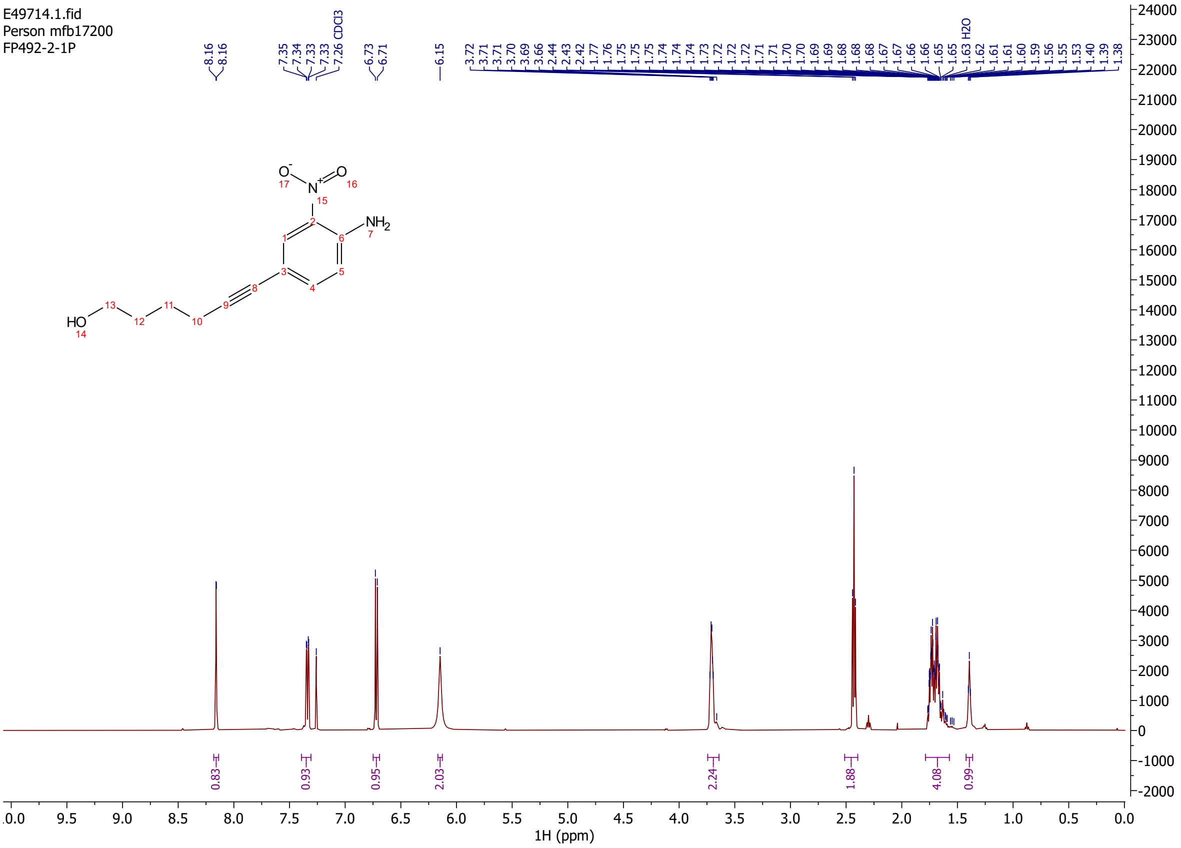

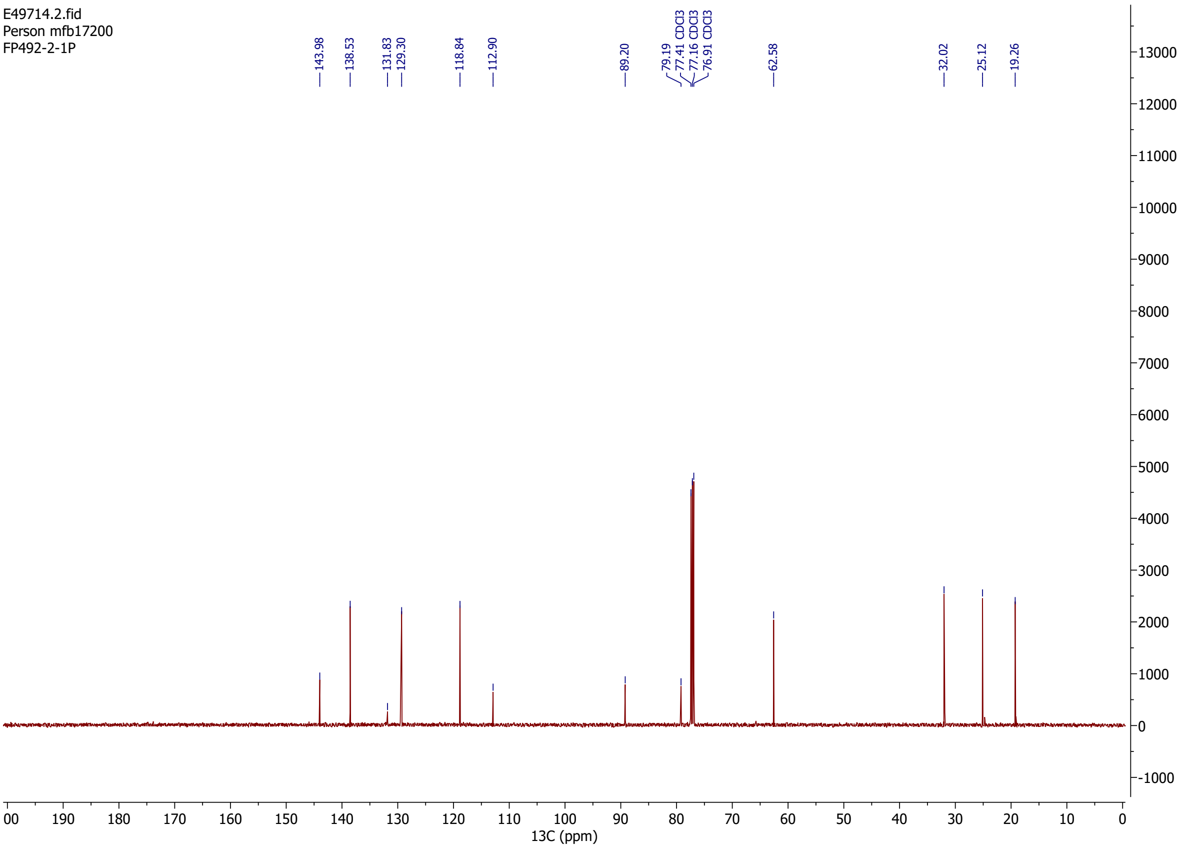

NMR spectra of compound **S6**

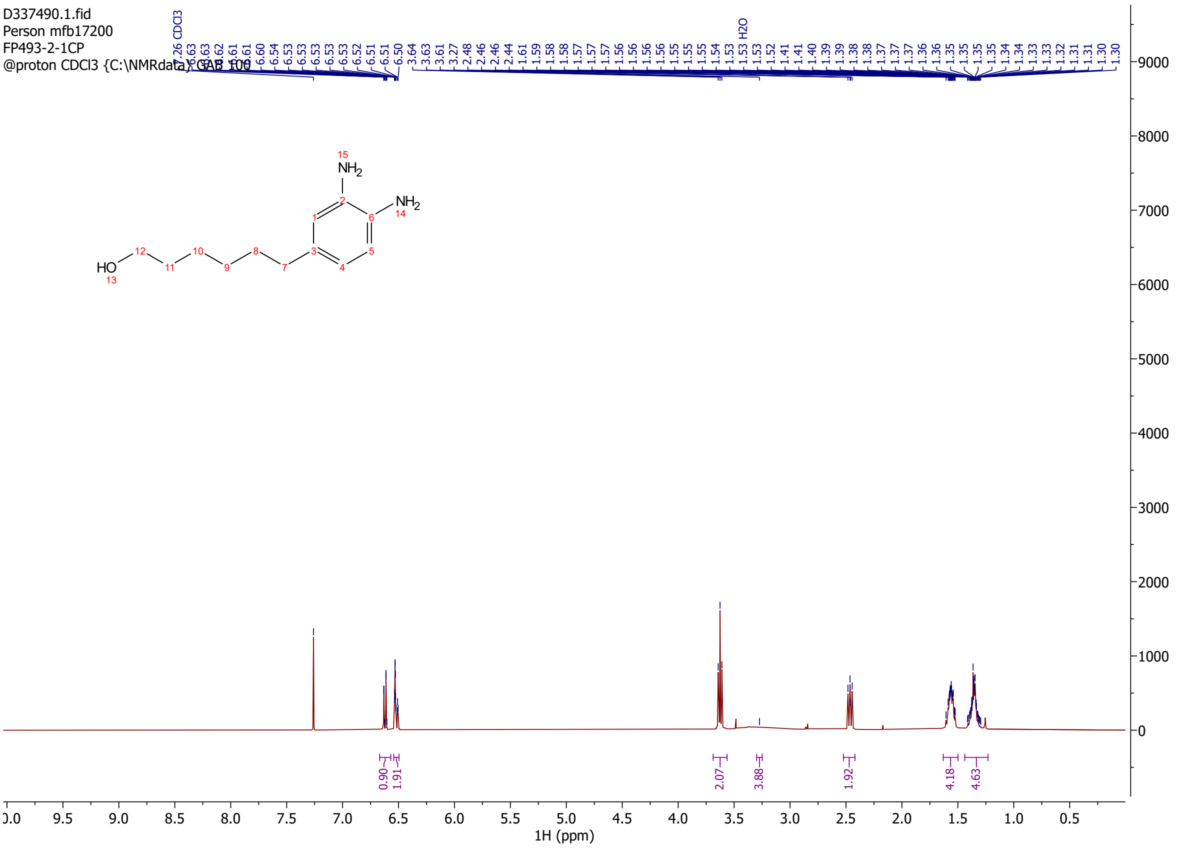

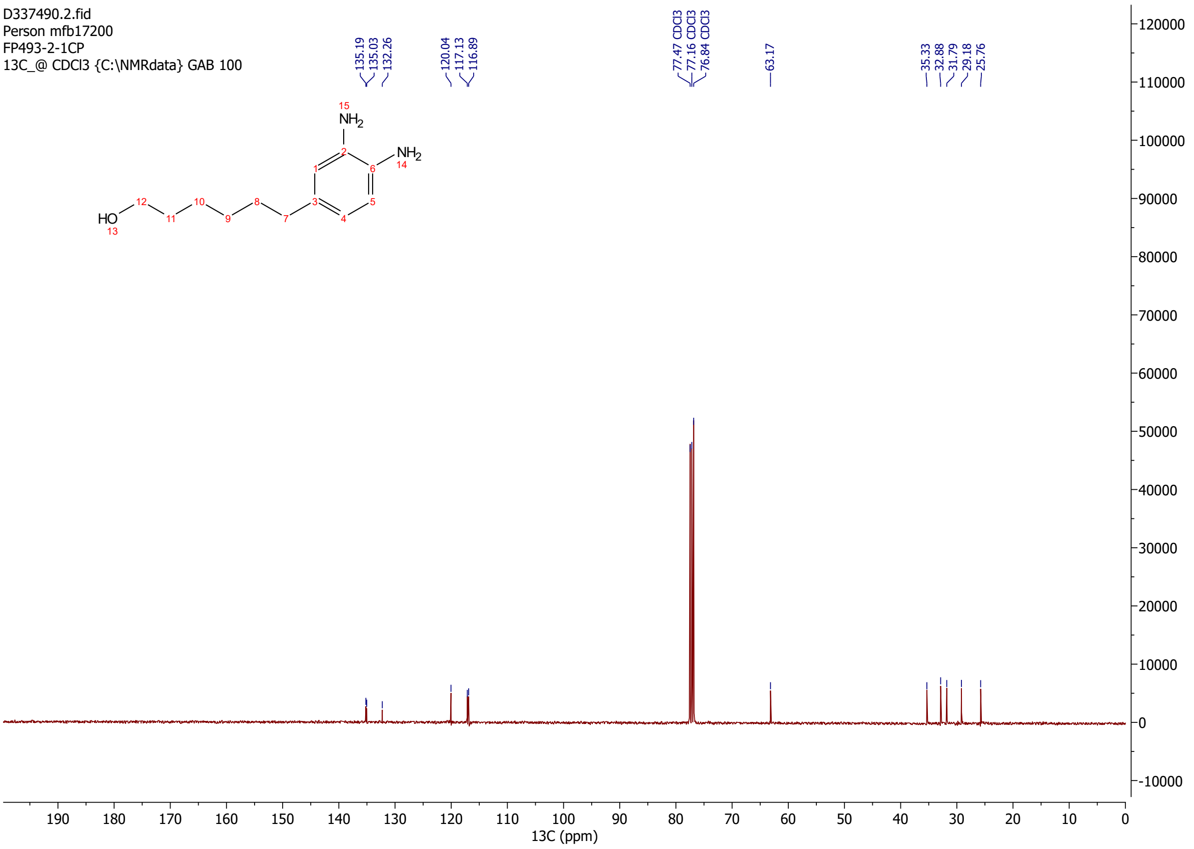

NMR spectra of comound **S7**

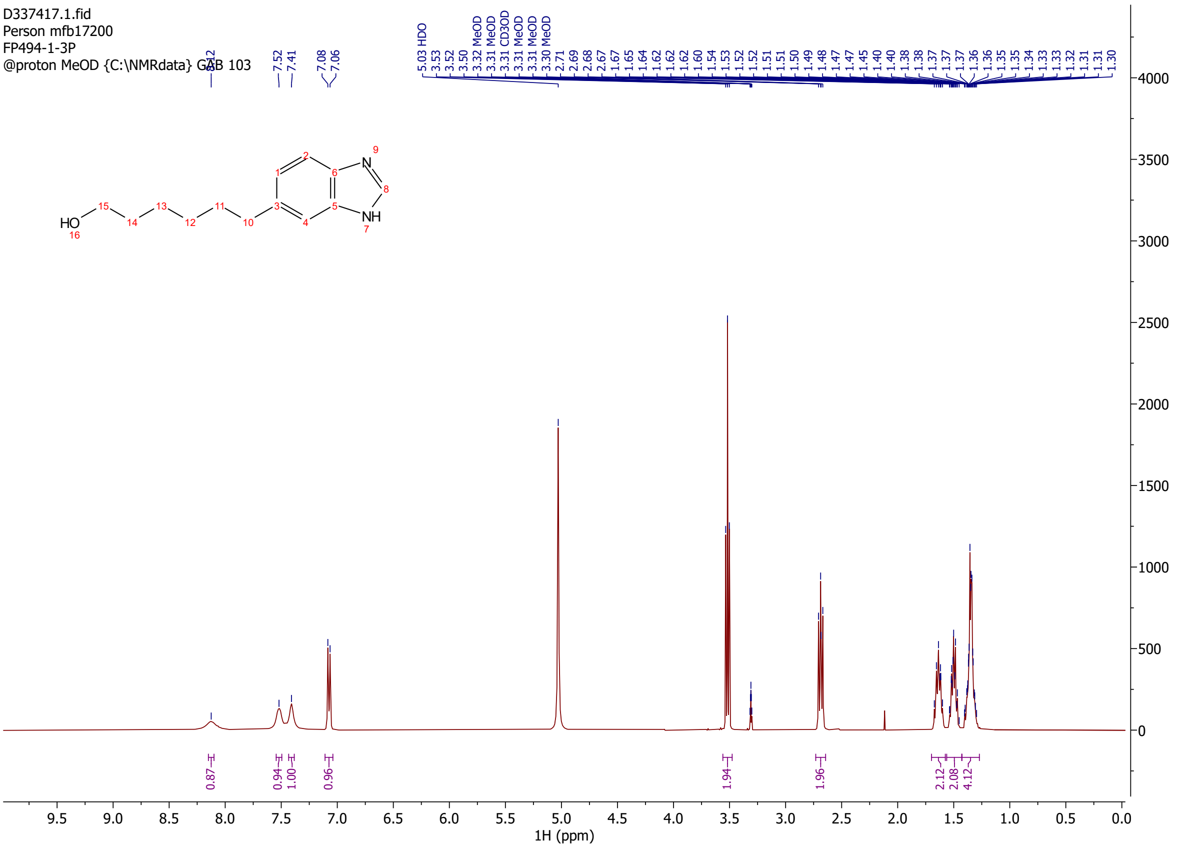

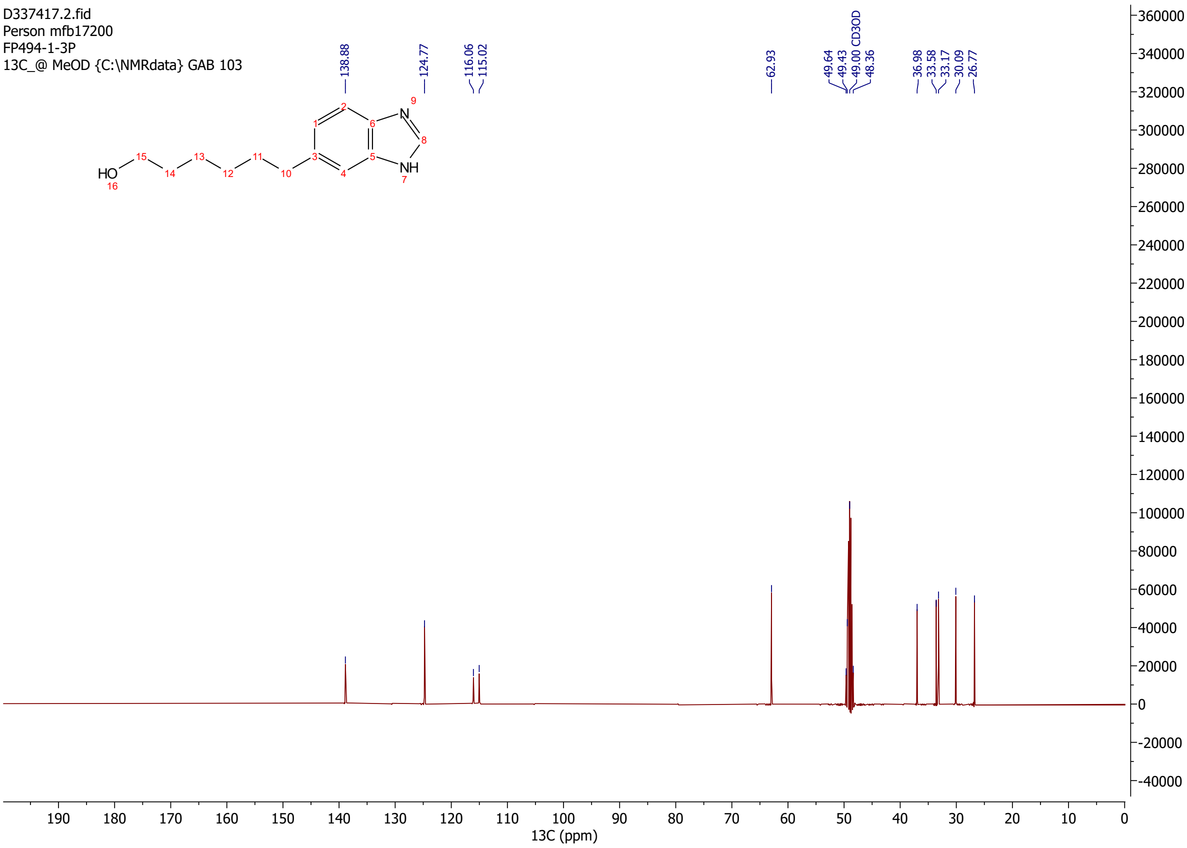

NMR spectra of compound **S8**

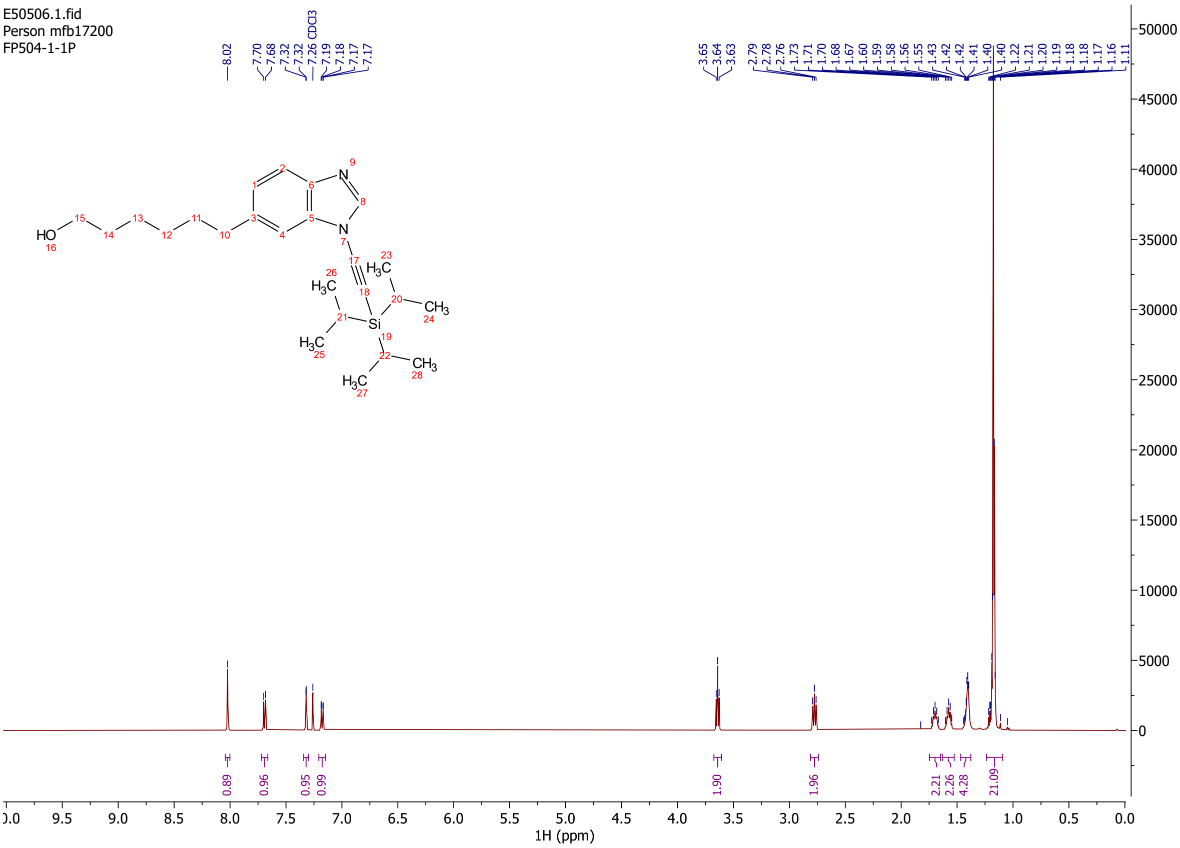

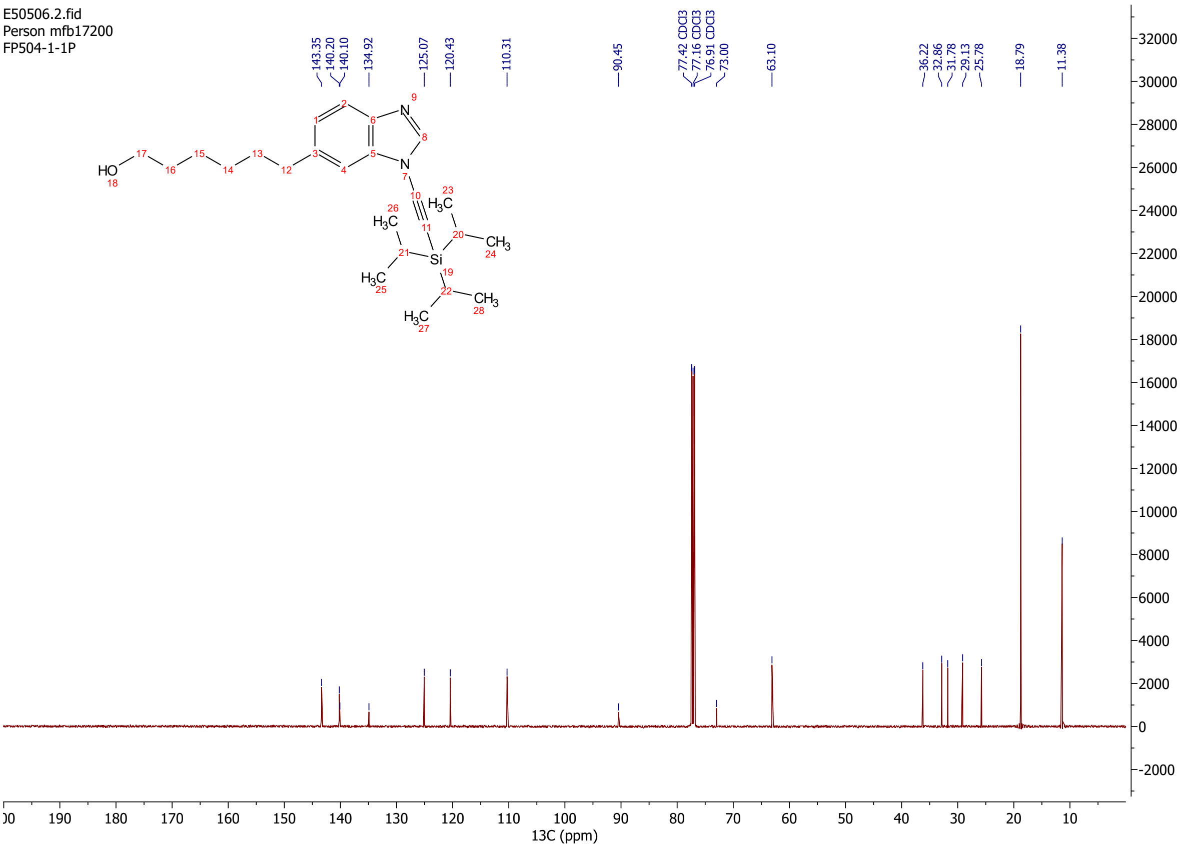

NMR spectra of compound **S9**

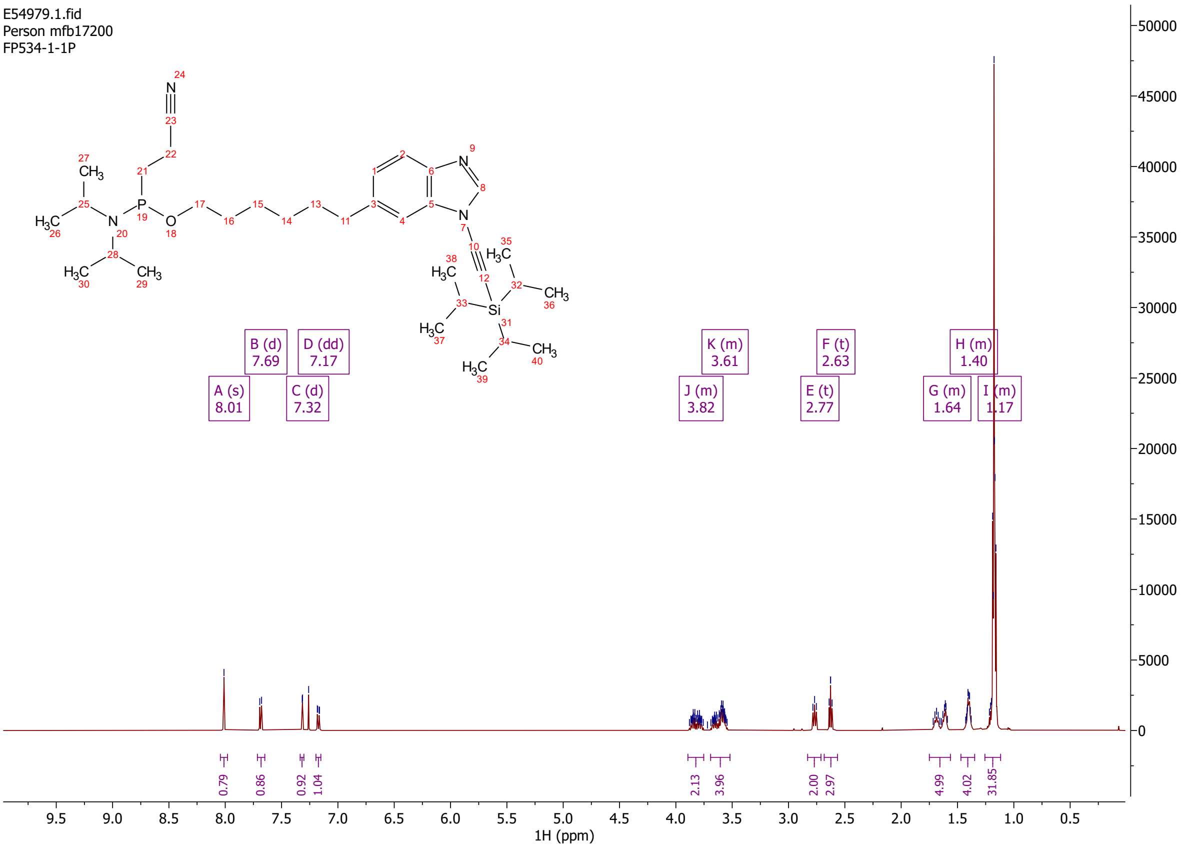

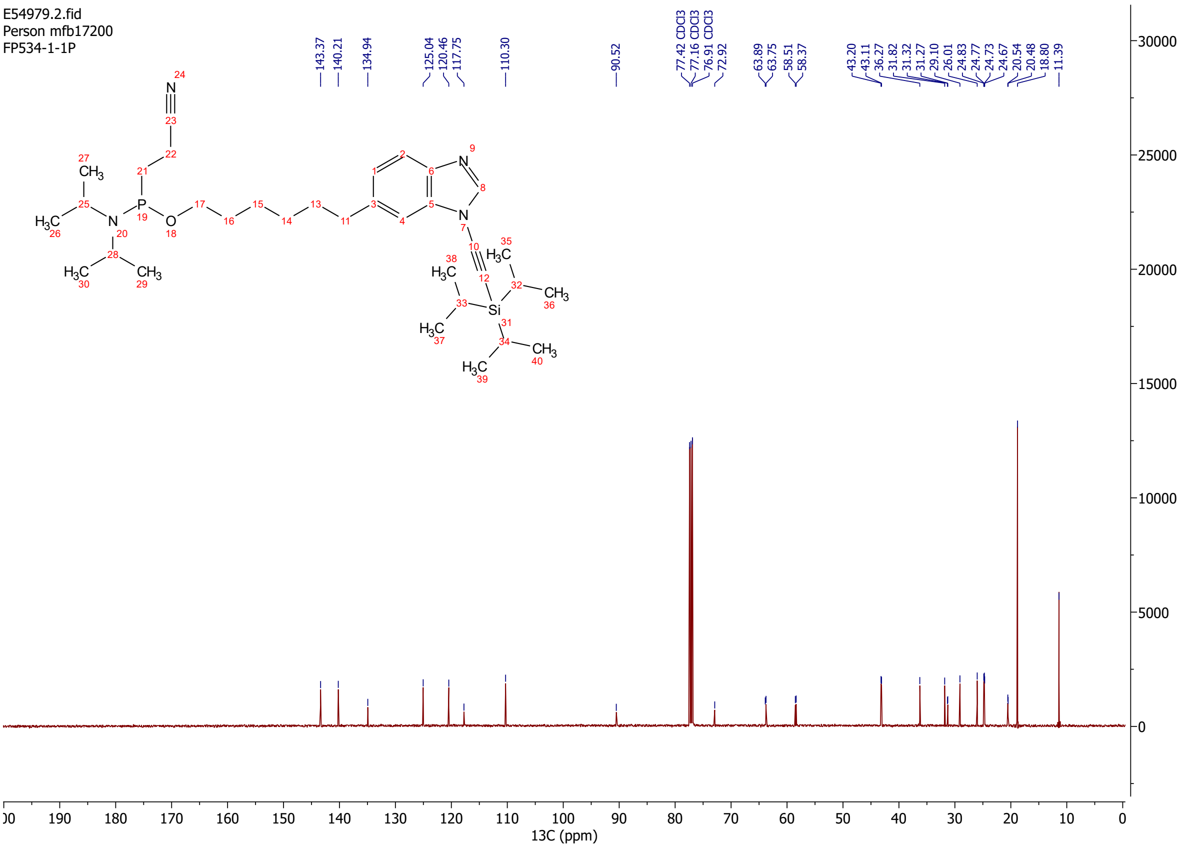

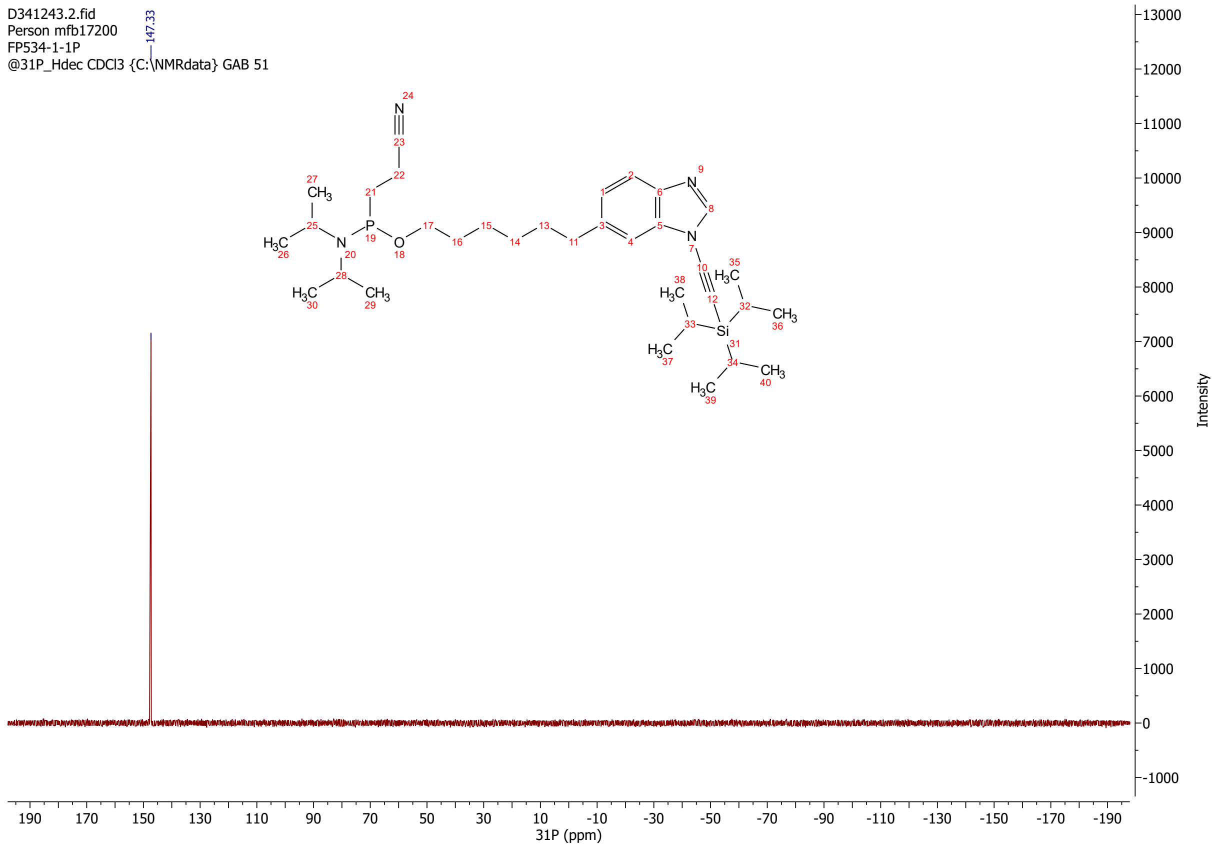
D341243.2.fid
Person mfb17200
FP534-1-1P
@31P_Hdec CDCl3 {C:\NMRdata} GAB 51
147.33
31P (ppm)
Intensity

5. List of References

1 G. A. Burley, D. L. Davies, G. A. Griffith, M. Lee and K. Singh, *J. Org. Chem.*, 2010, **75**, 980–983.

2 C. P. Seath, G. A. Burley and A. J. B. Watson, *Angew. Chemie Int. Ed.*, 2017, **56**, 3314–3318.

3 A. H. Banday, S. A. Shameem, B. D. Gupta and H. M. S. Kumar, *Steroids*, 2010, **75**, 801–804.

4 A. Nocentini, M. Ferraroni, F. Carta, M. Ceruso, P. Gratteri, C. Lanzi, E. Masini and C. T. Supuran, *J. Med. Chem.*, 2016, **59**, 10692–10704.

5 P. Conti, L. Tamborini, A. Pinto, L. Sola, R. Ettari, C. Mercurio and C. De Micheli, *Eur. J. Med. Chem.*, 2010, **45**, 4331–4338.

6 P. Kleiner, W. Heydenreuter, M. Stahl, V. S. Korotkov and S. A. Sieber, *Angew. Chemie Int. Ed.*, 2017, **56**, 1396–1401.

7 A. Haslop, A. Gee, C. Plisson and N. Long, *J. Label. Compd. Radiopharm.*, 2013, **56**, 313–316.

8 H. Mach, C. R. Middaugh and R. V. Lewis, *Anal. Biochem.*, 1992, **200**, 74–80.
